# Heroin addiction modulates transcription factor binding in regulatory regions of the human putamen

**DOI:** 10.1038/s41598-026-52754-7

**Published:** 2026-05-12

**Authors:** Rajashree Chakraborty, Avinash Veerappa, Chittibabu Guda

**Affiliations:** Department of Genetics, Cell Biology and Anatomy, University of Nebraska Medical Center, Omaha, NE USA

**Keywords:** Opioid use disorder, Transcription factors, ATAC-Seq, Neuroinflammation, Putamen, Substance use disorders, Neurology, Neuroscience

## Abstract

The ongoing opioid crisis underscores the need to elucidate the neurobiology of addiction for improved treatments. Dynamic transcription factor (TF) binding is a key driver of substance use disorders (SUDs), yet its genome-wide patterns in human brain cell types remain poorly defined. We performed a computational re-analysis of publicly available ATAC-seq data (PRJNA561094) from post-mortem putamen samples of heroin users and matched non-users, where we profiled chromatin accessibility analyzing neuronal and glial cells separately. Genome-wide TF binding sites were identified and quantified, followed by footprinting and differential binding analyses. Co-occurrence analysis was applied to identify TF pairings associated with SUD-related genes. Neurons from heroin users exhibited 38 TFs with altered binding and footprinting, while glia showed 11 differentially bound TFs, primarily from the FOS**,** JUN, and ZNF families. NRF1 and KLF15 were differentially bound in both cell types. These TFs were linked to SUD-associated genes and activation of neuroinflammatory, neurohormonal, and S100 pathway genes. Co-occurrence analysis identified five TF pairs in user neurons and thirteen in non-user glia interacting with SUD-related genes and driving neuroinflammatory signaling. Overall, our findings reveal distinct alterations in TF binding accessibility and co-occurrence networks in heroin users’ neurons and glia, implicating transcriptional dysregulation in addiction-related neuroinflammation and neurohormonal activity.

**Supplementary Information:**

The online version contains supplementary material available at 10.1038/s41598-026-52754-7.

## Introduction

Opioid use disorder (OUD) is characterized by an overwhelming urge to consume opioids, including heroin, morphine, codeine, fentanyl, and oxycodone, resulting in significant psychosocial impairments^1,2^. Current mortality rate due to heroin overdose is 5.5 deaths per 100,000 people for men compared to 2.0 deaths for women^3^. Growing evidence highlights the critical role of epigenetic regulations in SUDs, including OUD, with TF binding dynamics emerging as key molecular contributors^4–8^. Studies have demonstrated that opioid use alters the function of TFs responsible for remodeling chromatin structures and modulating DNA accessibility within the regulatory regions of the brain’s reward circuitry^6,9^. Previous research has highlighted the involvement of TFs in regulating opioid-associated genes and elucidated its importance in the overall pathophysiology of OUD^10–15^. Changes in gene expression associated with OUD in both neural and non-neural cells can lead to stable alterations in neural circuitry, potentially resulting in altered behavior such as addiction cycle phenotypes and increased susceptibility to substance use^15–17^. Therefore, understanding the TF binding dynamics in OUD is crucial for grasping the complexities of its pathophysiology.

Prior substance use studies utilized ATAC-seq across various brain regions and models and identified distinct epigenetic and gene expression changes including hyperacetylation, altered signaling, metabolic, and cell-type-specific regulatory modifications, and have uncovered potential therapeutic targets to mitigate addictive behaviors^18–22^. A significant limitation in the field is the predominance of studies using animal models, with limited studies on human samples^23^. This gap highlights the importance of post-mortem studies using age- and gender-matched users and non-users to gain a more accurate and unbiased understanding of the chromatin states in OUD. Moreover, researching addiction in the putamen region of brain is particularly important because of its central role in habit formation and directly influencing compulsive drug-seeking behaviors, thereby providing critical insights into the neural mechanisms of addiction that are especially relevant for developing targeted interventions^24^. To date, no study has explored differences in TF binding in putamen of post-mortem samples from individuals with a history of heroin overdose. To achieve this, we conducted a comprehensive assessment of chromatin accessibility by performing a computational re-analysis of publicly available genome-wide ATAC-seq data (PRJNA561094, Egervari et al., 2020) from post-mortem putamen samples of heroin users and matched non-users. We employed ATAC-seq to examine neuronal and glial cells, focusing on TF footprinting, and co-occurrence patterns between heroin-users and non-users. Our analysis identified 38 and 11 differentially bound TFs in neuronal and glial cells of users versus non-users, respectively, with the majority belonging to the FOS, JUN, and ZNF families. Further, unique TF binding sites in heroin-users and non-users, target genes and downstream regulatory interactions were identified. Additionally, we predicted 5 and 13 co-occurring TFs in the user neurons and the non-user glial cells, respectively, evaluated binding differences, and assessed cell-specific variations in their presence, elucidating how these TF interactions relate to SUD-associated gene networks. Our findings indicate altered TF binding accessibility in both neuronal and glial cell types of users compared to non-users, and consequently, modified neuroinflammatory, neurohormonal, and S100 pathways. Thus, this study investigates how heroin use modifies TF binding in neurons and glial cells, enhancing our understanding of addiction mechanisms and helping identify potential therapeutic targets.

## Methods

### Raw data acquisition

Human brains from a homogenous cohort of Caucasian subjects with a history of heroin overdose and control non-users (determined by self-report and ancestry-informative marker analysis) were collected at autopsy within 24 h of death at the Department of Forensic and Insurance Medicine, Semmelweis University, Hungary by original authors^17^. As per the original study, informed consent from legally authorized representatives was obtained at the time of autopsy, and cause of death was determined by the forensic pathologist. All heroin users (70% male, 30% female; aged 19-31 years) had a documented history of chronic heroin use (Supplementary Table 1) and died from heroin overdose, meeting the inclusion criteria of documented heroin use and/or positive toxicology at death, along with physical evidence of intravenous drug use. Multi-drug users, individuals receiving methadone/buprenorphine treatment, and those with head trauma or comorbid psychiatric diagnoses were excluded. The non-user cohort (80% males, 20% females; aged 1931 years) had negative toxicology, no history or evidence of opiate or other drug use, no head trauma, and no documented psychiatric disorders. Causes of death for non-users included respiratory insufficiency from pneumonia, acute cardiac failure (various etiologies), food asphyxiation, natural causes, electrocution, and similar events. Univariate correlations for demographic variables (age, pH, gender, etc.) were explored, and significant variables were included in the final model. Putamen tissue punches were obtained from the same coronal plane.

ATAC-Seq was performed on these post-mortem putamen samples from individuals who died of heroin overdose that were matched based on age, gender and race with non-user subjects. ATAC-Seq reads in FastQ format were obtained from the Sequence Read Archive (SRA) under accession ID PRJNA561094^18^. The study included heroin users (n = 10) and non-user controls (n = 10), where neuronal and glial cell samples were obtained from the putamen region and sequenced separately from each subject in two technical replicates. Hence, the experimental design includes 20 total subjects, with two cell types per subject and two technical replicates of each cell type making a total of 80 ATAC-seq samples. Hence our experimental design contains 20 total subjects (10 heroin users + 10 non-users) × 2 cell types (10 glia samples + 10 neuron samples) × 2 technical replicates = 80 total study samples.

### Quality control

Quality control (QC) and alignment metrics were performed as part of the pre-analysis step. *FastQC 0.11.9* was used to perform pre-alignment QC, read alignment steps and visualize base quality scores, GC content, sequence of length distribution, sequence duplication levels, k-mer overrepresentation, and contamination of primers and adapters in the raw sequence data^25^. The sequencing and experimental batch metrics of all samples were homogenous. Using *Trim Galore! V0.6.10*^26^*,* 13 bp sequencing adapters were removed along with low-quality bases for accurate read alignment. Raw FastQ files and accompanying ATAC-Seq metadata were retrieved using the *nf-core/fetchings* pipeline v1.4 on *Nextflow* v21.04.0^27^. The pipeline facilitated the extraction of sample IDs from the study, which were subsequently converted into experiment-level IDs compatible with the European Nucleotide Archive (*ENA*) Application Programming Interface (*API*). Comprehensive metadata, including FastQ file download links, was extracted via the *ENA API* to ensure accurate sample tracking and management. To download the FastQ files, *curl* was utilized, followed by integrity verification using *md5sum*. This two-step process ensured the accuracy and reliability of the sequencing data by confirming that the downloaded files matched their original checksums. A unified sample sheet was then compiled, encompassing metadata for all samples alongside the file paths to their respective FastQ files. This compilation was essential for the subsequent determination of open chromatin accessibility regions.

### Alignment and processing

The trimmed reads were mapped to the *GRCh38* human reference genome using *BWA v0.7.17-r188*^28^. BWA achieved unique mapping rates exceeding 80% by allowing overhangs of bases on both ends of the reads. To locate and remove duplicates, the *MarkDuplicates* tool from Picard v2.23.1^29^ was employed, which also performed QC metrics for ATAC-Seq, including the merging of alignments from different libraries of the same sample. Duplicates were relabeled using *Picard v2.23.1*, after which reads mapped to mitochondrial DNA were removed using *Samtools* v1.10^28^, followed by mapping to blacklisted regions using *Samtools v1.10* and *Bedtools v2.29.2.* Subsequent filtering steps involved removing reads mapped to blacklisted regions using *Samtools* v1.10 and *Bedtools* v2.29.2^30^. Reads marked as duplicates, those not designated as primary alignments, unmapped reads, or reads mapped to multiple locations were filtered out using *Samtools v1.10*. Additionally, reads containing more than four mismatches, those that were soft-clipped, or had an insert size exceeding 2 kb were removed using *BamTools v2.5*^31^. Reads mapped to different chromosomes, those not in the forward read orientation, or those representing only one read of a pair were corrected using *Pysam v0.15.3. Picard v2.23.1 and Preseq v2.0.3*^32^ were used to conduct alignment-level quality non-user (QC) and assess library complexity. BigWig files normalized to 1 million mapped reads were generated using *Bedtools v2.29.2* and *bedGraphToBigWig*^30^. Genome-wide enrichment was calculated with *deepTools v3.4.3*^33^. Chromatin accessibility, represented as broad and narrow peaks, was identified using *MACS v2.2.7.1*^34^, which applies a Poisson distribution model to estimate fragment distribution. Peaks were annotated to gene features via *Homer v4.11*^35^, and *Bedtools v2.29.2* was employed to generate consensus peak sets across all samples. Read counts within these consensus peak sets were extracted using *featureCounts v2.0.1*^36^. Finally, differential accessibility analysis, principal component analysis (PCA), and clustering were carried out using *R v3.6.2* and *DESeq2 v3.15*^37^.

### Differential analysis and peak annotation

Differential peak analysis and annotation were conducted using *UROPA*^38^. *UROPA* facilitated the identification of candidate regions via consensus peaks, normalization of fragment counts in these regions, and statistical comparison with non-users. Peaks were obtained using *MACS2 v2.2.7.1*^34^ and peak annotation related chromatin accessibility to gene regulation using *Homer v4.11*^35^.

### Footprinting analysis

TF Occupancy Prediction by Investigation of ATAC-Seq Signal (TOBIAS)^39^ as employed to perform a genome-wide investigation of TF binding dynamics, establishing a comprehensive footprinting framework. TOBIAS integrates multiple tools to analyze TF binding from ATAC-Seq signals by correcting Tn5 sequence bias, adjusting for sequence-specific biases introduced by the Tn5 transposase; calculating footprint scores by assessing the depletion of Tn5 cut sites within TF binding motifs based on chromatin accessibility and sequencing depth; detecting TF binding events by integrating footprints and known TF motifs; and identifying TF binding site (TFBS) positions from given sequences and motifs, followed by clustering motifs based on similarity. This comprehensive approach allows for the prediction of genome-wide TF binding by integrating footprints with genomic information and TF binding motifs.

### TF-COMB analysis

TF-COMB (Transcription Factor Co-occurrence using Market Basket Analysis)^40^ was utilized to predict co-occurring TF pairs based on cosine similarity and z-scores. The analysis involved filtering co-occurrence measures using the above filters to select rules of interest, annotating TF binding sites near the promoters of open chromatin regions and performing a comparative analysis between cases (heroin-users) and controls (non-users) to identify differentially co-occurring TF pairs. Visualization of the filtered TF pairs was achieved using heatmaps generated with the heatmap function in R. Additionally, an orientation analysis was conducted to understand the spatial relationship between co-occurring TF pairs, determining if pairs were on the same or opposite strands, and refining this analysis by assessing the relative order of TF1-TF2 co-occurring pairs, which resulted in “convergent” and “divergent” dimensions. The locations of TF pairs were measured, and footprints were plotted by calculating TF positions from motifs. Heatmaps of all binding pairs, sorted by distance, were created using *PairMap, PairTracks, and PairLines*. This analysis elucidates the complex interactions and spatial relationships between TFs, providing insights into their collective roles in regulating gene expression in heroin-users compared to non-users.

### Co-ordinate extraction, unique/common, annotation

Chromosomal coordinates were extracted, and unique as well as common TFs from the bound TF coordinates were identified using in-house python scripts (Supplementary Fig. 1) that can be accessed at https://github.com.mcas.ms/GudaLab/TF-regulatory-region-annotation. These coordinates were then annotated using the EnhancerAtlas database (http://www.enhanceratlas.org/).

### Ingenuity pathway analysis (IPA)

Target genes identified from differentially bound TF binding sites in neuronal and glial cohorts were submitted to Ingenuity Pathway Analysis (*IPA*, Qiagen) for upstream regulator analysis and canonical pathway analysis. Genes annotated as SUD-associated were identified using *IPA*'s built-in ‘Diseases and Functions’ category for ‘Substance-related disorders’. Network interactions between differentially bound TFs, their upstream transcriptional regulators, and SUD-associated genes were visualized using *IPA*'s pathway visualization tool. A Benjamini–Hochberg corrected p-value threshold of < 0.05 was applied to identify significantly enriched pathways and upstream regulators. The *IPA* Knowledge Base (QIAGEN, accessed 2024) was used as the reference dataset for all analyses.

## Results

### Differential TF binding patterns in neuron and glia of heroin users and non-users

**Fig. 1:**
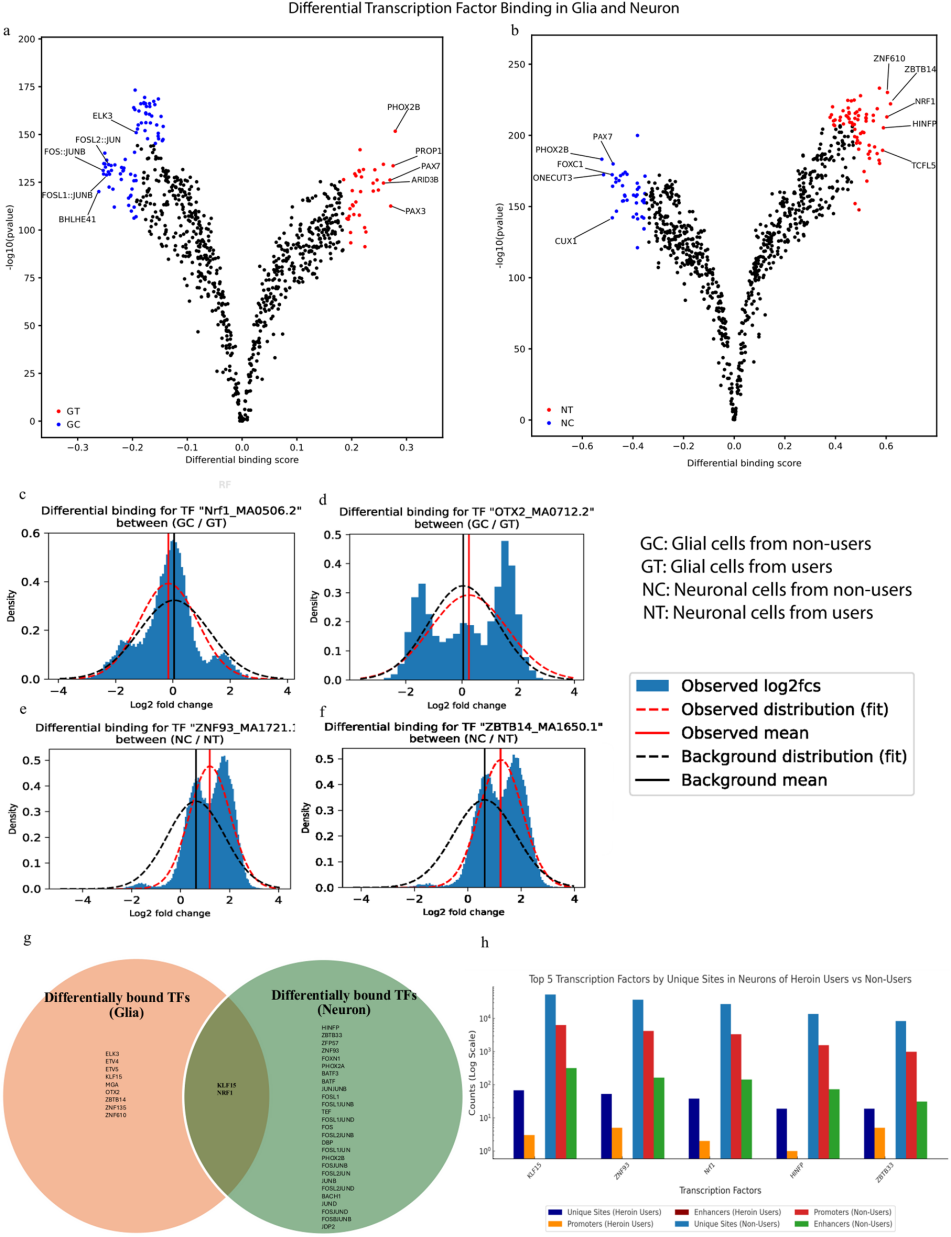
Comparative analysis of differential TF binding is user and non-user glia and neuron (**A**, **B**). Scatter plot showing differential TF binding between user and non-user groups in glia and neuron (GT: glia of users, GC: glia of non-users, NT: neurons of users, NC: neurons of non-users). Each dot represents a TF, with the x-axis showing the differential binding score and the y-axis the -log10(p-value). Red dots indicate TFs with higher (significant) binding scores in users, blue dots indicate the same for non-users. Black dots indicate non-significant differences. The top 5 differentially bound TFs on either side of the volcano plot are labelled. (**C**-**F**) Density plots of log₂FC for subset of TFs (NRF1, OTX2, ZNF93 and ZBTB14) that showed differential binding across user and non-user groups. (**G**) Differentially bound TFs found in glia and neuron after filtering with 1^st^ and 99^th^ percentile cutoff and p-value < 0.05. (**H**) Comparison of top 5 TFs by unique binding sites, promoters, and enhancers in neurons of heroin users vs non-users.

We conducted an analysis of the putamen region in post-mortem samples from heroin users and matched non-user subjects (Supplementary Table 1) to identify and elucidate differences in TF binding patterns associated with heroin-related alterations and their effects in neurons and glial cell types. To determine a specific TF binding score for each subject, we combined the footprint scores with binding motif information using the *BinDetect* module from *TOBIAS* (methods). We utilized the 841 known annotated TFs with motifs from the JASPAR database^41^. The log₂ fold change (log₂FC) in binding score, or differential binding score (DBS) for each TF was calculated as the ratio of the mean binding scores between users and non-users to evaluate differential TF binding between the two groups. Volcano plots (Fig. 1A,B) illustrate the differential TF binding scores, represented as log₂FC for glial cells and neurons in heroin users compared to non-users. Blue and red colors denote clusters of TFs with negative and positive DBS, respectively. In the glial cohort, we observed a positive DBS for 377 TFs, ranging from 0 to 0.2, while a negative DBS for 463 TFs, spanning from 0 to -0.2 (Supplementary Table 2). Similarly, within the neuronal cell type, negative DBS was observed for 397 TFs between 0 and -0.5, whereas positive DBS was observed for 443 TFs ranging from 0 to 0.2 (Supplementary Table 3). These results suggest an association between heroin use and altered inferred TF binding patterns. After applying filtering criteria p-value < 0.05 and DBS thresholds of 1st and 99th percentile, 11 TFs were identified in the glial cohort with differential binding (Supplementary Table 4, Supplementary Fig. 2A), including 8 with positive DBS and 3 with negative DBS. Among these, NRF1 (Fig. 1C) displayed the highest DBS with a log₂FC of 0.225, while OTX2 (Fig. 1D) exhibited the lowest DBS score with a log₂FC of -0.168. In contrast, we identified 38 TFs in the neuronal cell type (Supplementary Table 5, Supplementary Fig. 2B) at the 1st and 99th percentiles which showed more TFs with 27 positive log₂FC scores DBS and 11 negatives. The highest DBS was observed for ZNF93 (Fig. 1E), with a log₂FC of 0.247, while the lowest score was recorded for ZBTB14 (Fig. 1F) with a log_2_FC of -0.612.

### Annotating unique TF binding sites

Subsequently, we investigated the unique TF binding chromosomal sites in both non-user and heroin-user cohorts to elucidate the transcriptional regulatory elements specific to these regions. We found unique chromosomal sites for 10 TFs in glia and 29 TFs in neurons of heroin users and non-users (Supplementary Tables 6 and 7). In both glial and neuronal cell types, a substantially smaller proportion of unique regulatory regions was observed in users compared to non-users. This reduction may be attributable to the reduced availability of open chromatin regions in the OUD cohorts (Supplementary Table 6). Upon annotation of all the unique TF bound regions, we determined that majority of the regulatory regions were promoters, with the remaining regions classified as enhancers in both non-user and user cohorts across neuronal and glial cell types (Fig. 1H; Supplementary Fig. 2C).

Furthermore, we identified 2 differentially bound TFs, NRF1 and KLF15, that were common to both neuronal and glial cell types (Fig. 1G). We analyzed the differences and similarities in NRF1 and KLF15 bound coordinates across both cell types. A similar trend of unique genomic regions was discerned in the non-user and user cohorts across neurons and glia as previously observed. Additionally, we detected 11,890 and 4,786 common genomic regions for non-users and users across neurons and glial cells for KLF15 and NRF1, respectively (Supplementary Table 8). Similarly, annotation of these common genomic regions across cell types revealed that the majority were promoters, with the remainder being enhancers. In conclusion, we observed differential TF binding and a reduced number of unique genomic regions (which are predominantly promoters) in users compared to non-users in both neuronal and glial cohorts. These findings may reflect the effects of heroin addiction, which could ultimately impact TF binding and dysregulate downstream transcriptomic pathways.

### Increased TF footprinting in heroin users

**Fig. 2:**
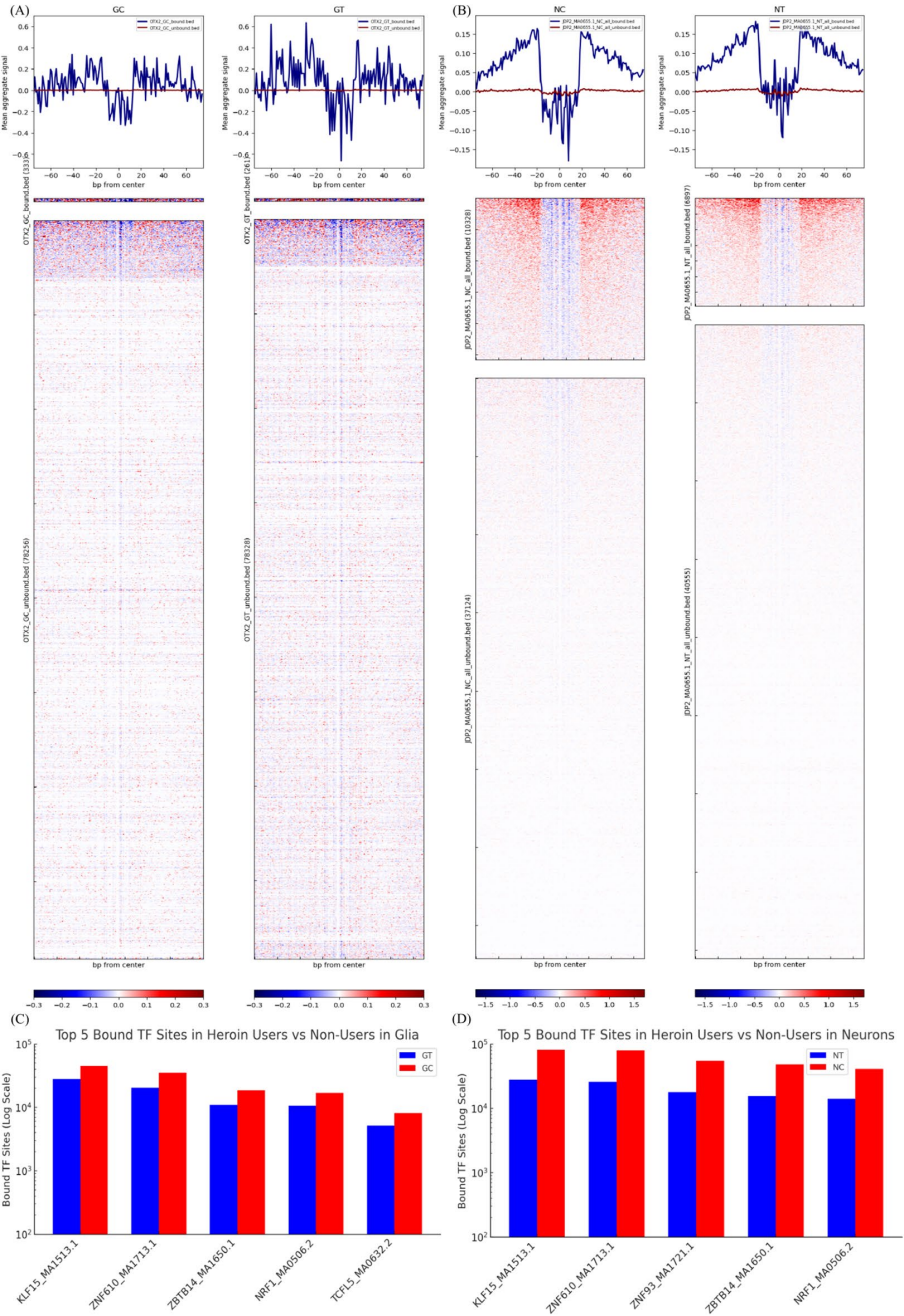
TF differential footprinting in user and non-user glia and neurons. (**A**, **B**) Heatmaps of TF footprinting for OTX2 and JDP2 in user and non-user groups in glia and neuron respectively. Top panels show footprint profiles (blue and red lines indicate bound and unbound TFs). Heatmaps display individual binding site signals of the TF footprints. (**C**, **D**) Changes in the top 5 TF binding sites for bound TFs between users and non-users in glia and neuron groups.

Thereafter, we sought to determine whether the observed differences in TF binding influence TF footprinting in non-users compared to heroin users. To achieve this, we employed the *TOBIAS Plot Heatmap* to correct the ATAC-seq signal within ± 60 base pair (bp) windows surrounding the occurrence of a given motif. Figure 2A,B and Supplemental Information Pages 1-10 present the differential footprint evidence in neuronal and glial cells, calculated based on flanking accessibility and foot-printing depth^39^. We identified patterns consistent with increased footprinting for both bound and unbound forms of TFs OTX2, ZNF135, NRF1, KLF15, and ZNF610 within the glial cell cohort of heroin users compared to non-users (Fig. 2A; Supplemental Information Page 1-3). Similarly, for the neuronal cell type, we observed higher inferred footprint scores for both bound and unbound TFs in heroin users compared to non-users. Specifically, the TFs JDP2, FOSJUNB, FOSL1JUNB, FOSBJUNB, ZBTB33, TCFL5, ZBTB14, NRF1, HINFP, ZFP57, ZNF610 and FOXN1 exhibited elevated footprint scores in heroin users (Fig. 2B; Supplemental Information Page 4-10). Figure 2C,D illustrate the differences in bound TF sites between the users and non-users of the neuronal and glial cohorts. Collectively, these findings suggest increased inferred footprinting in heroin users compared to non-users across both neuronal and glial cell types, the underlying causes of which remain to be elucidated through functional validation.

**Fig. 3:**
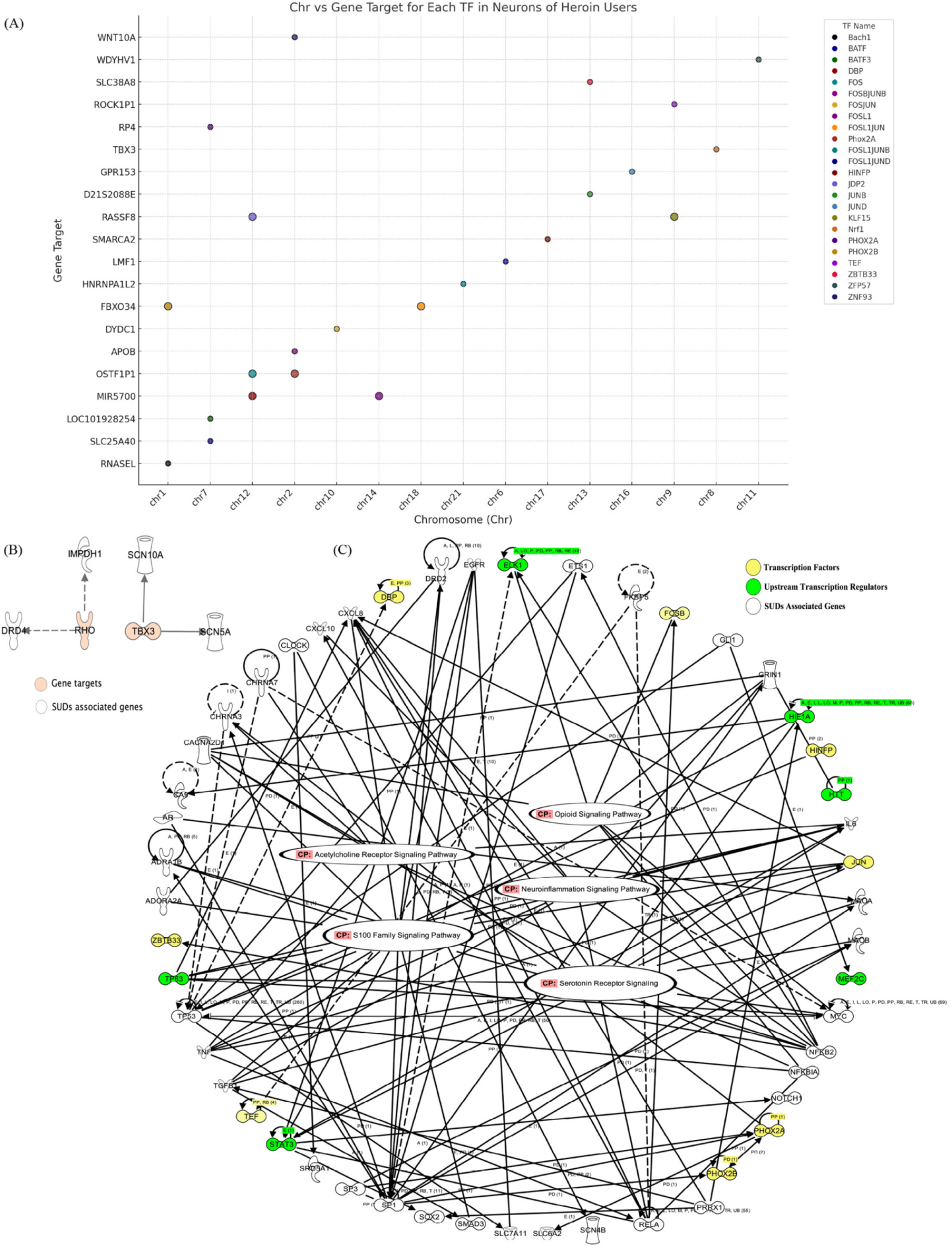
TF Networks and Interactions in Neurons of Heroin Users. (**A**) Chromosome vs. gene targets for TFs in neurons of heroin users, with each point representing a gene target mapped to its chromosome. Colors indicate TFs (legend), and bubble sizes represent gene target frequencies. (**B**) Regulatory relationships among DRD4, RHO, IMPDH1, TBX3, and SCN gene family members (SCN10A, SCN5A) in neurons, with solid and dashed lines for direct and indirect interactions, respectively. (**C**) Circular network (IPA analysis) showing interactions among TFs, upstream regulators, and SUD-associated molecules (genes). Solid lines represent direct interactions; dashed lines indicate expression relationship.

Following this, we annotated the unique bound regions of the selected TFs exclusive to users within both neuronal and glial cell types using computational methods (Supplementary Fig. 1) and proceeded to annotate these regions (Supplementary table 9 and 10). For the glial cell type, we found differentially bound TFs of this cohort are inferred to occupy promoters of *PRDM9* and *USP17L* family of genes (Supplementary table 10). We identified via *IPA* that *USP17L27* (which includes all *USP17L* family of TFs) interacts with *CSF1, STAT3, and MYC* genes which in turn regulate various genes involved in downstream signaling for e.g. serotonin receptor signaling (Supplementary Fig. 3A). For the neuron cohort, we found the differential TFs to be targeting promoters or enhancers regions of *RNASEL, MIR5700, OSTF1P1, LOC101928254, SLC25A40, FBXO34, ROCK1P1, DYDC1, LINC01068, B3GNT5, and TBX3* genes (Fig. 3A. We noted, in neurons of heroin users, TFs were predominantly bound at Chr12 at both enhancer and promoter regions targeting genes *OSTF1P1, RP4, TBX3, MIR5700, RASSF8* (Supplementary table 9). Performing pathway analysis using *IPA* on these target genes, we found *RHO (RP4),* one of the target genes of the selected TFs in the neuron cohort interacted with *DRD4* and *IMPHD1*. Another gene of the same cohort, *TBX3*, interacted with *SCN10A* and *SCN5A* (Fig. 3B).

### Upstream regulator analysis reveals interaction with SUD-associated genes

Upstream regulator analysis was performed on the TFs identified in glial and neuronal cell types (Fig. 1G). Results presented in Fig. 3C and Supplementary Fig. 3B illustrate the interactions between the identified differentially bound TFs in this study, their upstream transcriptional regulators and SUD-associated genes (using *IPA*). In neuronal cell types, differentially bound TFs such as DBP, FOSB, HINFP, JUN, PHOX2A/B, and TEF were found to interact with their respective transcriptional regulators and SUDs-related genes (Fig. 3C). Notably, pathways including acetylcholine, serotonin, opioid, neuroinflammation, and S100 family signaling were activated. Similarly, in glial cell types, differentially bound TFs, including ELK3, ETV5, KLF15, MGA, NRF1, OTX2, and ZNF610, were observed to interact with corresponding transcriptional regulators and SUDs-related genes (Supplementary Fig. 3B). These interactions were associated with the activation of pathways such as oxytocin, synaptogenesis, opioid, neuroinflammation, and CREB signaling. Gene ontology (GO) analysis suggested both significant and non-significant enrichment in cell maintenance, metabolism, and signaling pathways within the user and non-user populations of both neuronal and glial cells (Supplementary Fig. 3C–F).

### TF co-occurrence analysis

**Fig. 4:**
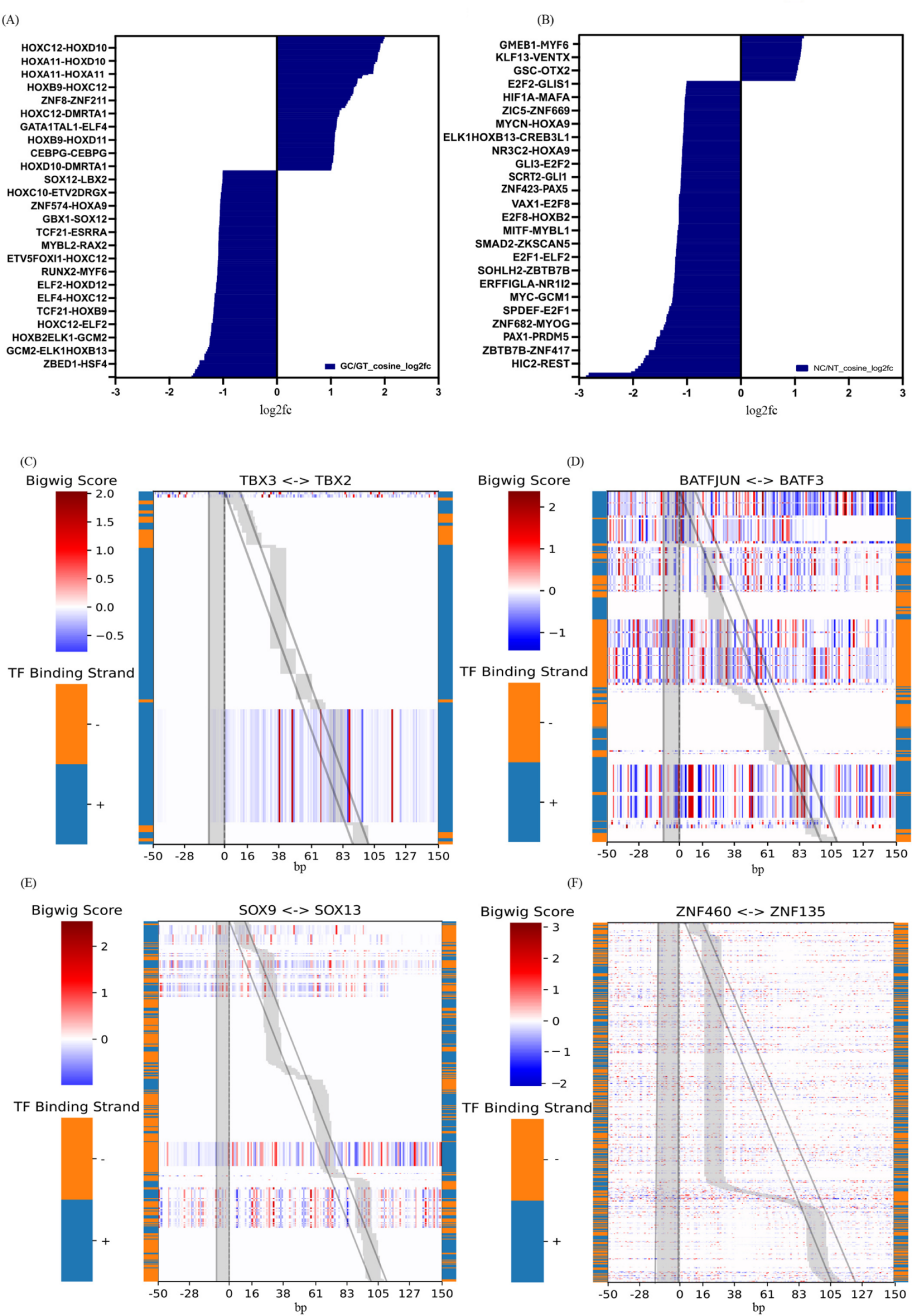
Differential TF Co-Occurrence and Binding Patterns in Glia and Neurons. (**A**, **B**) Differential cosine similarity scores for top 25 highest and lowest co-occurring TF pairs; y-axis shows TF pairs, x-axis indicates log₂FC. (**C**–**F**) PairMaps of unique co-occurring TF pairs in non-user glia (**C**, **D**) and user neurons (**E**, **F**); bigwig scores represent binding; y-axis denotes TF binding strand (orange: negative, blue: positive). The x-axis shows the genomic positions (bp) from -50 to 150 bp around binding sites.

Subsequently, we sought to predict TFs that, in conjunction with those in our dataset, orchestrate regulatory functions and to elucidate how the binding grammar of these TFs influences downstream pathways. To achieve this, we employed the *TFBS from motifs* feature of TF-COMB to detect co-occurring TFs within each cohort. *Market basket analysis* was utilized to determine the likelihood of two TFs co-occurring at specific regulatory regions. We ensured that each co-occurrence was counted only once per window by applying the *binarize* = *True* filter and the *count_within* parameter in the TF-COMB module. Our analysis initially identified 618,790, 615,947, 616,913, and 621,821 TF pairs for neurons from heroin users (NT), neurons from non-users (NC), glia from heroin users (GT), and glia from non-users (GC), respectively (Supplementary Fig. 4A). Following the application of filters based on cosine similarity (ranging from + 1 to −1) and positive z-scores (as negative Z scores depicted anti co-occurrence)^40^, we identified a total of 7,025, 6,745, 6,177, and 6,210 TF pair co-occurrences for the NT, NC, GT, and GC, respectively (Supplementary Fig. 4B). We then performed a differential cosine analysis based on log₂fc cosine values comparing users and non-users in both neuronal and glial cells (Fig. 4A, B). The figure illustrates the top 25 highest and lowest differential log₂ cosine values of co-occurring TF pairs. Thereafter we put a significant cosine threshold of > 0.5 and analyzed the unique and common TF co-occurrence pairs in heroin users versus non-users within each cell cohort. In the glial cohort, we identified 13 unique co-occurring pairs for non-users and none for users that were significant (cosine > 0.5) (Supplementary Fig. 5A; Supplementary table 11). Further downstream analysis of 6 of the co-occurring TFs identified indicated that most TF-gene interactions were associated with neuroinflammation, serotonin receptor signaling, and S100 family signaling pathways (Supplementary Fig. 5B). Contrary to this, in the neuronal cell type (Supplementary Fig. 6A; Supplementary table 12), we identified 5 unique co-occurrence pairs for users and no co-occurrence unique pair for non-users that co-occurred significantly (cosine > 0.5). Downstream analysis (Supplementary Fig. 6B) of seven of these unique TF co-occurrences in the neurons of users suggested the upregulation of neuroinflammatory genes and indicated the involvement of neuroinflammation, serotonin receptor signaling, S100 family signaling pathways and oxytocin in brain signaling pathway which are associated with various SUDs related disorders.

Next, we investigated common TF pairs where both TFs were associated with SUDs related gene database (based on *IPA*) and applied filters of cosine > 0.5. This analysis revealed 15 TF pairs for the glial cohort and 12 TF pairs for the neuronal cell type (Supplementary table 13 and 14). Among these, ten TF pairs DLX3-DLX4, EMX1-LHX8, EMX1-TLX2, HOXA2-VAX1, LHX8-TLX2, NKX6-2-PRRX1, NKX6-2-PRRX2, NKX6-2-SHOX2, PRRX1-SHOX2, and TBX1-TBX4 were common across both cell types. Although the common co-occurring TF pairs in users versus non-users within neuronal and glial cells were identified significant, the differential cosine score were not found to be significant.

### Footprinting patterns of unique and common co-occurring TF pairs

We created PairMaps to visualize the footprinting pattern of the unique and common TF pairs found in our analysis (Fig. 4C-F, Supplementary Fig. 7A-–D, and Supplemental Information Pages 11-79). Binding TFs can create small regions of inaccessible DNA at their binding sites, referred to as footprints. The PairMaps are designed to illustrate this phenomenon for specific TF pairs, showing how the footprint evolves with increasing distances between the two TFs. The bigwig score reflects DNA accessibility at a specific position, where values greater than 0 indicate accessibility and values less than 0 suggest blockage. In the PairMaps, each row represents a genomic location containing the TF pair. Patterns at marked locations indicate the presence of footprints created by the TFs. Additionally, the PairMaps reveal whether the TF pair exhibits a preferred binding distance by displaying the frequency of specific distances (bp). The length of red or blue lines in each row depicts the respective size of the genomic locations^40,42^. Figure 4C,D shows PairMaps for TF pairs TBX3-TBX2 and BATFJUN-BATF3 which were found to be unique pairs in glia of non-users. Figure 4E,F show PairMaps for SOX9-SOX13 and ZNF460-ZNF135 which were found in neurons of heroin users. We identified differences in binding and a gradual divergence among the unique TF pairs in glial and neuronal cells. Similarly, Supplementary Fig. 5(C-F) show PairMaps for common TF pairs VAX1-HOXA2 in neurons of non-users, neurons of users, glia of non-users, and glia of users respectively. We identified the differences in binding sites and preferred binding distances of the common TF pair VAX1-HOXA2 in users and non-users of neuron and glial cells. Additional PairMaps for unique and common TF pairs are provided in the Supplemental Information pages 11-79. Together, these visualizations highlight the footprinting pattern differences of the predicted TFs in our study.

## Discussion

We carried out an in-depth analysis of TF binding, footprinting patterns, and co-occurrence analysis in neuronal and glial cells derived from heroin users and non-users. Altered chromatin accessibility patterns in neurons and glial cells are closely associated with gene expression dysregulation, shaping the developmental and ongoing processes that underpin substance addiction. Neurons primarily contribute through altered neurotransmission and synaptic plasticity, while glial cells play a critical role in orchestrating key processes including neuronal generation, migration, axon differentiation and elongation, modulate inflammatory response and guiding circuit formation and synapse development^6,43–45^. Hence, a comprehensive understanding of these changes is essential for developing effective treatments for addiction. Our findings suggest distinct inferred TF binding and footprinting profiles (Figs. 1 and 2), with patterns consistent with upregulation in neuroinflammatory pathways associated with addiction (Fig. 3B, Supplementary Fig. 3B). Furthermore, we identified both unique and shared TF co-occurrence patterns, highlighting differential binding behaviors between heroin users and non-users (Supplementary Tables 11–14).

While both glial and neuronal cells showed elevated levels of TF binding in heroin users against non-users, a large number of TFs and their families showed diminished levels of binding scores (log_2_FC) in the neuronal cells of the user cohort. Using the 99th and 1st percentile cutoffs (and p value < 0.05), we determined the positive and negative binding TFs in the glial and neuronal cell types. Our analysis of footprint differences between users and non-users in both neurons and glia suggested an increase in inferred footprinting among users in both cell types (Fig. 2A, B and Supplemental Information Pages 1–10). Consistent with our findings, previous studies show upregulation of transcriptional regulators like CREB, ΔFosB, NFκB, and others impacting downstream regulation and addiction-related behavior and neuroplasticity in OUD^46–48^ and other SUDs. While TFs like ΔFosB, CREB, and NFκB^49–51^ showed alterations in neurons, others, including STAT3 and NFκB, exhibited changes in glial cells, indicating cell-specific TF regulation^52,53^. Studies in intact male rats demonstrated that μ opioid agonists acutely enhance c-fos and JunB expression in various brain regions, while embryonic ethanol exposure was found to decrease Otx2 mRNA levels in the adult mouse central nervous system^54,55^. We extracted unique genomic coordinates (Supplementary Table 6, 7) for differentially bound TFs, observing a staggering reduction in number of distinct TF binding regions in heroin users compared to non-users in both glial and neuronal cells. Most of these unique regulatory regions of the TFs were classified as bound to promoters, with the remainder categorized as bound to enhancers (Supplementary Table 9, 10) across both cell types and groups (Fig. 1H, Supplementary Fig. 2C). We suggest that the reduced binding sites observed in users could result from factors like epigenetic changes, chromatin remodeling, non-epigenetic chromatin inaccessibility, RNA interference, protein–protein interactions, mutations, or substance-use associated inhibition of TF-DNA binding^56–58^. This hypothesis aligns with previous studies demonstrating changes in histone acetylation, methylation, and other epigenetic alterations in the brain’s reward system following chronic exposure to opioids and other substances^56,59^. We found two differentially bound TFs, NRF1 and KLF15 to be common in both neuron and glia cell types. Changes in gene expression of NRF1 were reported with fentanyl exposure in mice^60^, while KLF15 was found to be associated with oxycodone exposure and related addictive behavior in rodents^61^. Moreover, elevated inferred binding of NRF1 and KLF15 raises the possibility that these factors may be associated with adaptive responses to heroin exposure; however, without gene expression (RNA-seq or qPCR data), or protein-level validation, their functional role cannot be established from the present data alone^62,63^.

Upstream regulator analysis (Fig. 3B, Supplementary Fig. 3B) was conducted on the differentially bound TFs previously identified within the glial and neuronal cell types (Fig. 1G). This analysis revealed that the selected TFs and their upstream transcriptional regulators interact with genes associated with SUDs. Utilizing *IPA,* we observed that in the glial cohort (Supplementary Fig. 3B), the TF OTX2 regulates *GNRH1*, which is implicated in substance abuse-related brain circuitry^64^. Additionally, *GBX2*, linked to AUD, is also regulated by *OTX2*. Both *GNRH1* and *GBX2* are associated with METH addiction [3]. The cannabinoid receptor gene *CNR1*, which is associated with substance dependence and may serve as a predictor for SUDs, is regulated by the TF *CIC*^65,66^. Furthermore, *CCND1* and *GRIK3*, both genes associated with AUD, are also regulated by *CIC*^67,68^. *GABARAPL1*, which plays critical roles in METH dependence treatment^69^, interacts with *LMX1B*, which in turn, is regulated by *NRXN1*, a gene linked to nicotine dependence^70^. *HTT* also regulates *DRD2*, which is involved in SUDs including cocaine, nicotine and opioid dependence^71,72^. *SERTAD2* regulates *ADRB3*, an addiction signaling gene^73^. *POU4F1* regulates the genes *ADCYAP1*, *CARTPT, GBX2* and *CHRNA3*. Variations in these genes contribute to various forms of drug dependence. *ATXN1* interacts with *GRM1* and *CHRNA7*, both of which are associated with addictive behaviors^74–76^. In the neuronal cell type (Fig. 3B), we identified that *FKBP5*, a gene associated with cocaine and morphine dependence, regulates TEF, a differentially expressed TF identified in this study^77^. ZBTB33, which is regulated by *NFKB2*, is also implicated in behaviors associated with the addiction cycle. *TP53*, a gene associated with cross-generational AUD, regulates DBP, which is also regulated by HINFP, latter two being differentially bound in our study. Additionally, *CHRNA7* regulates *MYC*, another gene associated with AUD and is regulated by *TP53*^78–80^. *SP1* together with PHOX2A, a differentially bound TF of this study, regulates *DRD2*^81^. *SP3* regulates *MAOB*, polymorphisms of which are associated with alcohol and opiate addiction^77^. Similarly, *CA9*, which is regulated by HIF1A, is also implicated in different forms of addiction^81^. *CLOCK* regulates *SCN4B* and *SRD5A1*, both of which are significantly correlated with lifetime alcohol abuse^82^ and OUD therapy, respectively^82^. Collectively, these findings suggest an association between differentially inferred TF binding and genes previously implicated in SUDs, though causal roles remain to be established. Moreover, differences in footprinting between non-user and user groups in both neuronal and glial cells may lead to alterations in SUDs-associated downstream pathways, potentially contributing to vulnerability to addiction and addictive behavior, a hypothesis that requires functional validation. Together, these revealed genes involved in neuroinflammation, synaptogenesis, and signaling pathways associated with CREB, opioids, and neurotransmitters in neurons and glia (Fig. 3B, Supplementary Fig. 3B) which is consistent with findings from previous addiction studies^50,83–85^. Furthermore, we identified associations between AUD and downstream gene targets including GBX2, CCND1, GRIK3, ALDH1A1, MYC, and TP53, as shown in Fig. 3B and Supplementary Figs. 3A and 3B. These relationships may indicate the presence of multiple addictions among substance users and suggest that common neural signaling pathways are activated during the use of various substances^86,87^. We identified activation of the neuroinflammatory and S100 signaling pathway (Fig. 3B, Supplementary Figs. 3B, 5B and 6B). Previous studies have shown that S100B and inflammatory cytokine levels in the blood may serve as potential markers for blood-brain barrier damage and psychiatric impairment in individuals with co-morbid hepatitis C viral infection and alcohol use disorder^88^. Notably, the involvement of the S100 pathway in addiction has not been reported previously, highlighting the need for further investigation. Differential cosine analysis, based on log₂fc values between heroin users and non-users, identified the top 25 (highest and lowest) co-occurring TF pairs exhibiting significant differential co-occurrence patterns (Fig. 4A, B). TF co-occurrence prediction analysis identified TFs with distinct predicted co-occurrence patterns in users versus non-users in both neurons and glia which can lay the ground for future TF-TF interaction studies. In the glial cohort, we identified 13 unique significant co-occurrence pairs (cosine > 0.5) in non-users, with none in heroin users (Supplementary Table 11). Downstream analysis suggested that these TF pairs in non-users are also associated with gene interactions involved in activation of neuroinflammation, serotonin receptor signaling, and S100 family signaling (Supplementary Fig. 5A and 5B). Conversely, In the neuronal cell type, we identified five unique, significantly co-occurring TF pairs (cosine > 0.5) in heroin users, while no unique pairs were observed in non-users (Supplementary Table 12). Further analysis of these unique TF co-occurrences in heroin users’ neurons suggested an upregulation of pathways related to neuroinflammation, oxytocin, serotonin receptor, and S100 family signaling (Supplementary Fig. 6A and 6B). Although functional evidence is needed for future studies, the absence of the aforementioned TF pairs in heroin users in glial cohort suggests that heroin use may downregulate genes essential for glial cell function. This downregulation could impair glial roles in supporting neurons, regulating neurotransmitter levels, and maintaining brain homeostasis^89–91^. Many of these TFs are involved in development and differentiation; thus, their reduced expression might lead to altered glial maturation and function, potentially affecting neural circuits involved in addiction^92–95^. Furthermore, the inferred accessibility of these TFs in neurons of heroin users, if functionally confirmed, may lead to changes in gene expression that promote neuronal plasticity associated with addiction^96,97^. Common TF pairs (Supplementary Fig. 7A-7D, Supplementary Table 13–14) identified across both neuronal and glial cells, despite lacking significant differential co-occurrence between users and non-users, may represent fundamental cellular regulatory mechanisms that remain resilient to heroin exposure. These observations suggest that neurons and glia engage distinct transcriptional control networks, each influencing addiction-related adaptations through separate functional routes though functional validation is needed. To summarize, these findings deepen our understanding of cell-type–specific TF regulation and co-occurrence patterns in the context of heroin addiction, providing crucial insights for future research and therapeutic interventions^18,98,99^.

Our bioinformatics re-analysis of publicly available ATAC-seq data (PRJNA561094, Egervari et al., 2020) identifies chromatin accessibility patterns associated with heroin use in neuronal and glial nuclei of the putamen. There are several limitations to this study. This work is intentionally hypothesis-generating rather than direct evidence of heroin-driven regulatory remodeling. ATAC-seq footprinting infers TF occupancy computationally and does not directly measure TF binding or activity; without matched transcriptomic or proteomic data, differential activity of the identified TFs at the RNA or protein level cannot be confirmed. Given the absence of functional validation, all mechanistic interpretations should be treated as preliminary. The identified TF candidates, particularly those relevant to striatal circuit, represent interesting leads for future investigation. Individual-level TF binding scores were not computed, as the TOBIAS BinDetect module prefers merged group-level BAM files to achieve sufficient read depth for reliable footprint detection at TF motif sites. Additional limitations may include limited post-mortem tissue availability, incomplete metadata, and gender imbalance in the studied cohort.

## Supplementary Information

Below is the link to the electronic supplementary material.

Supplementary Material 1

Supplementary Material 2

Supplementary Material 3

Supplementary Material 3

## Data Availability

The raw data for this study were obtained from the NCBI Sequence Read Archive under accession PRJNA561094, as originally reported by Egervari et al. 2020 (Nat. Commun. 11, 4634; 2020).
