# Supplementary material for: Heroin addiction modulates transcription factor binding in regulatory regions of the human putamen
Source: Sci Rep. 2026 May 12;16:21737. doi: 10.1038/s41598-026-52754-7 (PMC13357565; doi:10.1038/s41598-026-52754-7)
Supplement: Supplementary file 2 — Supplementary Material 2 [file 41598_2026_52754_MOESM2_ESM.pdf]

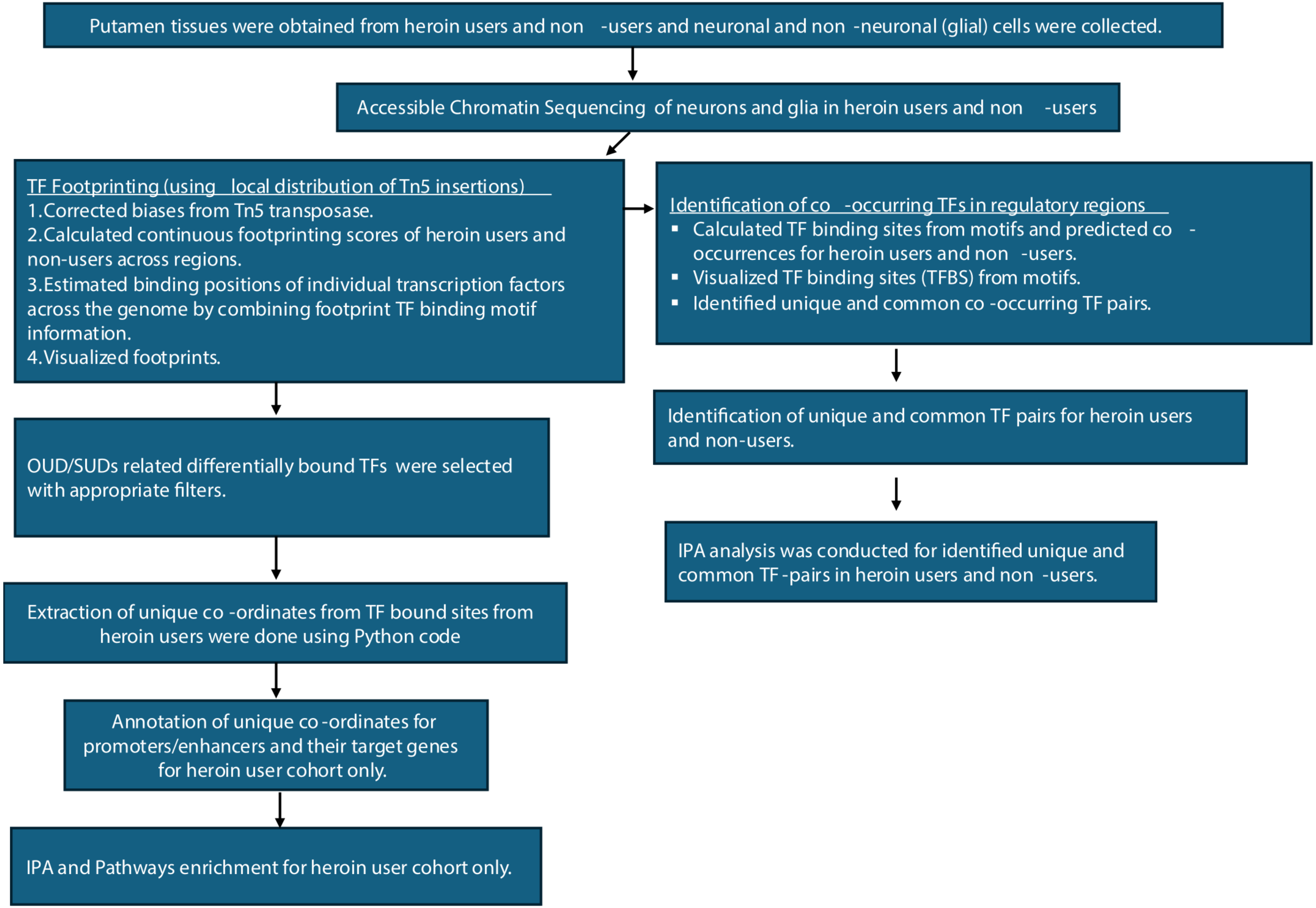
Putamen tissues were obtained from heroin users and non -users and neuronal and non -neuronal (glial) cells were collected.
Accessible Chromatin Sequencing of neurons and glia in heroin users and non -users
TF Footprinting (using local distribution of Tn5 insertions)
1.Corrected biases from Tn5 transposase.
2.Calculated continuous footprinting scores of heroin users and non-users across regions.
3.Estimated binding positions of individual transcription factors across the genome by combining footprint TF binding motif information.
4.Visualized footprints.
Identification of co -occurring TFs in regulatory regions
Calculated TF binding sites from motifs and predicted co - occurrences for heroin users and non -users.
Visualized TF binding sites (TFBS) from motifs.
Identified unique and common co -occurring TF pairs.
OUD/SUDs related differentially bound TFs were selected with appropriate filters.
Identification of unique and common TF pairs for heroin users and non-users.
IPA analysis was conducted for identified unique and common TF -pairs in heroin users and non -users.
Extraction of unique co -ordinates from TF bound sites from heroin users were done using Python code
Annotation of unique co -ordinates for promoters/enhancers and their target genes for heroin user cohort only.
IPA and Pathways enrichment for heroin user cohort only.

(A)

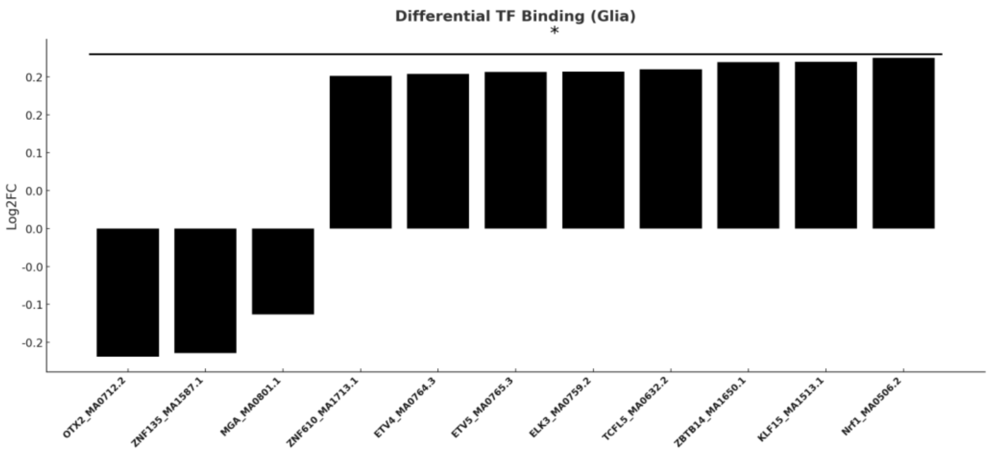
Differential TF Binding (Glia)
*
Log2FC
0.2
0.2
0.1
0.0
0.0
-0.0
-0.1
-0.2
OTX2_MA0712.2
ZNF135_MA1587.1
MGA_MA0801.1
ZNF610_MA1713.1
ETV4_MA0764.3
ETV5_MA0765.3
ELK3_MA0759.2
TCFL5_MA0632.2
ZBTB14_MA1650.1
KLF15_MA1513.1
Nrf1_MA0506.2

(B)

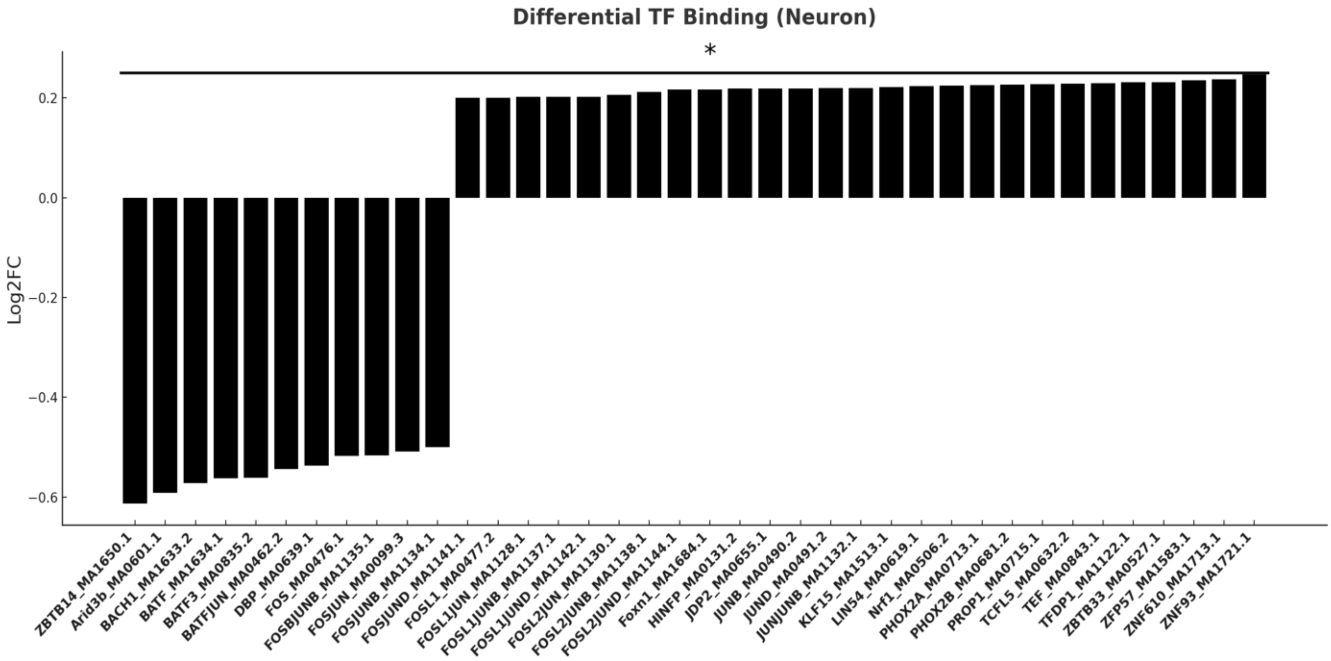
Differential TF Binding (Neuron)
*
Log2FC
0.2
0.0
−0.2
−0.4
−0.6
ZBTB14_MA1650.1
Arid3b_MA0601.1
BACH1_MA1633.2
BATF_MA1634.1
BATF3_MA0835.2
BATFJUN_MA0462.2
DBP_MA0639.1
FOS_MA0476.1
FOSBJUNB_MA1135.1
FOSJUN_MA0099.3
FOSJUNB_MA1134.1
FOSJUND_MA1141.1
FOSL1_MA0477.2
FOSL1JUN_MA1128.1
FOSL1JUNB_MA1137.1
FOSL1JUND_MA1142.1
FOSL2JUN_MA1130.1
FOSL2JUNB_MA1138.1
FOSL2JUND_MA1144.1
Foxn1_MA1684.1
HINFP_MA0131.2
JDP2_MA0655.1
JUNB_MA0490.2
JUND_MA0491.2
JUNJUNB_MA1132.1
KLF15_MA1513.1
LIN54_MA0619.1
Nrf1_MA0506.2
PHOX2A_MA0713.1
PHOX2B_MA0681.2
PROP1_MA0715.1
TCFL5_MA0632.2
TEF_MA0843.1
TFDP1_MA1122.1
ZBTB33_MA0527.1
ZFP57_MA1583.1
ZNF610_MA1713.1
ZNF93_MA1721.1

(C)

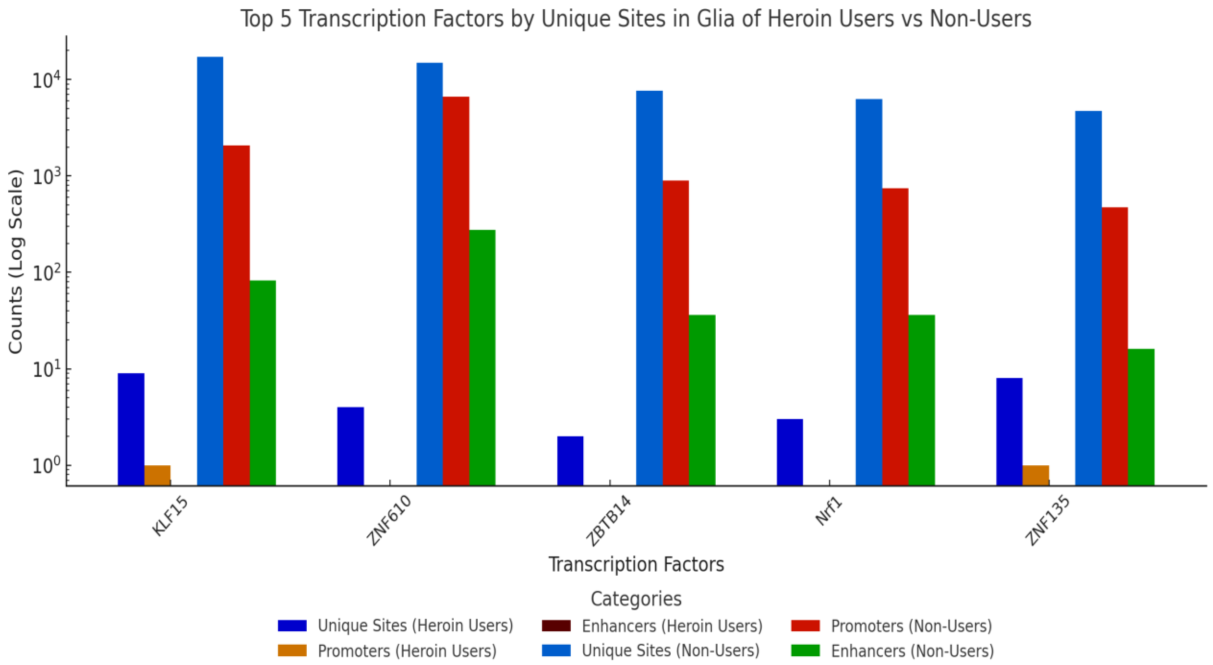
Top 5 Transcription Factors by Unique Sites in Glia of Heroin Users vs Non-Users
Counts (Log Scale)
10^4
10^3
10^2
10^1
10^0
KLF15
ZNF610
ZBTB14
Nrf1
ZNF135
Transcription Factors
Categories
Unique Sites (Heroin Users)
Promoters (Heroin Users)
Enhancers (Heroin Users)
Unique Sites (Non-Users)
Promoters (Non-Users)
Enhancers (Non-Users)

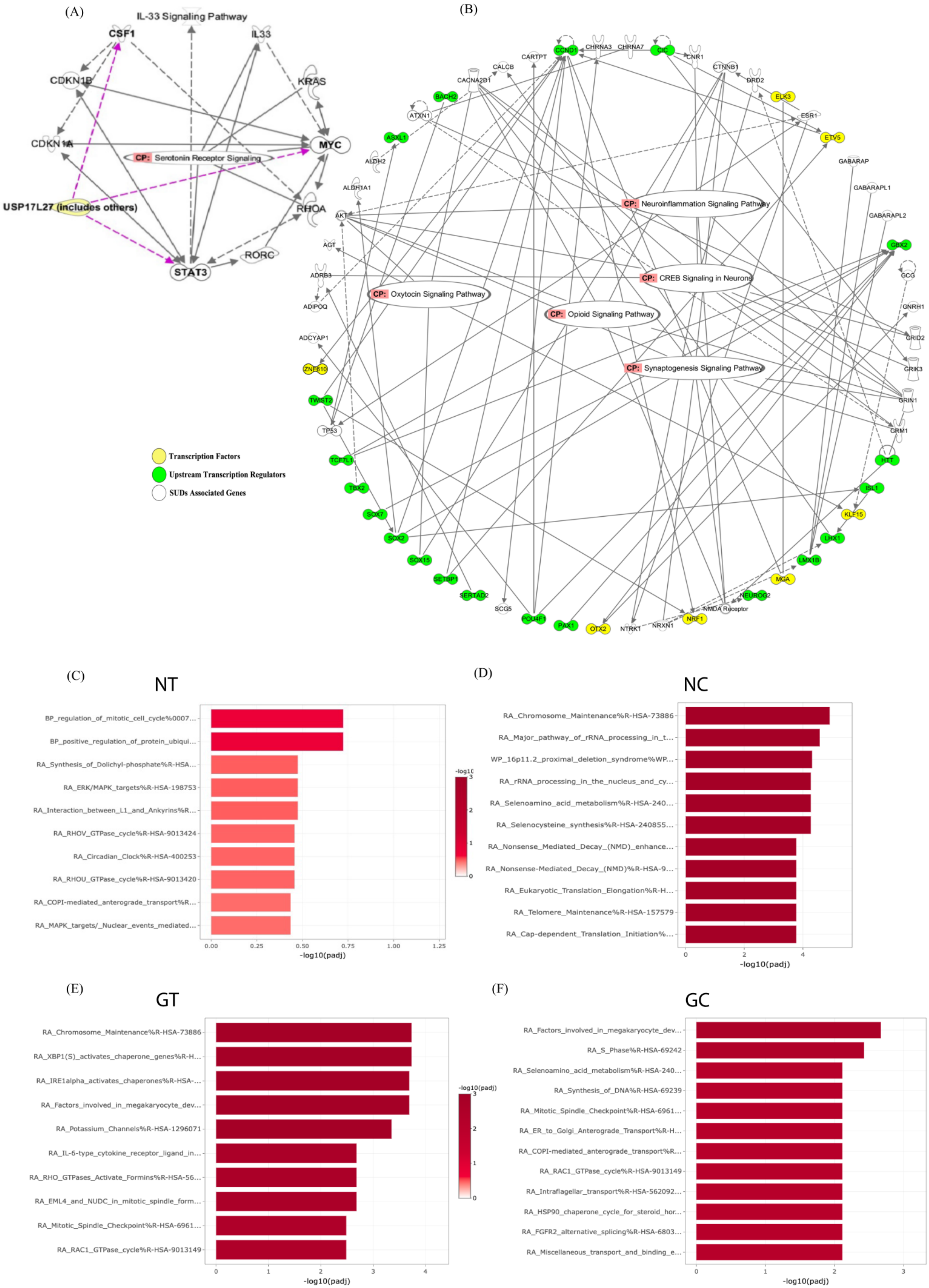

(A)
IL-33 Signaling Pathway
CSF1
IL33
CDKN1B
KRAS
CDKN1A
MYC
CP: Serotonin Receptor Signaling
USP17L27 (includes others)
RHOA
RORC
STAT3
Transcription Factors
Upstream Transcription Regulators
SUDs Associated Genes
(B)
CP: Neuroinflammation Signaling Pathway
CP: CREB Signaling in Neurons
CP: Oxytocin Signaling Pathway
CP: Opioid Signaling Pathway
CP: Synaptogenesis Signaling Pathway
CCND1
CHRNA3
CHRNA7
CIC
CNR1
CARTPT
CALCB
CTNNB1
DRD2
CACNA2D1
BACH2
ELK3
ATXN1
ESR1
ASXL1
ETV5
ALDH2
GABARAP
ALDH1A1
GABARAPL1
AKT
GABARAPL2
AGT
GBX2
ADRB3
GCG
ADIPOQ
GNRH1
ADCYAP1
GRID2
ZNF610
GRIK3
TWIST2
GRIN1
TP53
GRM1
TCF7L1
HTT
TBX2
ISL1
SOX7
KLF15
SOX2
LHX1
SOX15
LMX1B
SETBP1
MGA
SERTAD2
NEUROG2
SCG5
NMDA Receptor
POU4F1
PAX1
OTX2
NTRK1
NRXN1
NRF1
(C)
NT
BP_regulation_of_mitotic_cell_cycle%0007...
BP_positive_regulation_of_protein_ubiqui...
RA_Synthesis_of_Dolichyl-phosphate%R-HSA...
RA_ERK/MAPK_targets%R-HSA-198753
RA_Interaction_between_L1_and_Ankyrins%R...
RA_RHOV_GTPase_cycle%R-HSA-9013424
RA_Circadian_Clock%R-HSA-400253
RA_RHOU_GTPase_cycle%R-HSA-9013420
RA_COPI-mediated_anterograde_transport%R...
RA_MAPK_targets/_Nuclear_events_mediated...
0.00
0.25
0.50
0.75
1.00
1.25
-log10(padj)
(D)
NC
RA_Chromosome_Maintenance%R-HSA-73886
RA_Major_pathway_of_rRNA_processing_in_t...
WP_16p11.2_proximal_deletion_syndrome%WP...
RA_rRNA_processing_in_the_nucleus_and_cy...
RA_Selenoamino_acid_metabolism%R-HSA-240...
RA_Selenocysteine_synthesis%R-HSA-240855...
RA_Nonsense_Mediated_Decay_(NMD)_enhance...
RA_Nonsense-Mediated_Decay_(NMD)%R-HSA-9...
RA_Eukaryotic_Translation_Elongation%R-H...
RA_Telomere_Maintenance%R-HSA-157579
RA_Cap-dependent_Translation_Initiation%...
0
2
4
-log10(padj)
(E)
GT
RA_Chromosome_Maintenance%R-HSA-73886
RA_XBP1(S)_activates_chaperone_genes%R-H...
RA_IRE1alpha_activates_chaperones%R-HSA-...
RA_Factors_involved_in_megakaryocyte_dev...
RA_Potassium_Channels%R-HSA-1296071
RA_IL-6-type_cytokine_receptor_ligand_in...
RA_RHO_GTPases_Activate_Formins%R-HSA-56...
RA_EML4_and_NUDC_in_mitotic_spindle_form...
RA_Mitotic_Spindle_Checkpoint%R-HSA-6961...
RA_RAC1_GTPase_cycle%R-HSA-9013149
0
1
2
3
4
-log10(padj)
-log10(padj)
(F)
GC
RA_Factors_involved_in_megakaryocyte_dev...
RA_S_Phase%R-HSA-69242
RA_Selenoamino_acid_metabolism%R-HSA-240...
RA_Synthesis_of_DNA%R-HSA-69239
RA_Mitotic_Spindle_Checkpoint%R-HSA-6961...
RA_ER_to_Golgi_Anterograde_Transport%R-H...
RA_COPI-mediated_anterograde_transport%R...
RA_RAC1_GTPase_cycle%R-HSA-9013149
RA_Intraflagellar_transport%R-HSA-562092...
RA_HSP90_chaperone_cycle_for_steroid_hor...
RA_FGFR2_alternative_splicing%R-HSA-6803...
RA_Miscellaneous_transport_and_binding_e...
0
1
2
3
-log10(padj)

(A)

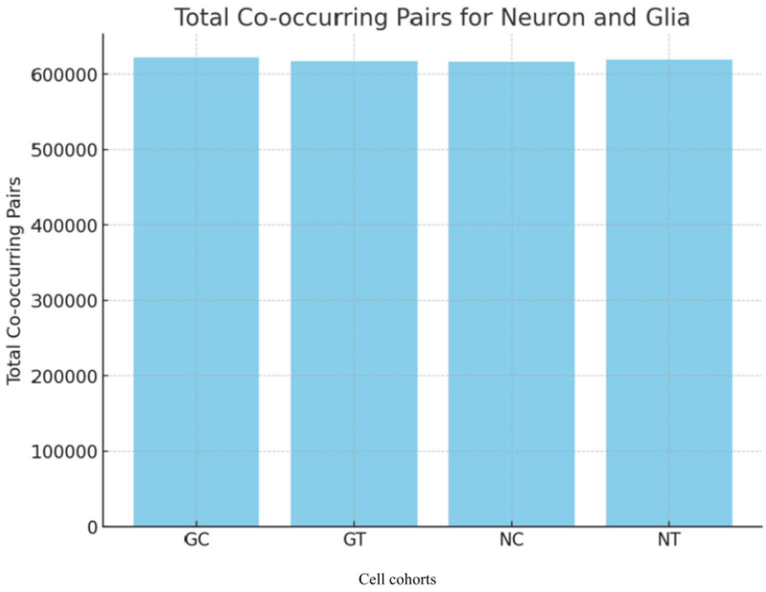
Total Co-occurring Pairs for Neuron and Glia
Total Co-occurring Pairs
600000
500000
400000
300000
200000
100000
0
GC
GT
NC
NT
Cell cohorts

(B)

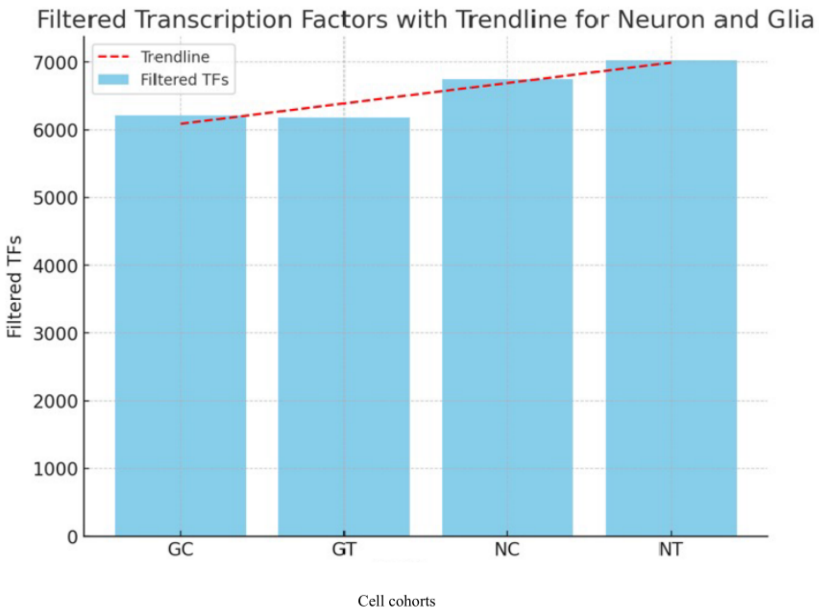
Filtered Transcription Factors with Trendline for Neuron and Glia
Trendline
Filtered TFs
Filtered TFs
7000
6000
5000
4000
3000
2000
1000
0
GC
GT
NC
NT
Cell cohorts

(A)

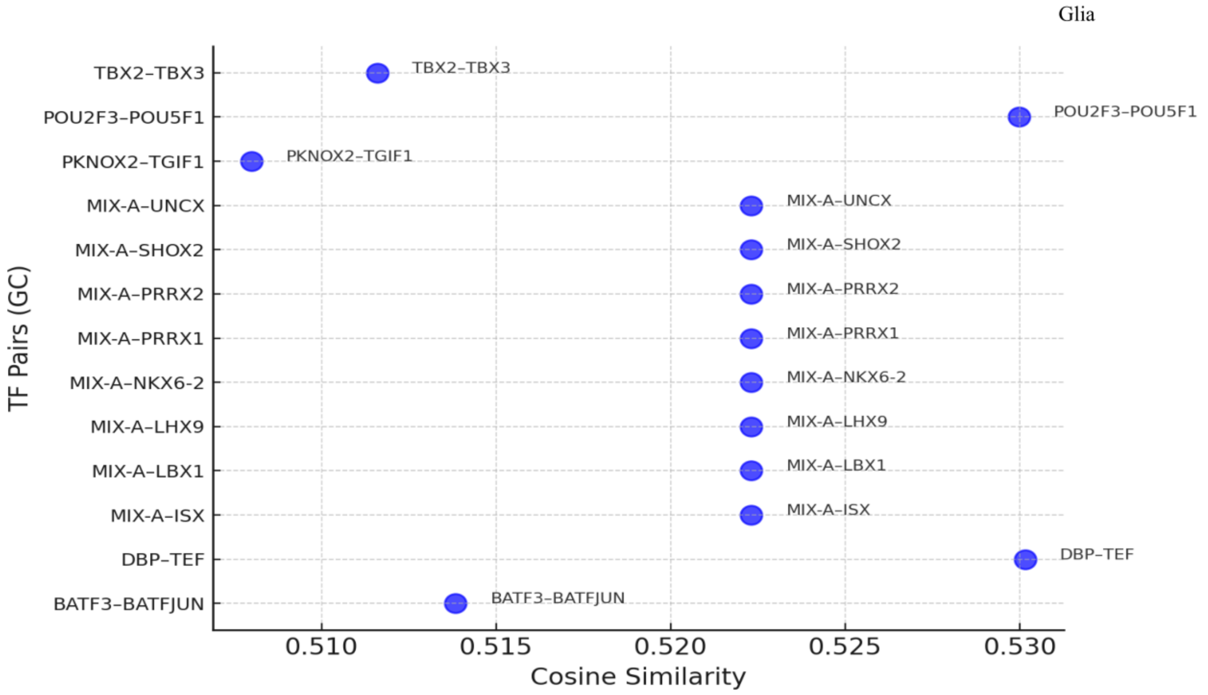
Glia
TF Pairs (GC)
TBX2–TBX3
POU2F3–POU5F1
PKNOX2–TGIF1
MIX-A–UNCX
MIX-A–SHOX2
MIX-A–PRRX2
MIX-A–PRRX1
MIX-A–NKX6-2
MIX-A–LHX9
MIX-A–LBX1
MIX-A–ISX
DBP–TEF
BATF3–BATFJUN
0.510
0.515
0.520
0.525
0.530
Cosine Similarity

(B)

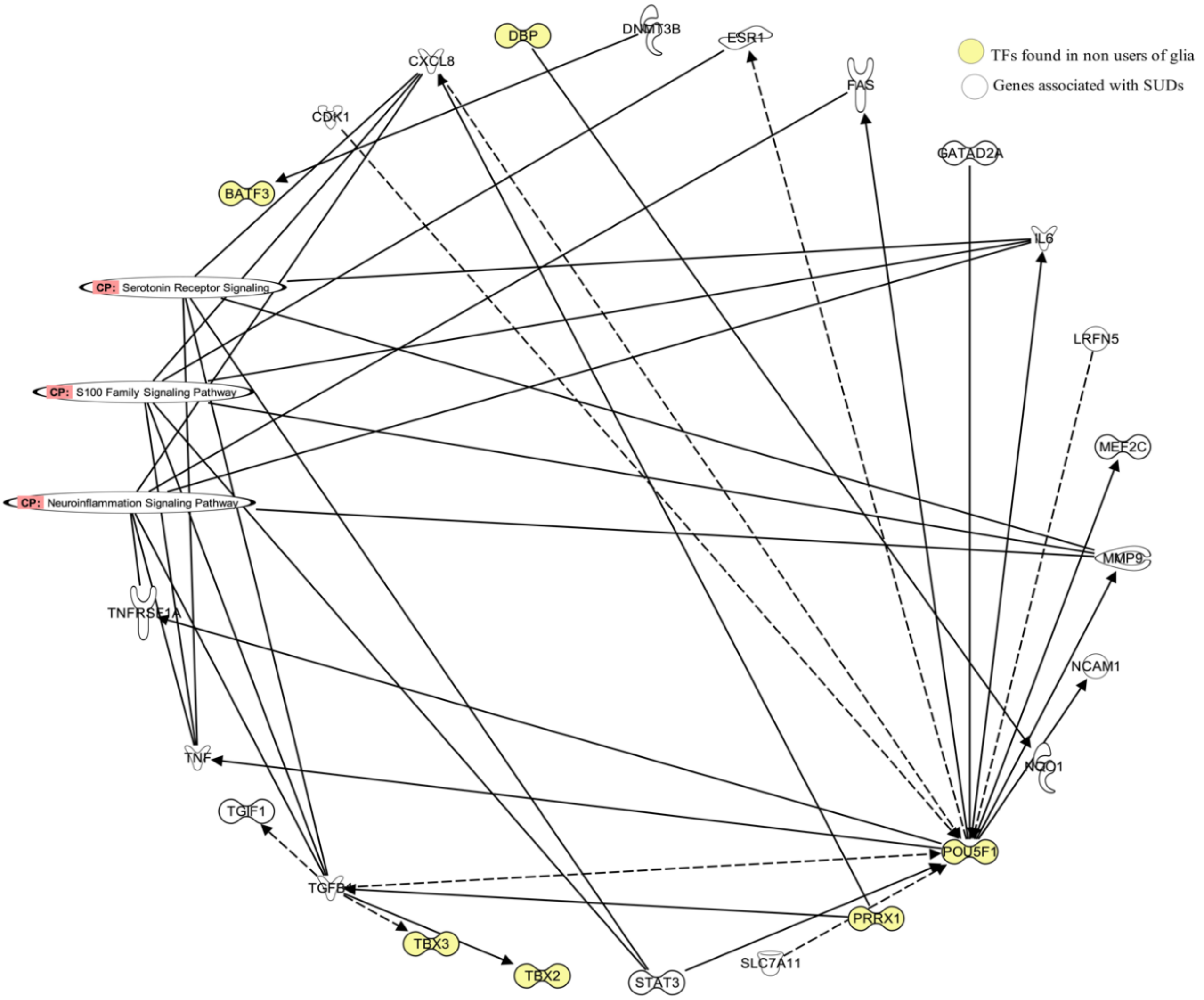
TFs found in non users of glia
Genes associated with SUDs
DBP
DNMT3B
ESR1
CXCL8
FAS
CDK1
GATAD2A
BATF3
IL6
CP: Serotonin Receptor Signaling
LRFN5
CP: S100 Family Signaling Pathway
MEF2C
CP: Neuroinflammation Signaling Pathway
MMP9
TNFRSF1A
NCAM1
TNF
NQO1
TGIF1
POU5F1
TGFB1
PRRX1
TBX3
SLC7A11
TBX2
STAT3

(A)

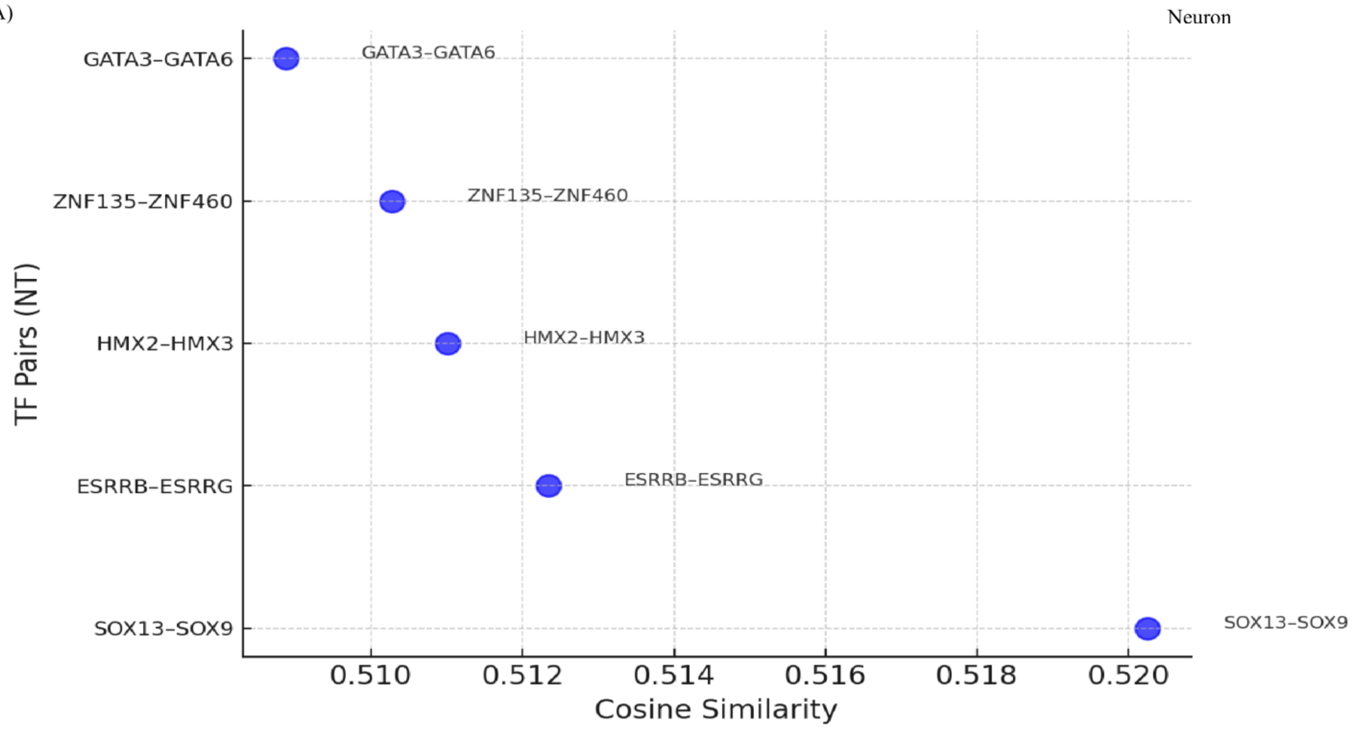
Neuron
TF Pairs (NT)
GATA3–GATA6
ZNF135–ZNF460
HMX2–HMX3
ESRRB–ESRRG
SOX13–SOX9
0.510
0.512
0.514
0.516
0.518
0.520
Cosine Similarity

(B)

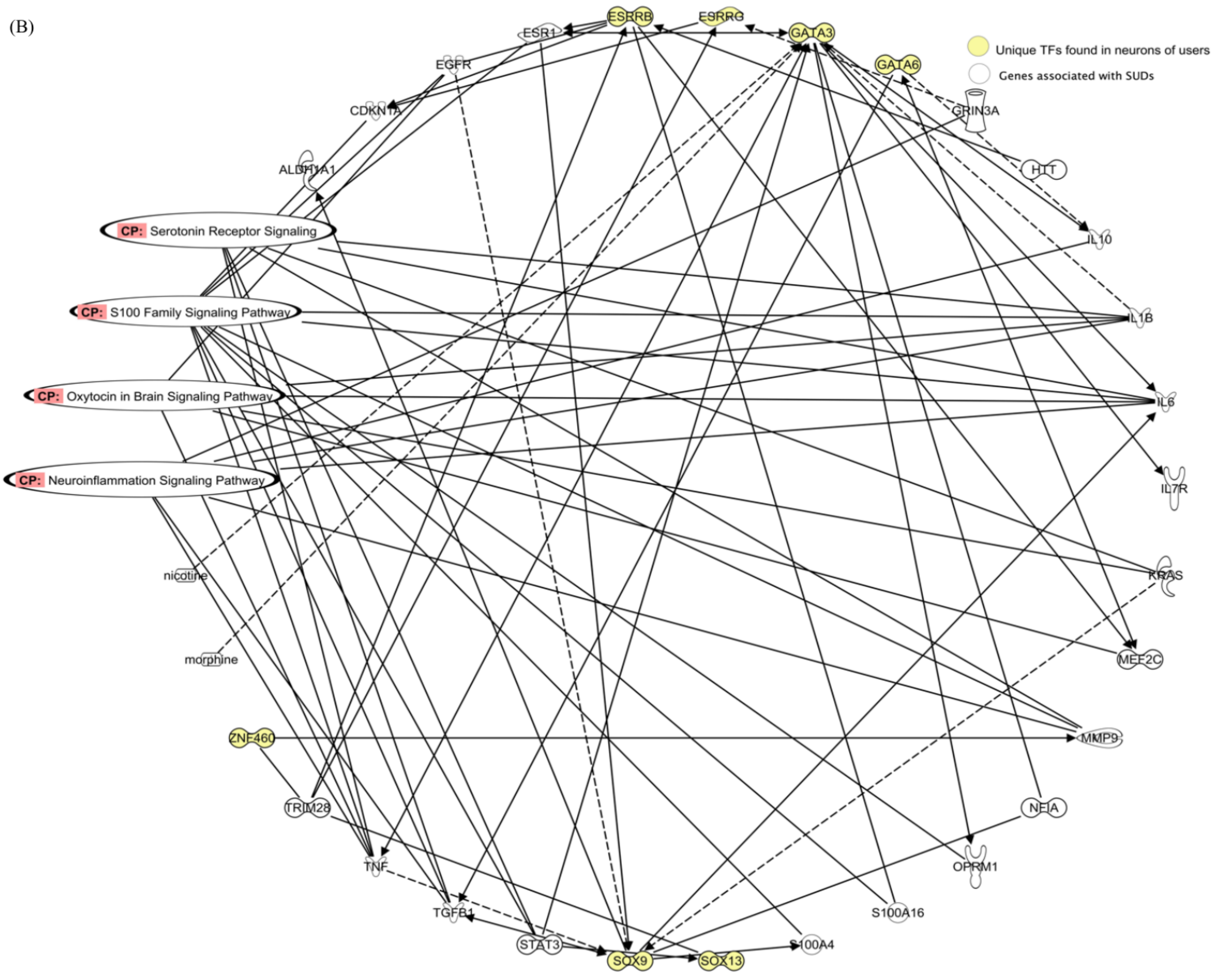
Unique TFs found in neurons of users
Genes associated with SUDs
ESRRB
ESRRG
GATA3
GATA6
ESR1
EGFR
CDKN1A
GRIN3A
ALDH1A1
HTT
IL10
CP: Serotonin Receptor Signaling
CP: S100 Family Signaling Pathway
IL1B
CP: Oxytocin in Brain Signaling Pathway
IL6
CP: Neuroinflammation Signaling Pathway
IL7R
nicotine
KRAS
morphine
MEF2C
ZNF460
MMP9
TRIM28
NFIA
TNF
OPRM1
TGFB1
S100A16
STAT3
SOX9
SOX13
S100A4

(A)

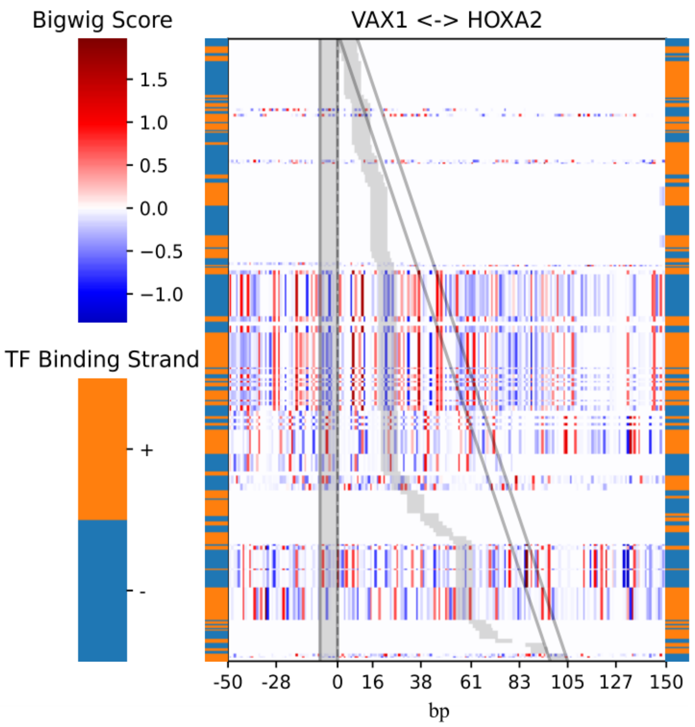
Bigwig Score
1.5
1.0
0.5
0.0
−0.5
−1.0
TF Binding Strand
+
-
VAX1 <-> HOXA2
-50 -28 0 16 38 61 83 105 127 150
bp

(B)

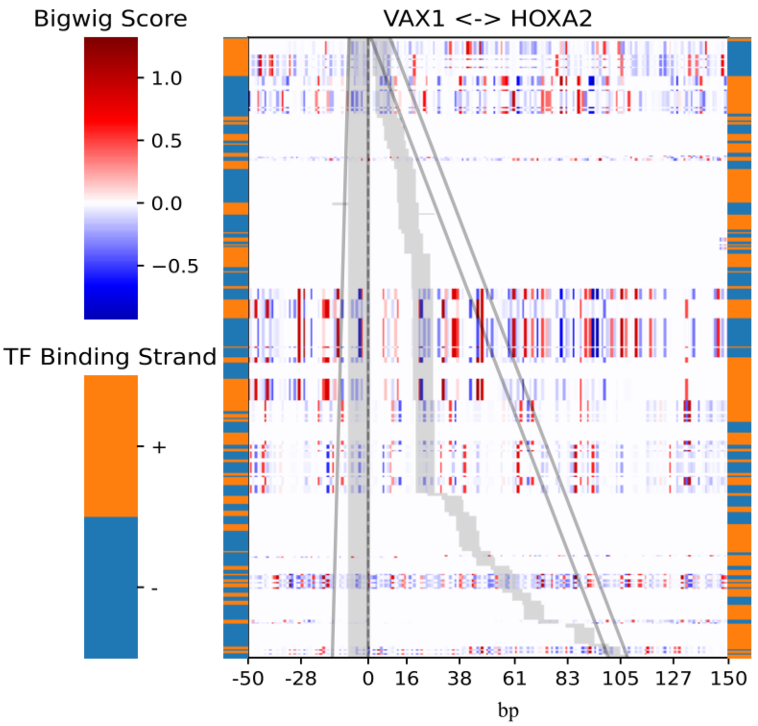
Bigwig Score
1.0
0.5
0.0
−0.5
TF Binding Strand
+
-
VAX1 <-> HOXA2
-50 -28 0 16 38 61 83 105 127 150
bp

(C)

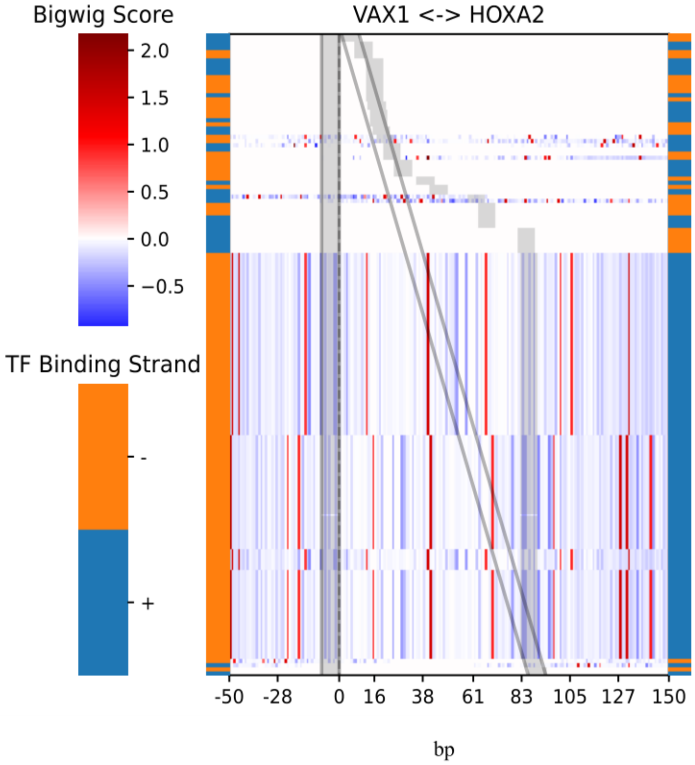
Bigwig Score
2.0
1.5
1.0
0.5
0.0
−0.5
TF Binding Strand
-
+
VAX1 <-> HOXA2
-50 -28 0 16 38 61 83 105 127 150
bp

(D)

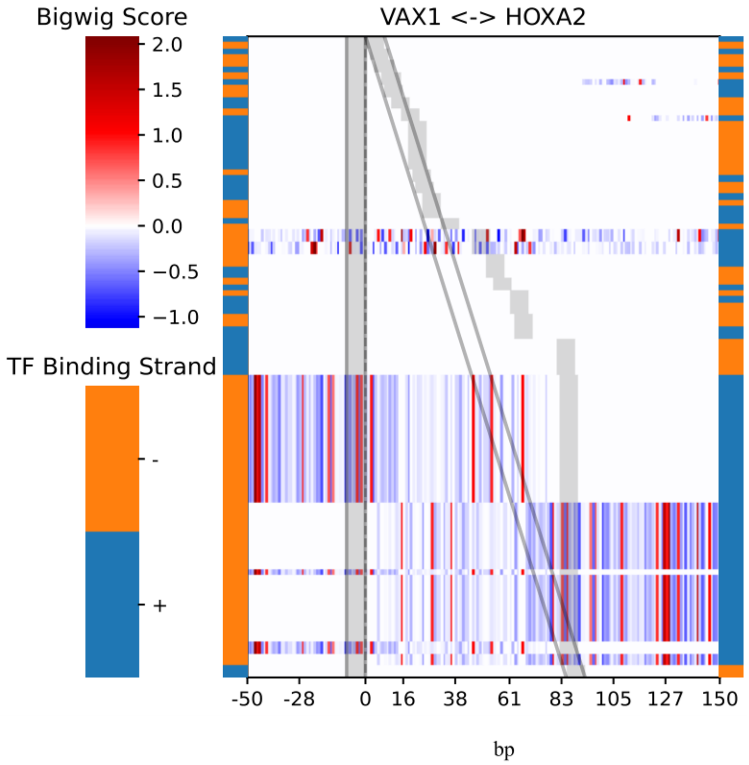
Bigwig Score
2.0
1.5
1.0
0.5
0.0
−0.5
−1.0
TF Binding Strand
-
+
VAX1 <-> HOXA2
-50 -28 0 16 38 61 83 105 127 150
bp
