# Supplementary material for: Heroin addiction modulates transcription factor binding in regulatory regions of the human putamen
Source: Sci Rep. 2026 May 12;16:21737. doi: 10.1038/s41598-026-52754-7 (PMC13357565; doi:10.1038/s41598-026-52754-7)
Supplement: Supplementary file 3 — Supplementary Material 3 [file 41598_2026_52754_MOESM3_ESM.pdf]

Supplemental Information
Footprint Glia (Merged)

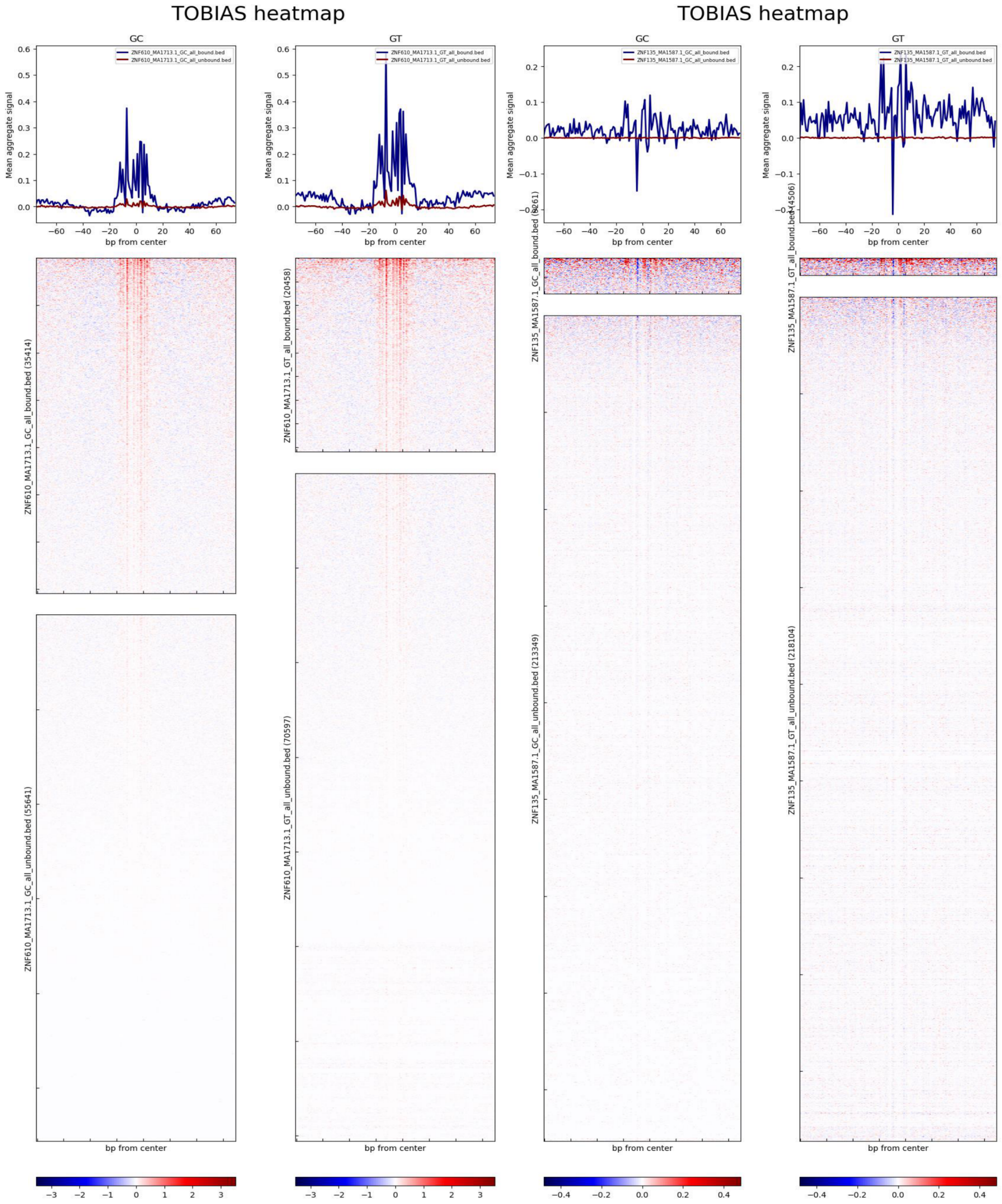
TOBIAS heatmap
TOBIAS heatmap
GC
GT
GC
GT
ZNF610_MA1713.1_GC_all_bound.bed
ZNF610_MA1713.1_GC_all_unbound.bed
ZNF610_MA1713.1_GT_all_bound.bed
ZNF610_MA1713.1_GT_all_unbound.bed
ZNF135_MA1587.1_GC_all_bound.bed
ZNF135_MA1587.1_GC_all_unbound.bed
ZNF135_MA1587.1_GT_all_bound.bed
ZNF135_MA1587.1_GT_all_unbound.bed
Mean aggregate signal
bp from center
ZNF610_MA1713.1_GC_all_bound.bed (35414)
ZNF610_MA1713.1_GC_all_unbound.bed (55641)
ZNF610_MA1713.1_GT_all_bound.bed (20458)
ZNF610_MA1713.1_GT_all_unbound.bed (70597)
ZNF135_MA1587.1_GC_all_unbound.bed (213349)
ZNF135_MA1587.1_GT_all_unbound.bed (218104)

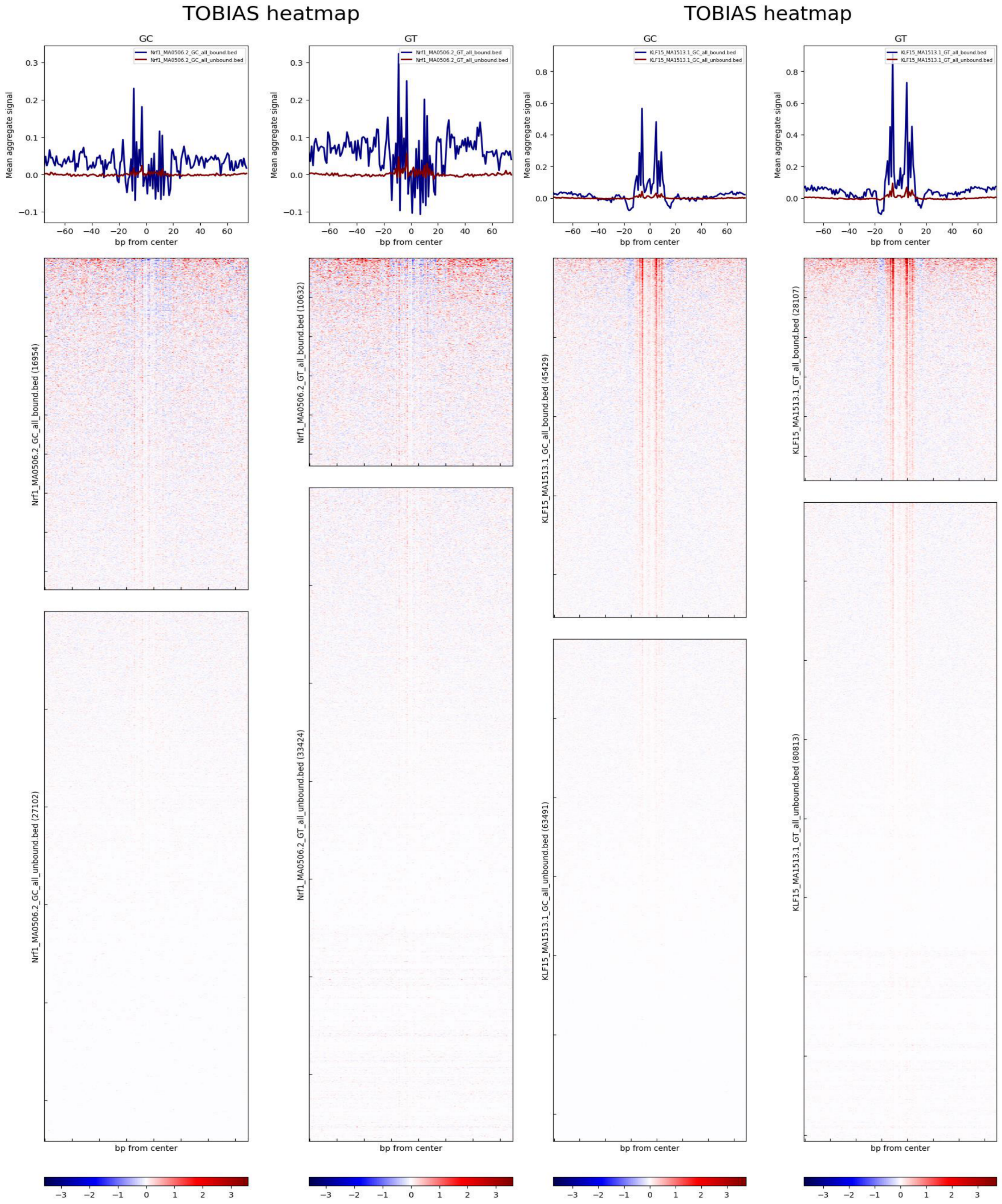

TOBIAS heatmap
TOBIAS heatmap
GC
GT
GC
GT
Nrf1_MA0506.2_GC_all_bound.bed
Nrf1_MA0506.2_GC_all_unbound.bed
Nrf1_MA0506.2_GT_all_bound.bed
Nrf1_MA0506.2_GT_all_unbound.bed
KLF15_MA1513.1_GC_all_bound.bed
KLF15_MA1513.1_GC_all_unbound.bed
KLF15_MA1513.1_GT_all_bound.bed
KLF15_MA1513.1_GT_all_unbound.bed
Mean aggregate signal
bp from center
Nrf1_MA0506.2_GC_all_bound.bed (16954)
Nrf1_MA0506.2_GC_all_unbound.bed (27102)
Nrf1_MA0506.2_GT_all_bound.bed (10632)
Nrf1_MA0506.2_GT_all_unbound.bed (33424)
KLF15_MA1513.1_GC_all_bound.bed (45429)
KLF15_MA1513.1_GC_all_unbound.bed (63491)
KLF15_MA1513.1_GT_all_bound.bed (28107)
KLF15_MA1513.1_GT_all_unbound.bed (80813)

Supplemental Information
Footprint Neuron (Merged)

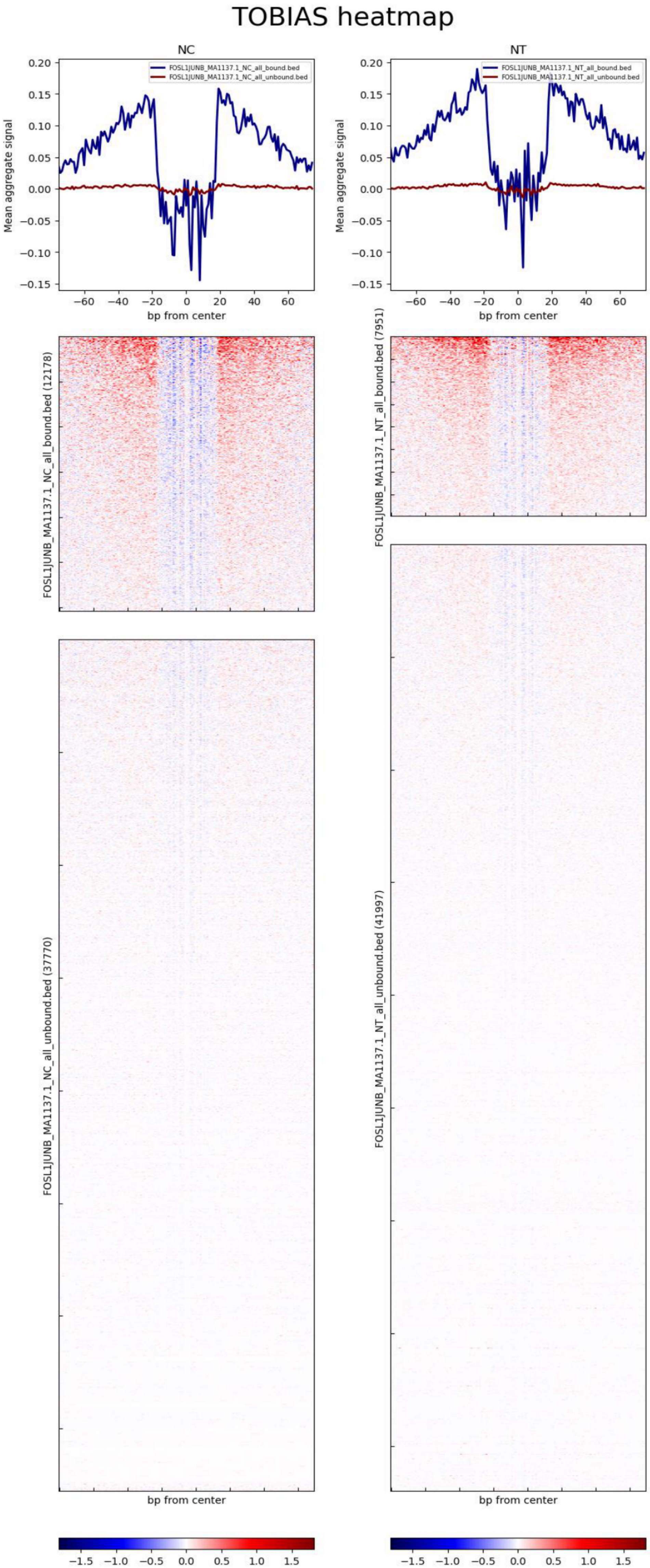
TOBIAS heatmap
NC
FOSL1JUNB_MA1137.1_NC_all_bound.bed
FOSL1JUNB_MA1137.1_NC_all_unbound.bed
NT
FOSL1JUNB_MA1137.1_NT_all_bound.bed
FOSL1JUNB_MA1137.1_NT_all_unbound.bed
Mean aggregate signal
0.20
0.15
0.10
0.05
0.00
−0.05
−0.10
−0.15
−60
−40
−20
0
20
40
60
bp from center
FOSL1JUNB_MA1137.1_NC_all_bound.bed (12178)
FOSL1JUNB_MA1137.1_NC_all_unbound.bed (37770)
FOSL1JUNB_MA1137.1_NT_all_bound.bed (7951)
FOSL1JUNB_MA1137.1_NT_all_unbound.bed (41997)
−1.5
−1.0
−0.5
0.0
0.5
1.0
1.5

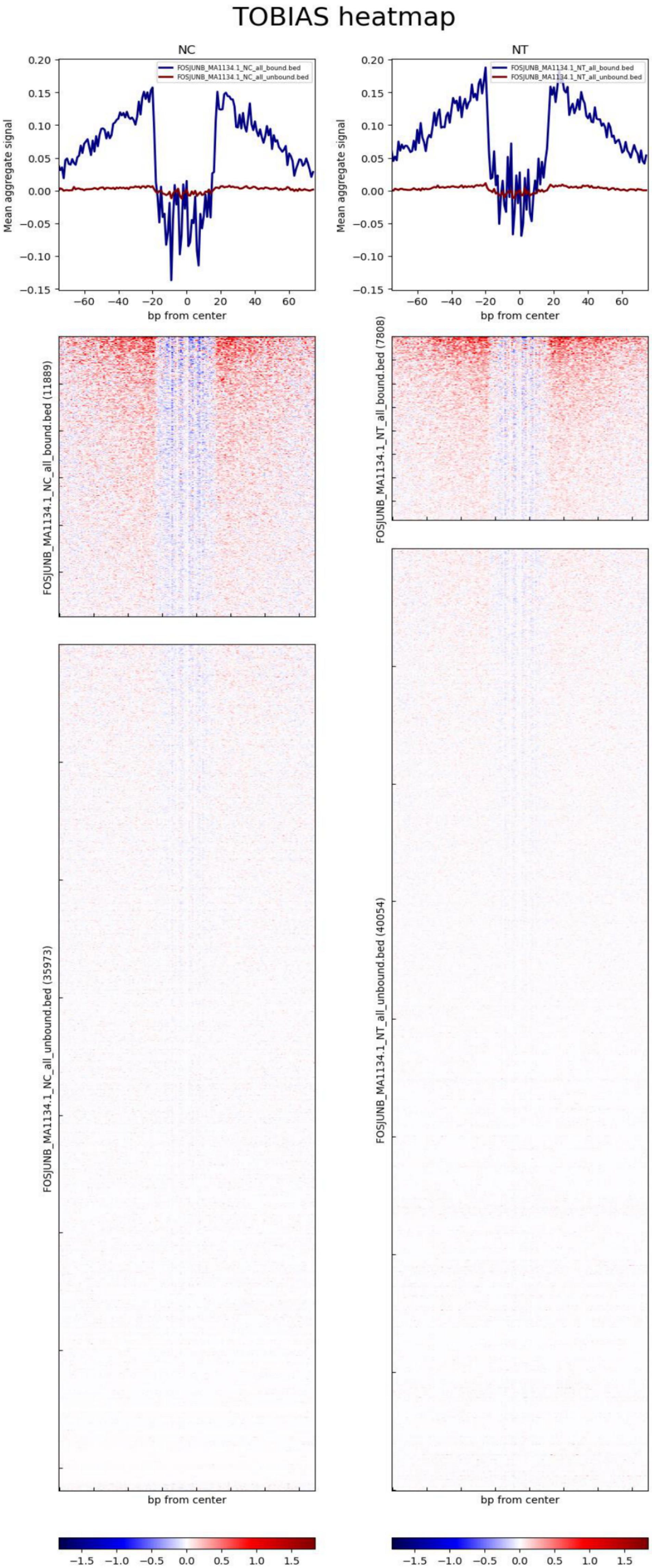
TOBIAS heatmap
NC
FOSJUNB_MA1134.1_NC_all_bound.bed
FOSJUNB_MA1134.1_NC_all_unbound.bed
NT
FOSJUNB_MA1134.1_NT_all_bound.bed
FOSJUNB_MA1134.1_NT_all_unbound.bed
Mean aggregate signal
0.20
0.15
0.10
0.05
0.00
−0.05
−0.10
−0.15
−60
−40
−20
0
20
40
60
bp from center
FOSJUNB_MA1134.1_NC_all_bound.bed (11889)
FOSJUNB_MA1134.1_NC_all_unbound.bed (35973)
FOSJUNB_MA1134.1_NT_all_bound.bed (7808)
FOSJUNB_MA1134.1_NT_all_unbound.bed (40054)
−1.5
−1.0
−0.5
0.0
0.5
1.0
1.5

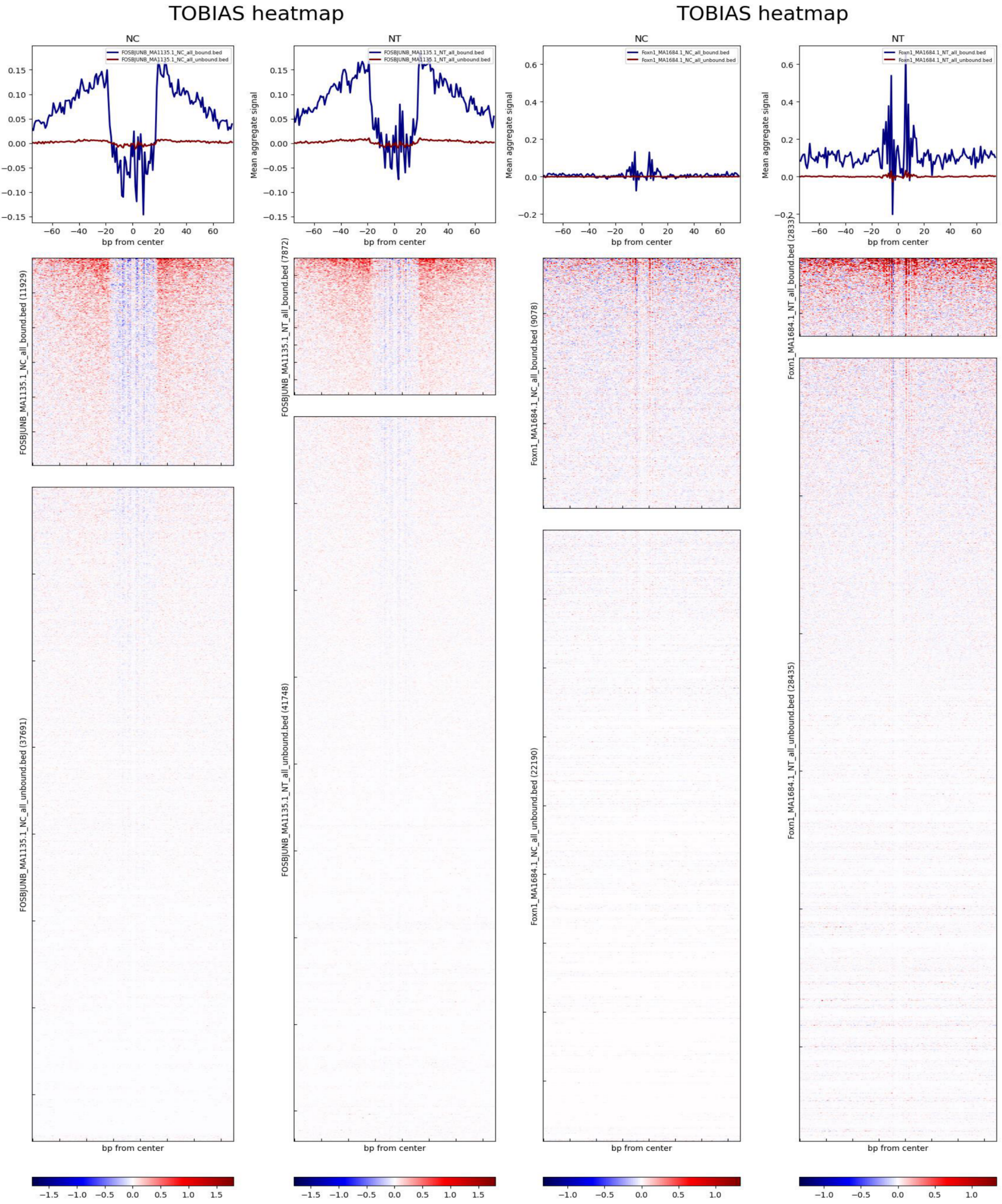

TOBIAS heatmap
TOBIAS heatmap
NC
NT
NC
NT
FOSBJUNB_MA1135.1_NC_all_bound.bed
FOSBJUNB_MA1135.1_NC_all_unbound.bed
FOSBJUNB_MA1135.1_NT_all_bound.bed
FOSBJUNB_MA1135.1_NT_all_unbound.bed
Foxn1_MA1684.1_NC_all_bound.bed
Foxn1_MA1684.1_NC_all_unbound.bed
Foxn1_MA1684.1_NT_all_bound.bed
Foxn1_MA1684.1_NT_all_unbound.bed
Mean aggregate signal
bp from center
FOSBJUNB_MA1135.1_NC_all_bound.bed (11929)
FOSBJUNB_MA1135.1_NC_all_unbound.bed (37691)
FOSBJUNB_MA1135.1_NT_all_bound.bed (7872)
FOSBJUNB_MA1135.1_NT_all_unbound.bed (41748)
Foxn1_MA1684.1_NC_all_bound.bed (9078)
Foxn1_MA1684.1_NC_all_unbound.bed (22190)
Foxn1_MA1684.1_NT_all_bound.bed (2833)
Foxn1_MA1684.1_NT_all_unbound.bed (28435)

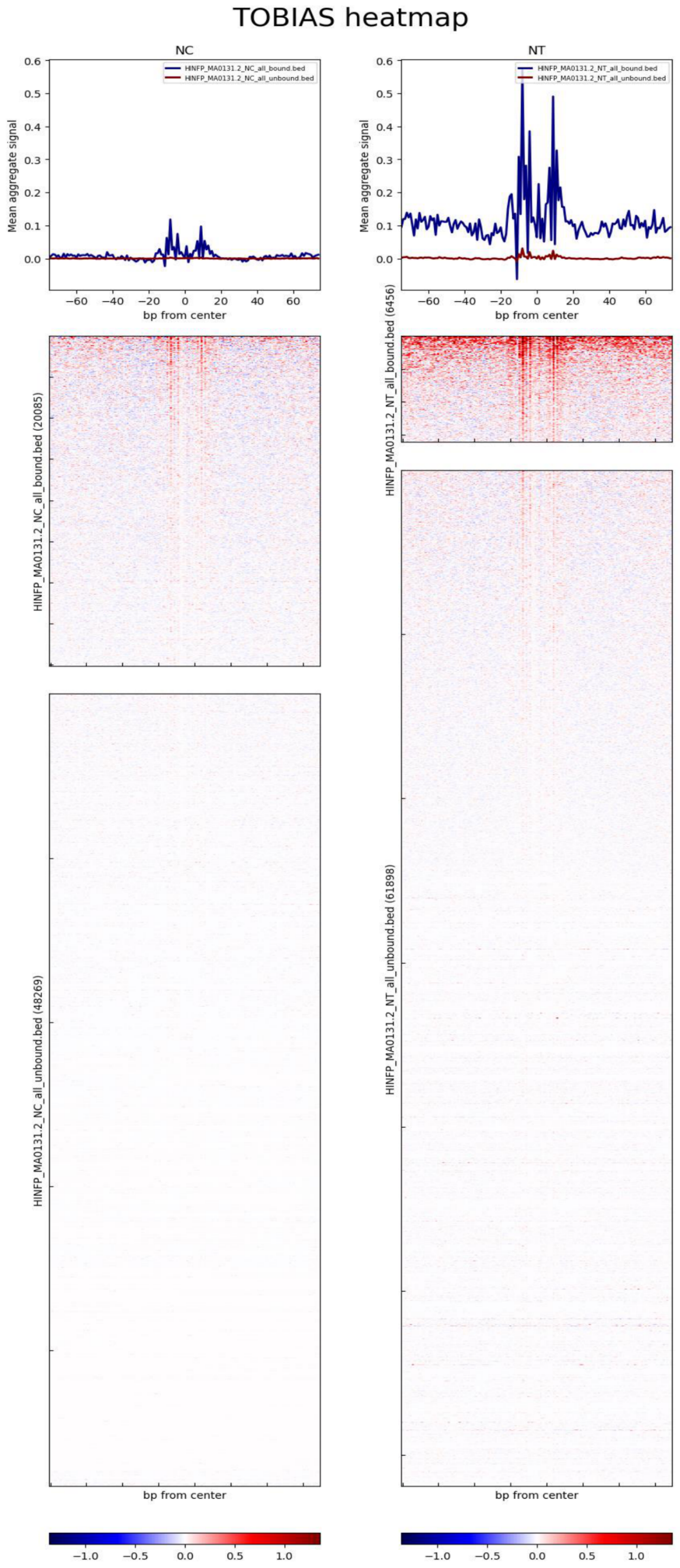
TOBIAS heatmap
NC
NT
Mean aggregate signal
HINFP_MA0131.2_NC_all_bound.bed
HINFP_MA0131.2_NC_all_unbound.bed
HINFP_MA0131.2_NT_all_bound.bed
HINFP_MA0131.2_NT_all_unbound.bed
bp from center
HINFP_MA0131.2_NC_all_bound.bed (20085)
HINFP_MA0131.2_NC_all_unbound.bed (48269)
HINFP_MA0131.2_NT_all_bound.bed (6456)
HINFP_MA0131.2_NT_all_unbound.bed (61898)

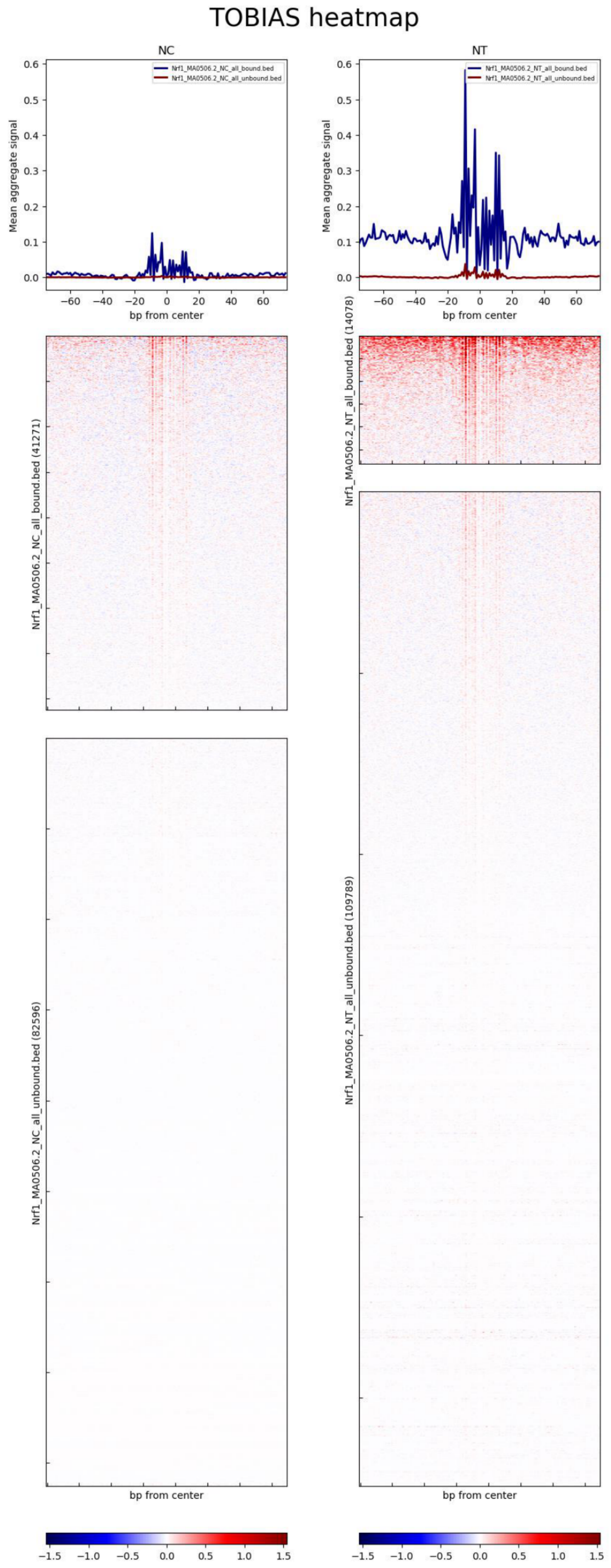
TOBIAS heatmap
NC
NT
Mean aggregate signal
Nrf1_MA0506.2_NC_all_bound.bed
Nrf1_MA0506.2_NC_all_unbound.bed
Nrf1_MA0506.2_NT_all_bound.bed
Nrf1_MA0506.2_NT_all_unbound.bed
bp from center
Nrf1_MA0506.2_NC_all_bound.bed (41271)
Nrf1_MA0506.2_NC_all_unbound.bed (82596)
Nrf1_MA0506.2_NT_all_bound.bed (14078)
Nrf1_MA0506.2_NT_all_unbound.bed (109789)

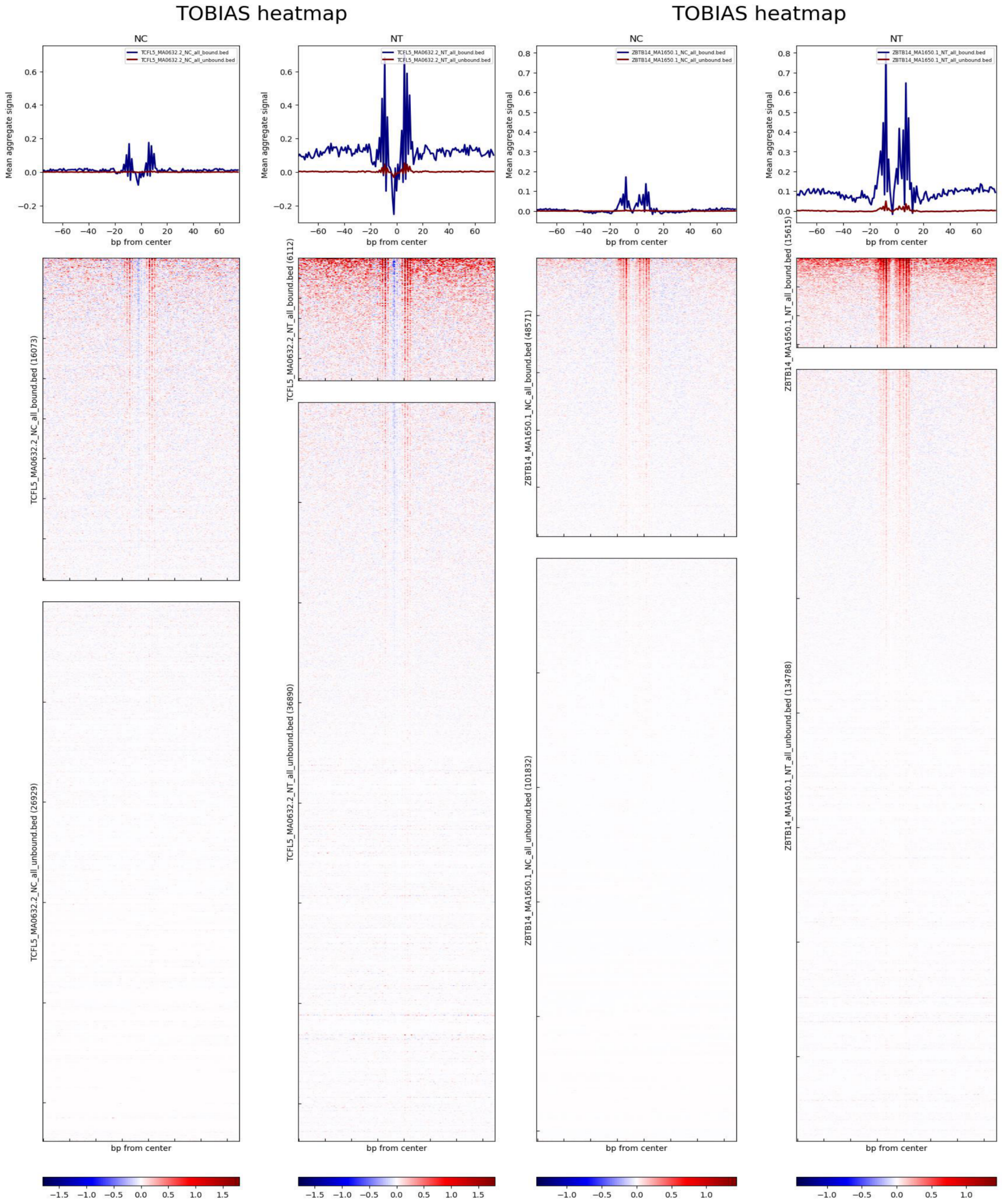

TOBIAS heatmap
TOBIAS heatmap
NC
NT
NC
NT
Mean aggregate signal
bp from center
TCFL5_MA0632.2_NC_all_bound.bed (16073)
TCFL5_MA0632.2_NC_all_unbound.bed (26929)
TCFL5_MA0632.2_NT_all_bound.bed (6112)
TCFL5_MA0632.2_NT_all_unbound.bed (36890)
ZBTB14_MA1650.1_NC_all_bound.bed (48571)
ZBTB14_MA1650.1_NC_all_unbound.bed (101832)
ZBTB14_MA1650.1_NT_all_bound.bed (15615)
ZBTB14_MA1650.1_NT_all_unbound.bed (134788)

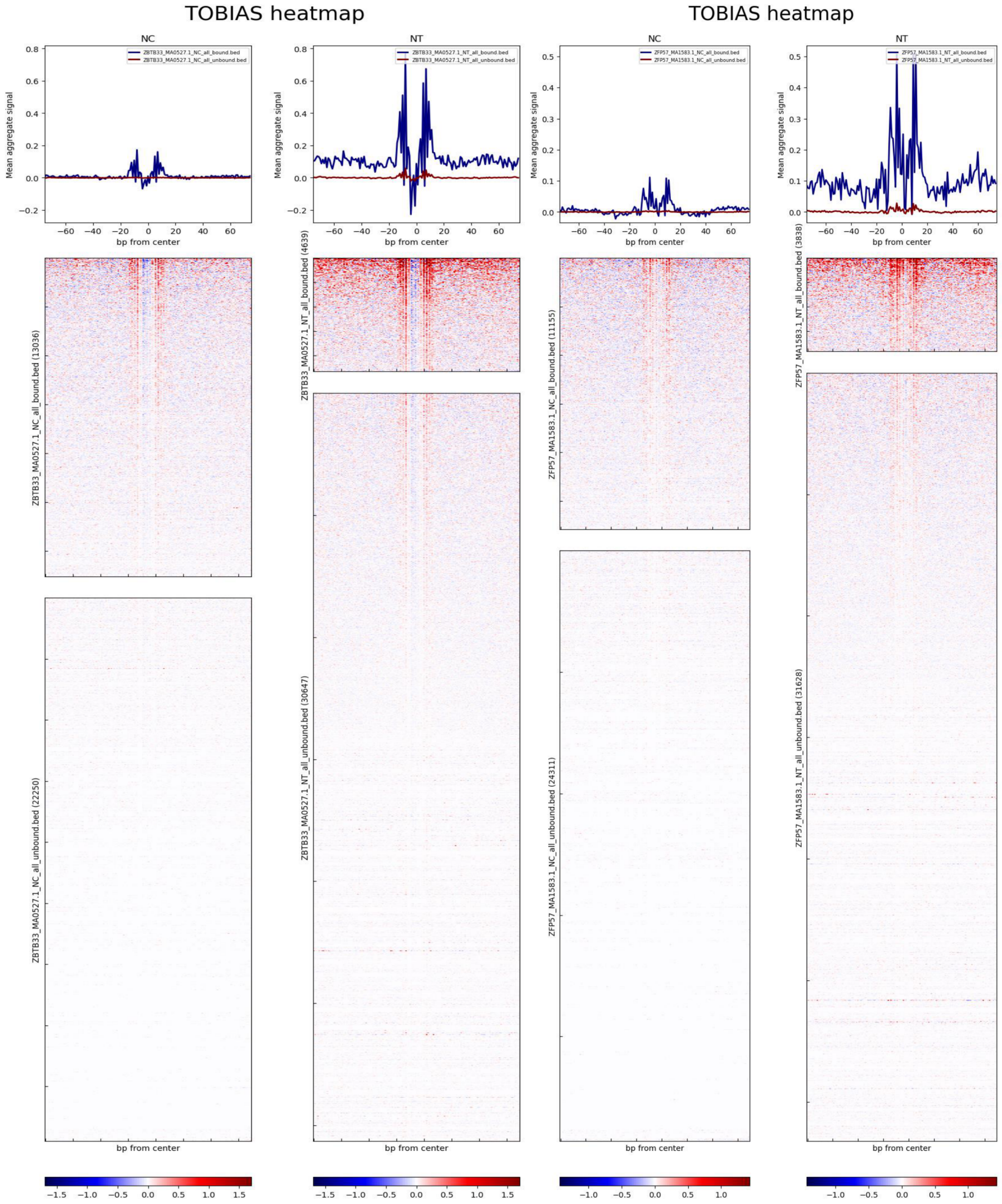
TOBIAS heatmap
TOBIAS heatmap
NC
NT
NC
NT
ZBTB33_MA0527.1_NC_all_bound.bed
ZBTB33_MA0527.1_NC_all_unbound.bed
ZBTB33_MA0527.1_NT_all_bound.bed
ZBTB33_MA0527.1_NT_all_unbound.bed
ZFP57_MA1583.1_NC_all_bound.bed
ZFP57_MA1583.1_NC_all_unbound.bed
ZFP57_MA1583.1_NT_all_bound.bed
ZFP57_MA1583.1_NT_all_unbound.bed
Mean aggregate signal
bp from center
ZBTB33_MA0527.1_NC_all_bound.bed (13036)
ZBTB33_MA0527.1_NC_all_unbound.bed (22250)
ZBTB33_MA0527.1_NT_all_bound.bed (4639)
ZBTB33_MA0527.1_NT_all_unbound.bed (30647)
ZFP57_MA1583.1_NC_all_bound.bed (11155)
ZFP57_MA1583.1_NC_all_unbound.bed (24311)
ZFP57_MA1583.1_NT_all_bound.bed (3838)
ZFP57_MA1583.1_NT_all_unbound.bed (31628)

TOBIAS heatmap

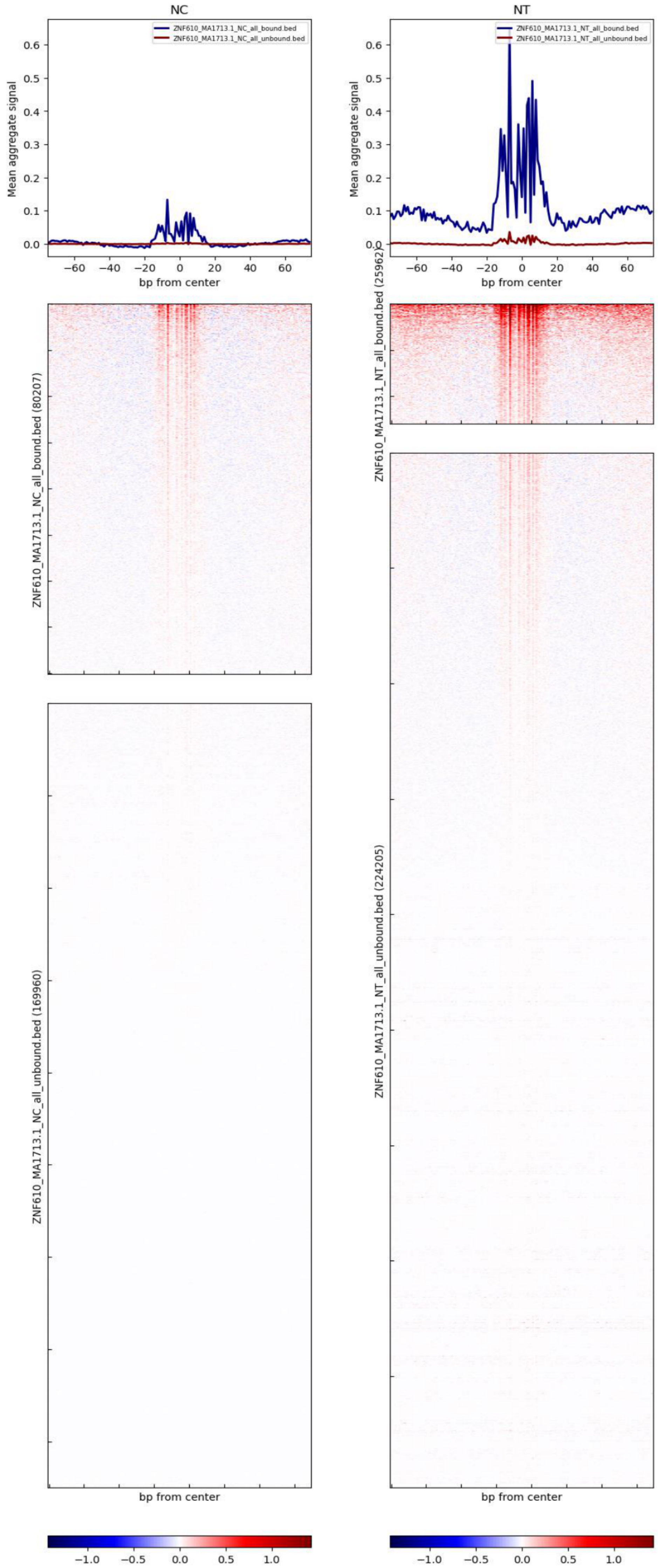

Supplemental Information: Unique TF pairs for user Neuron (merged)

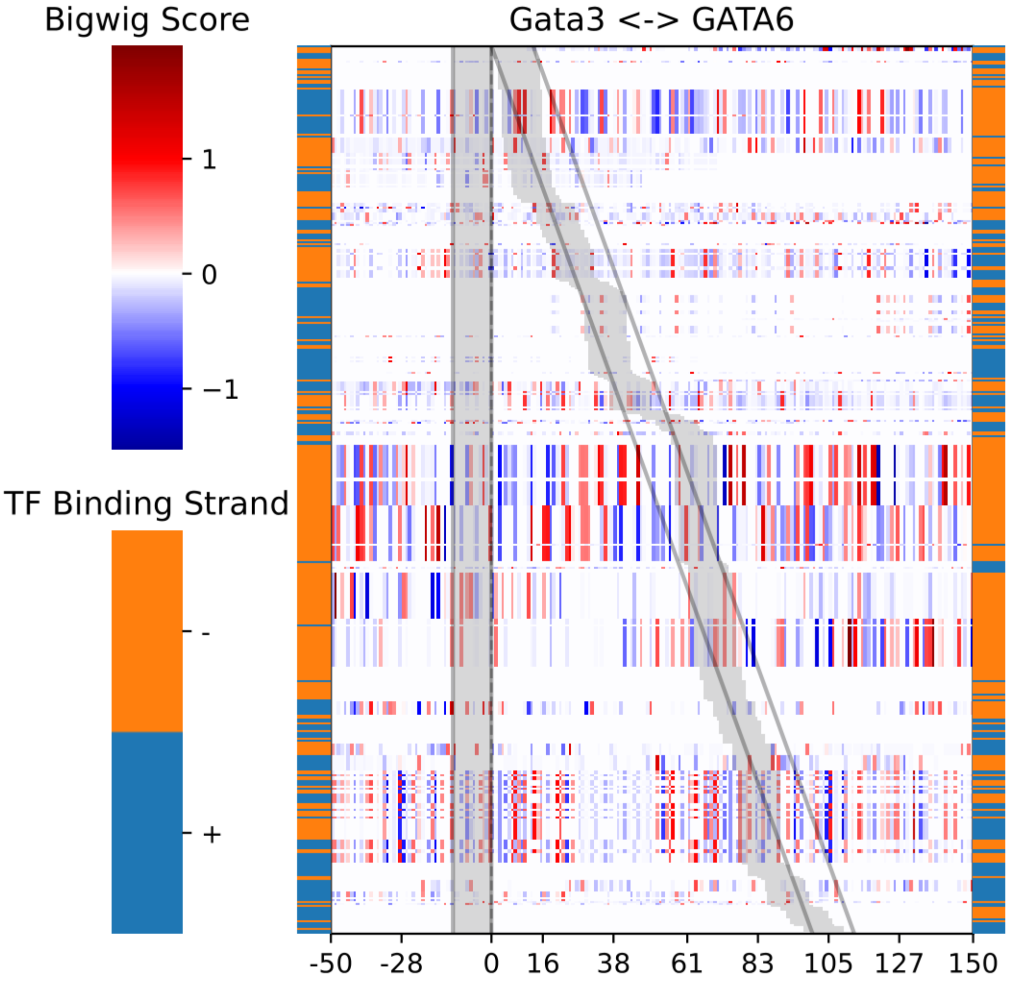
Bigwig Score
1
0
−1
TF Binding Strand
-
+
Gata3 <-> GATA6
-50
-28
0
16
38
61
83
105
127
150

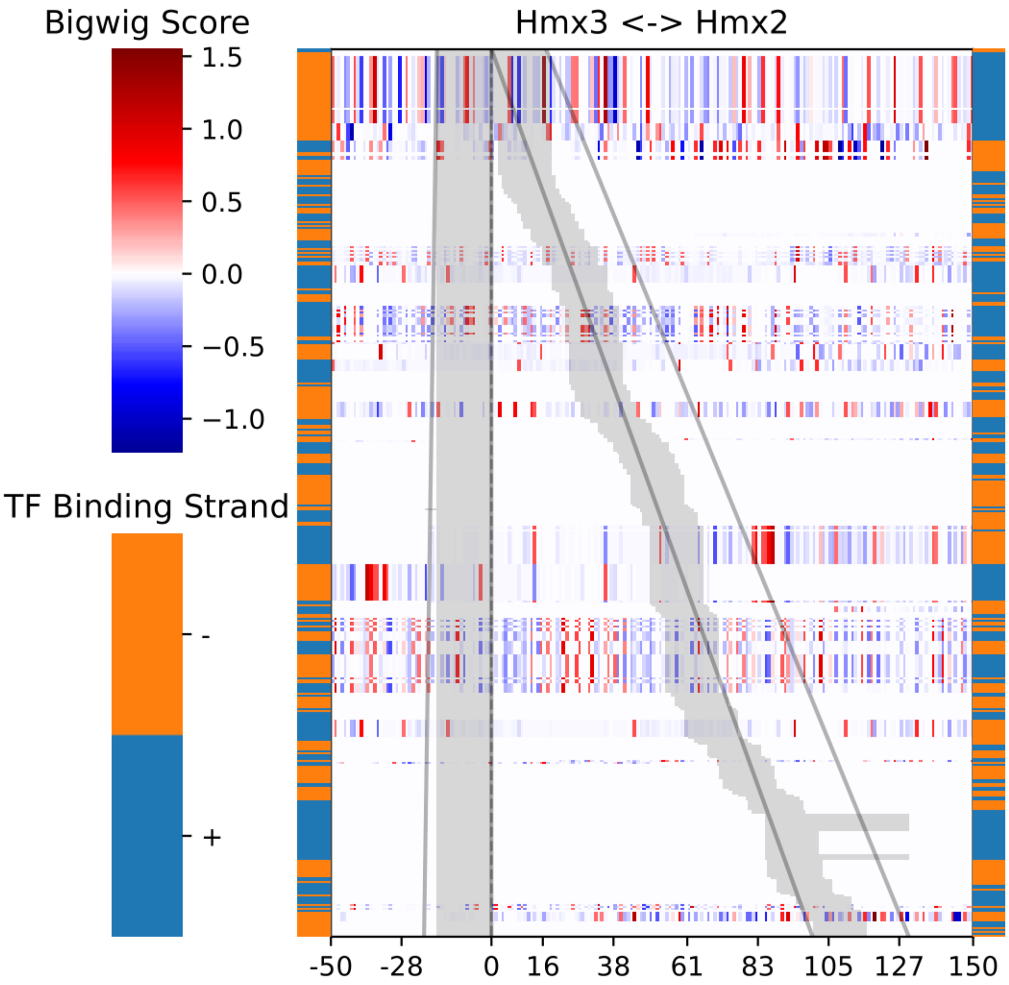

Bigwig Score
1.5
1.0
0.5
0.0
−0.5
−1.0
TF Binding Strand
-
+
Hmx3 <-> Hmx2
-50
-28
0
16
38
61
83
105
127
150

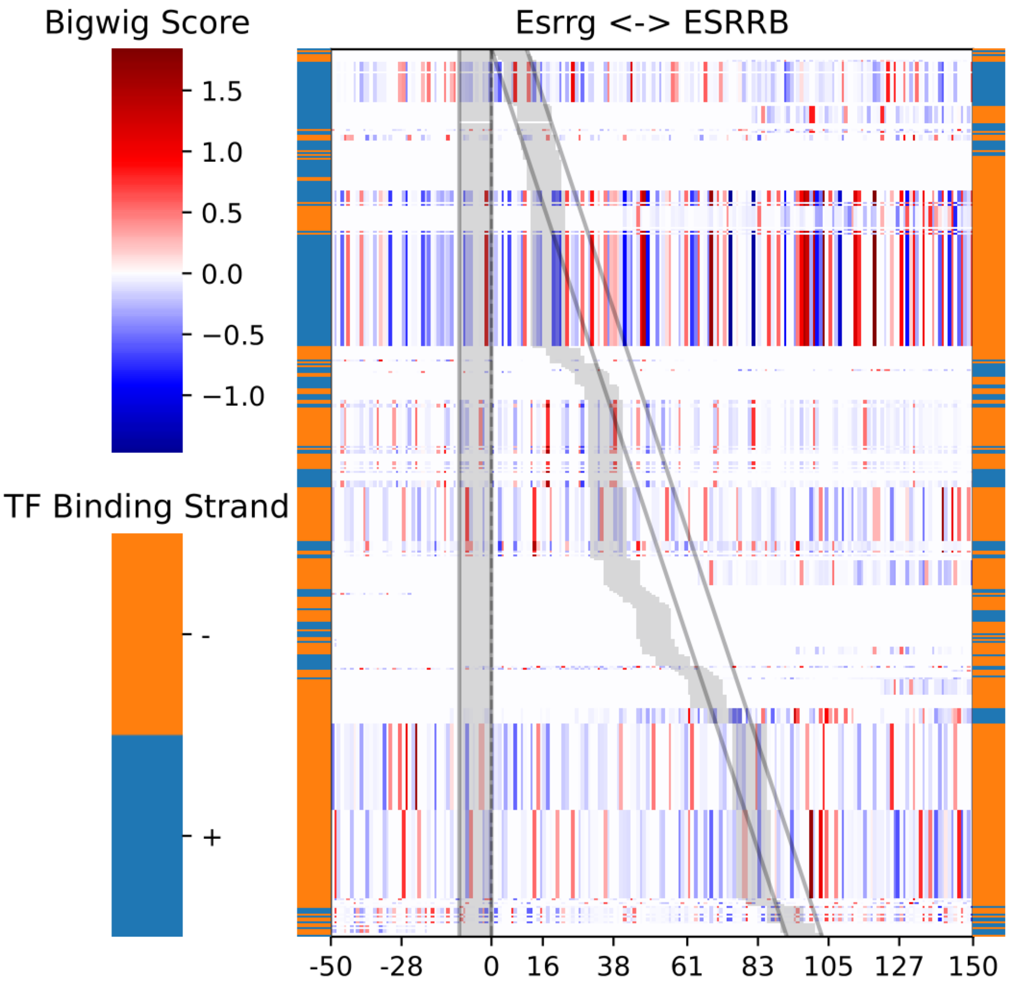
Bigwig Score
1.5
1.0
0.5
0.0
−0.5
−1.0
TF Binding Strand
-
+
Esrrg <-> ESRRB
-50
-28
0
16
38
61
83
105
127
150

Supplemental Information: Unique TF pairs for non-user Glia (merged)

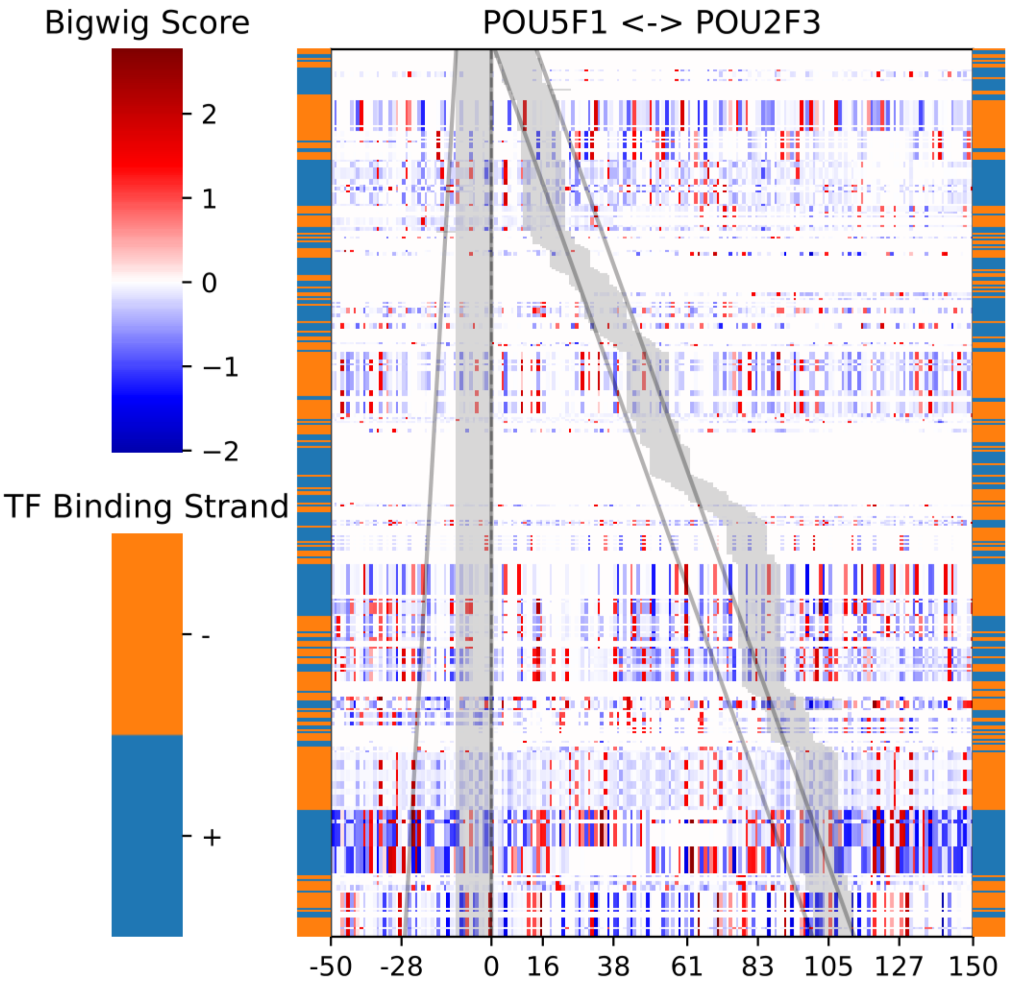

Bigwig Score
2
1
0
−1
−2
TF Binding Strand
-
+
POU5F1 <-> POU2F3
-50
-28
0
16
38
61
83
105
127
150

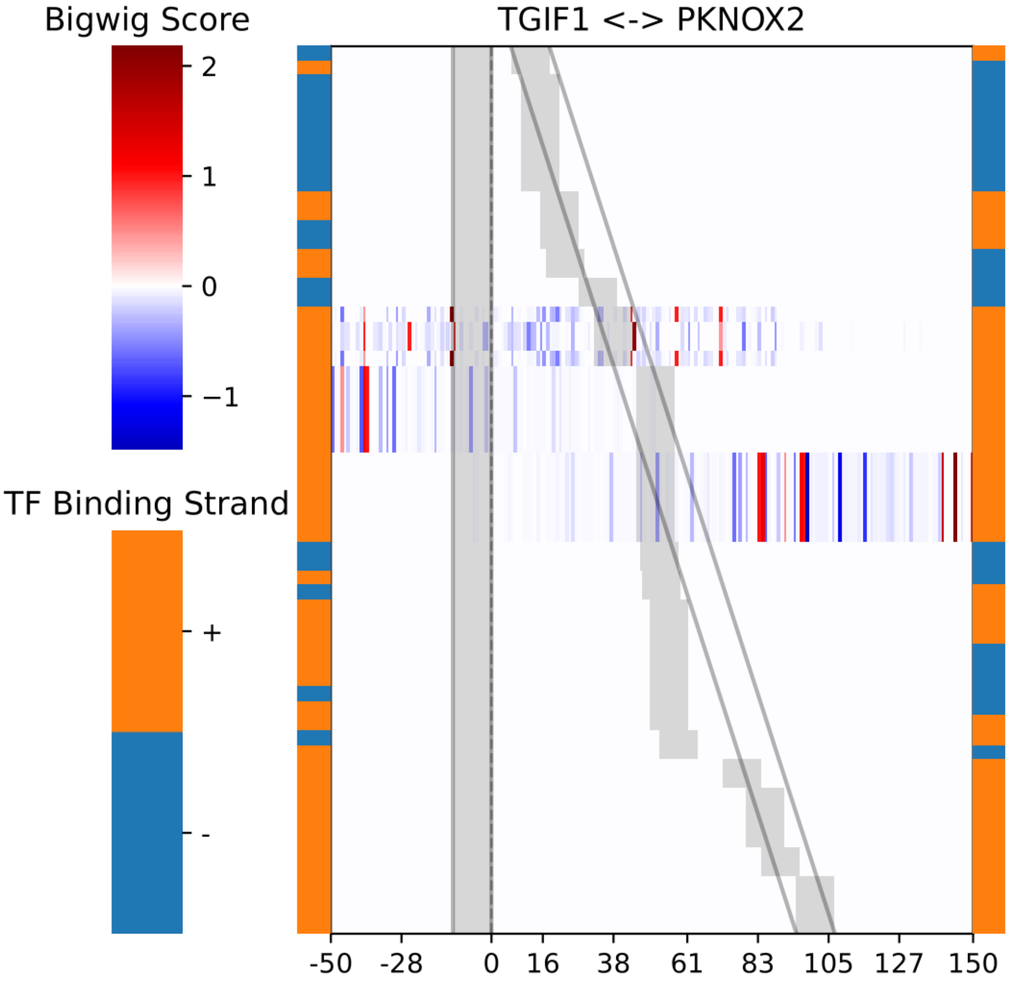

Bigwig Score
2
1
0
−1
TF Binding Strand
+
-
TGIF1 <-> PKNOX2
-50
-28
0
16
38
61
83
105
127
150

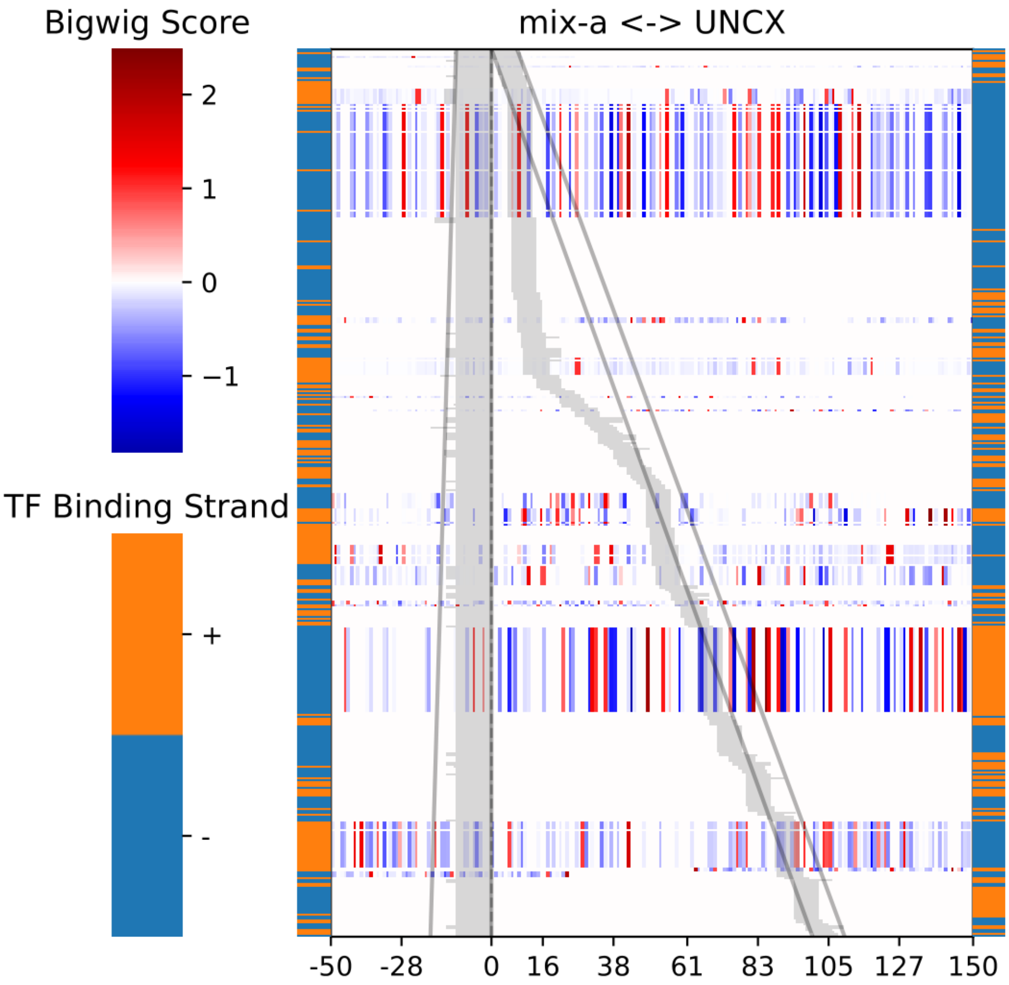

Bigwig Score
2
1
0
−1
TF Binding Strand
+
-
mix-a <-> UNCX
-50
-28
0
16
38
61
83
105
127
150

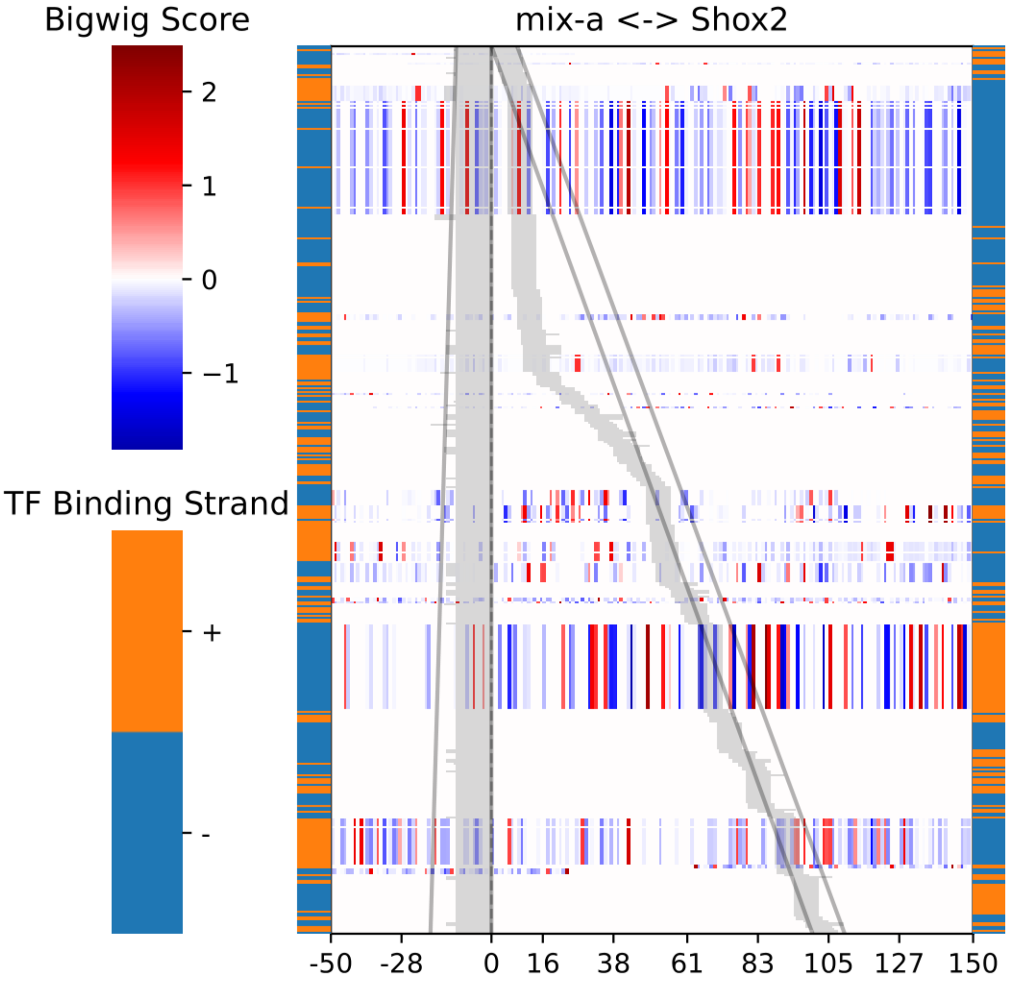

Bigwig Score
2
1
0
−1
TF Binding Strand
+
-
mix-a <-> Shox2
-50
-28
0
16
38
61
83
105
127
150

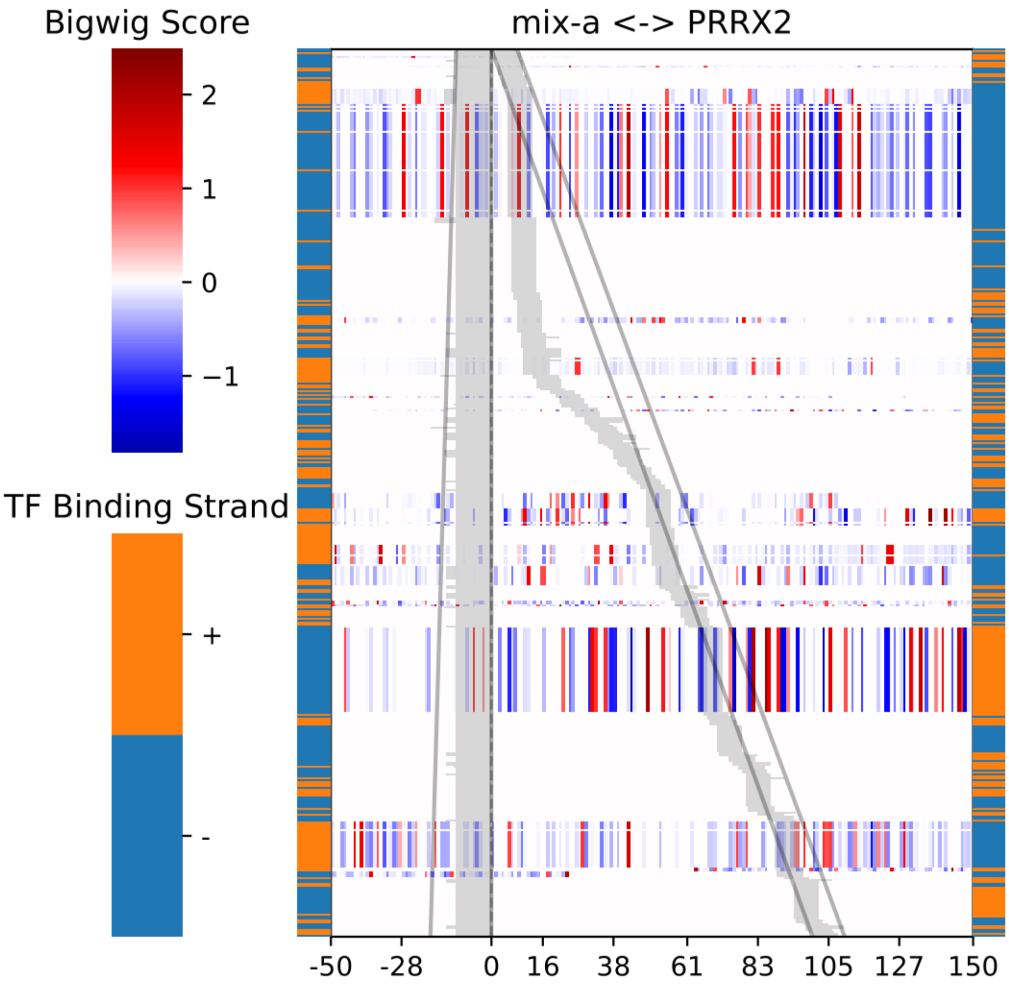

Bigwig Score
2
1
0
−1
TF Binding Strand
+
-
mix-a <-> PRRX2
-50
-28
0
16
38
61
83
105
127
150

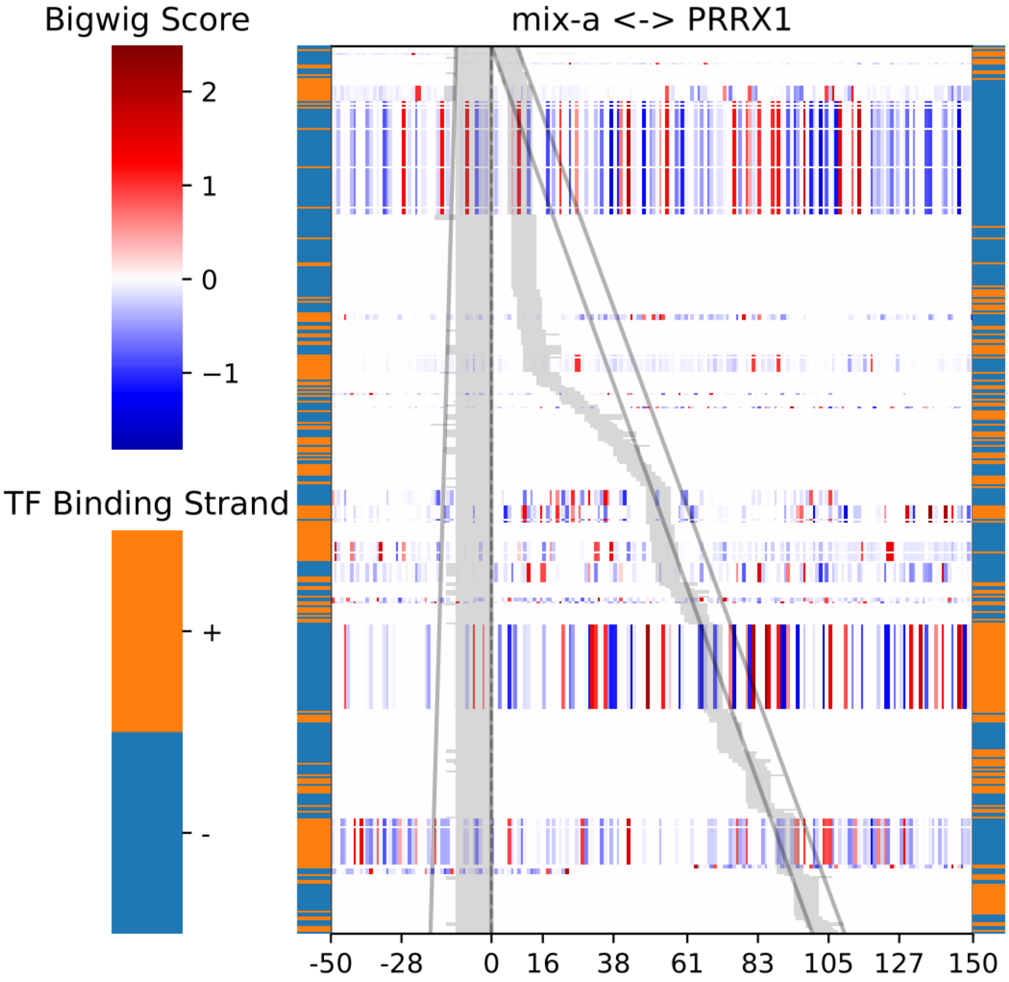

Bigwig Score
2
1
0
−1
TF Binding Strand
+
-
mix-a <-> PRRX1
-50
-28
0
16
38
61
83
105
127
150

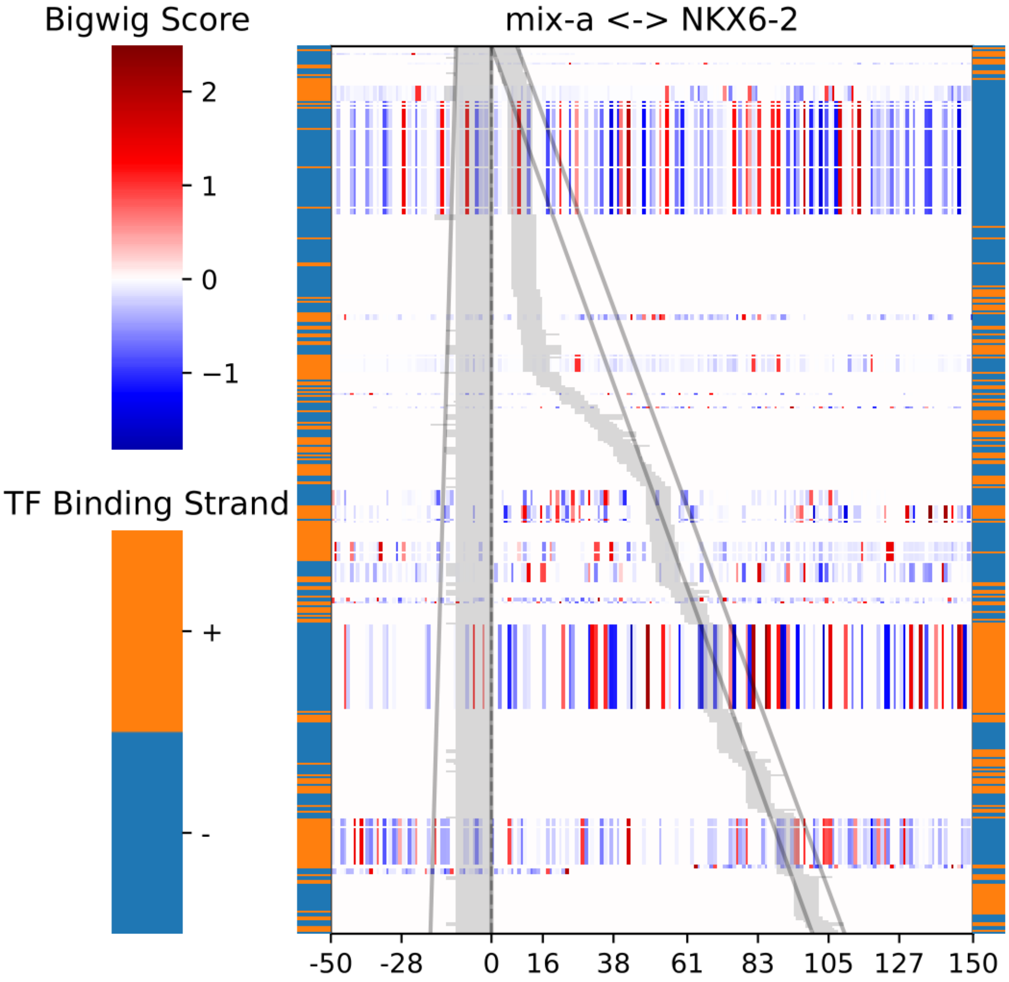

Bigwig Score
2
1
0
−1
TF Binding Strand
+
-
mix-a <-> NKX6-2
-50
-28
0
16
38
61
83
105
127
150

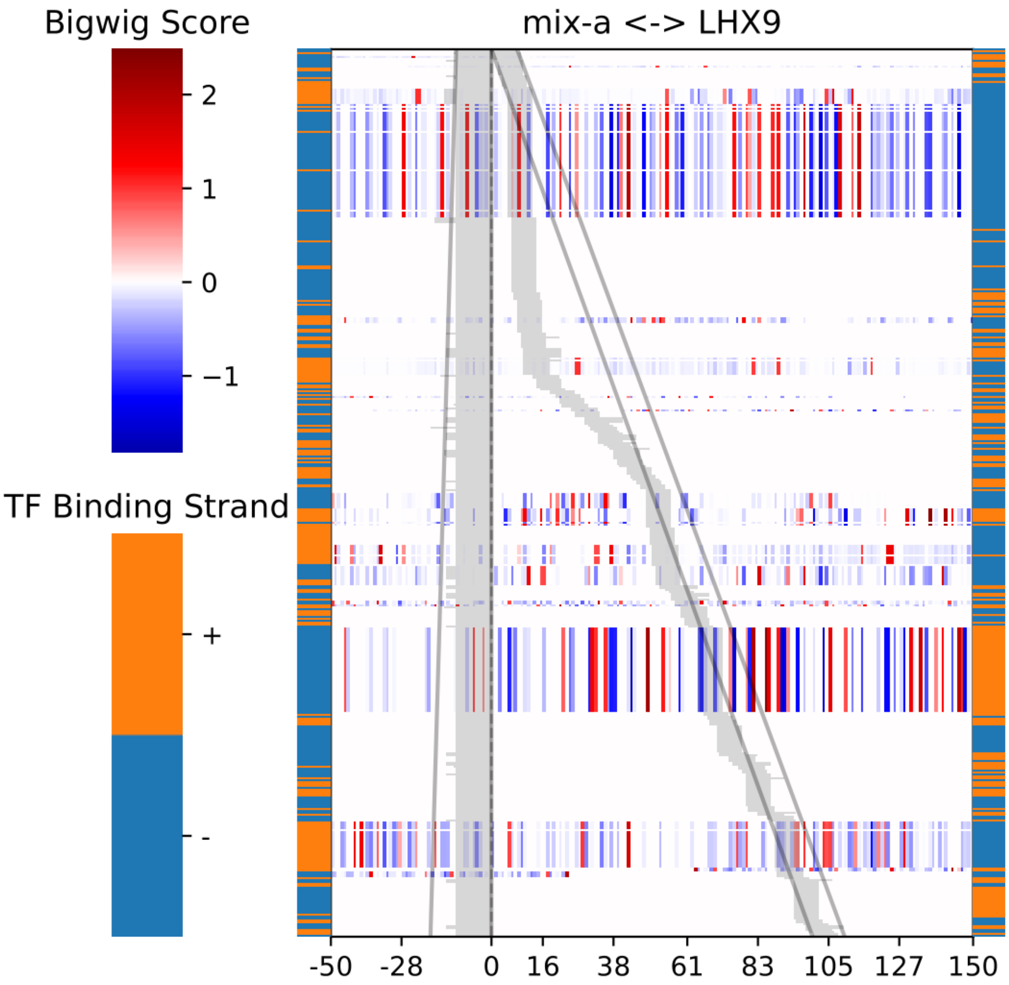
Bigwig Score
2
1
0
−1
TF Binding Strand
+
-
mix-a <-> LHX9
-50
-28
0
16
38
61
83
105
127
150

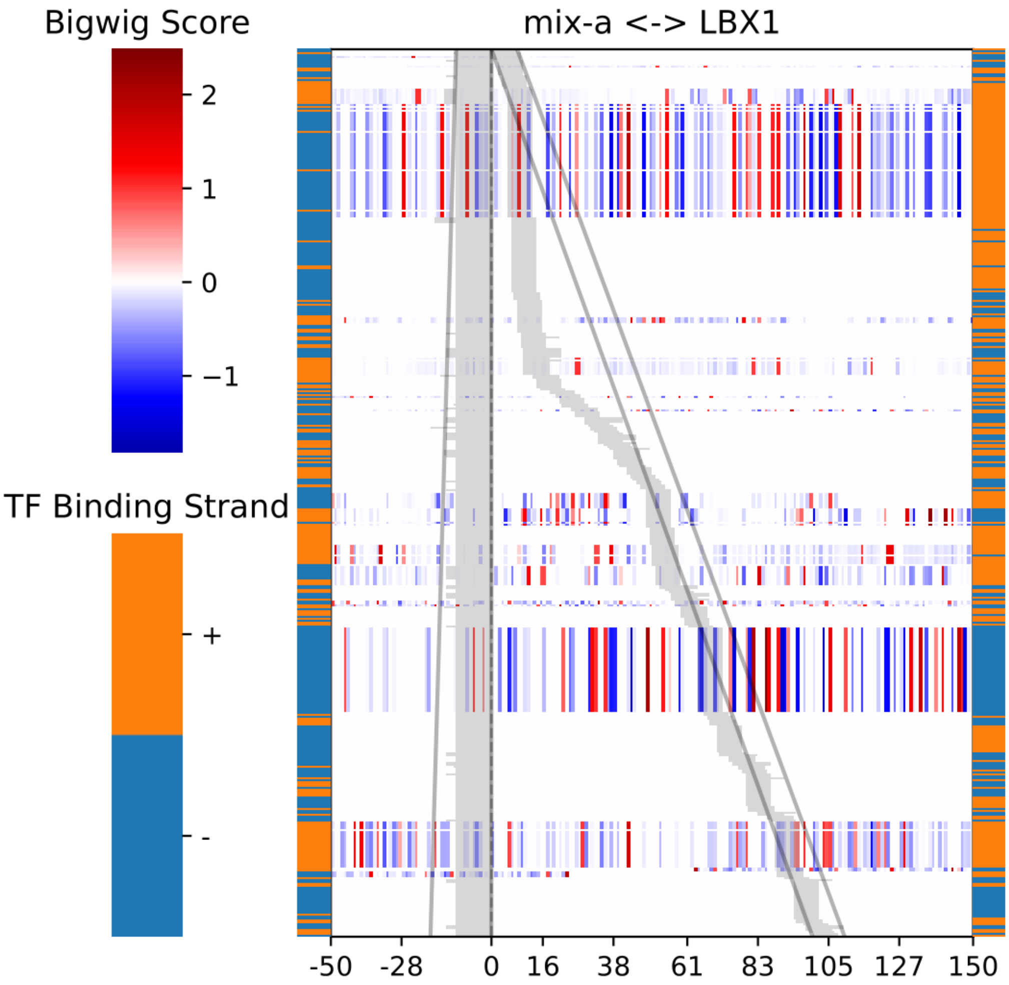

Bigwig Score
2
1
0
−1
TF Binding Strand
+
-
mix-a <-> LBX1
-50
-28
0
16
38
61
83
105
127
150

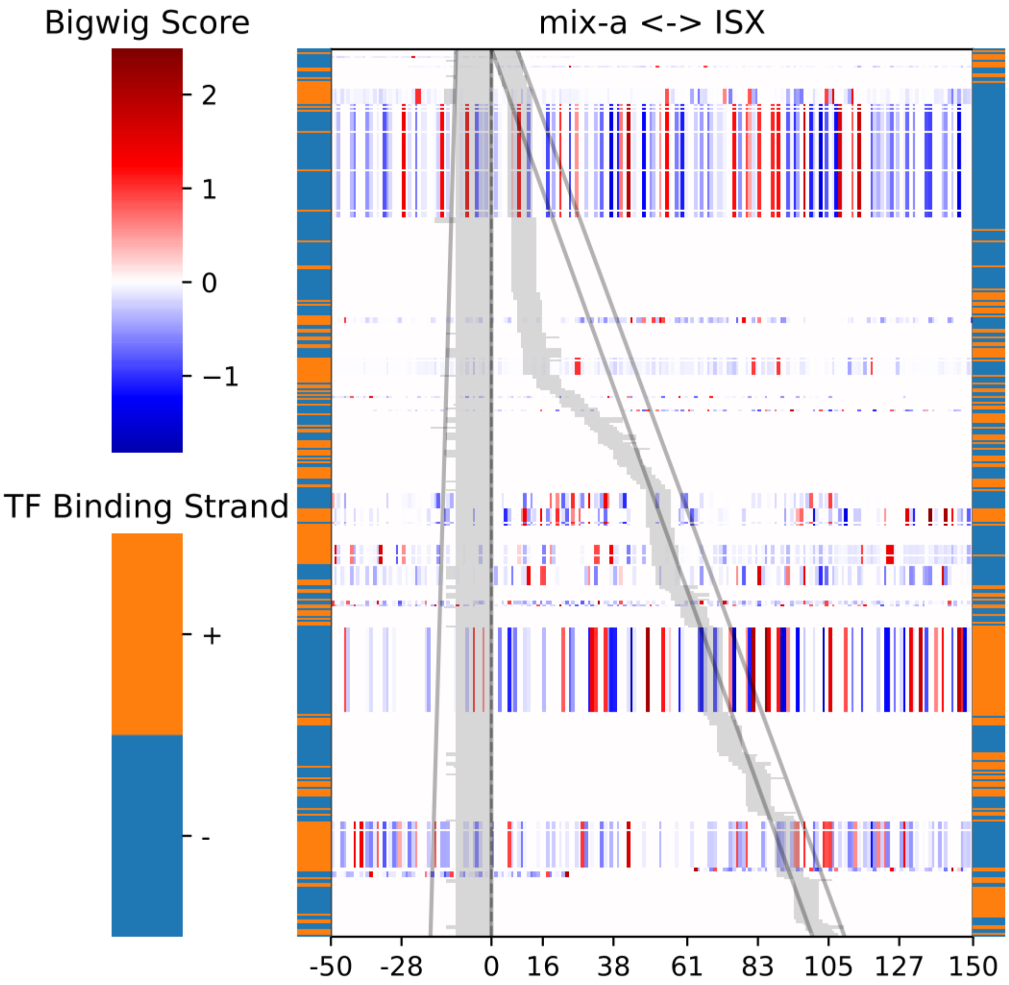

Bigwig Score
2
1
0
−1
TF Binding Strand
+
-
mix-a <-> ISX
-50
-28
0
16
38
61
83
105
127
150

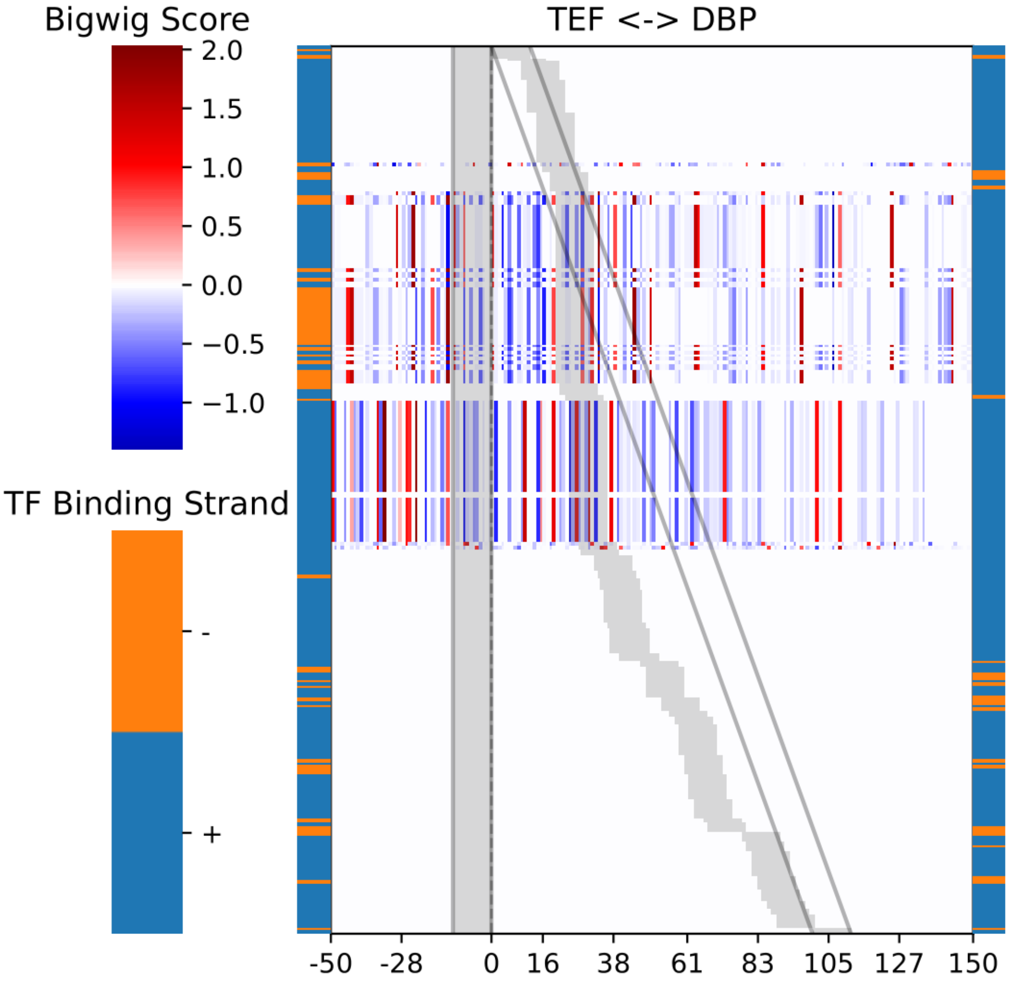

Bigwig Score
2.0
1.5
1.0
0.5
0.0
−0.5
−1.0
TF Binding Strand
-
+
TEF <-> DBP
-50
-28
0
16
38
61
83
105
127
150

Supplemental Information: Common TF pairs for Neuron user (merged)

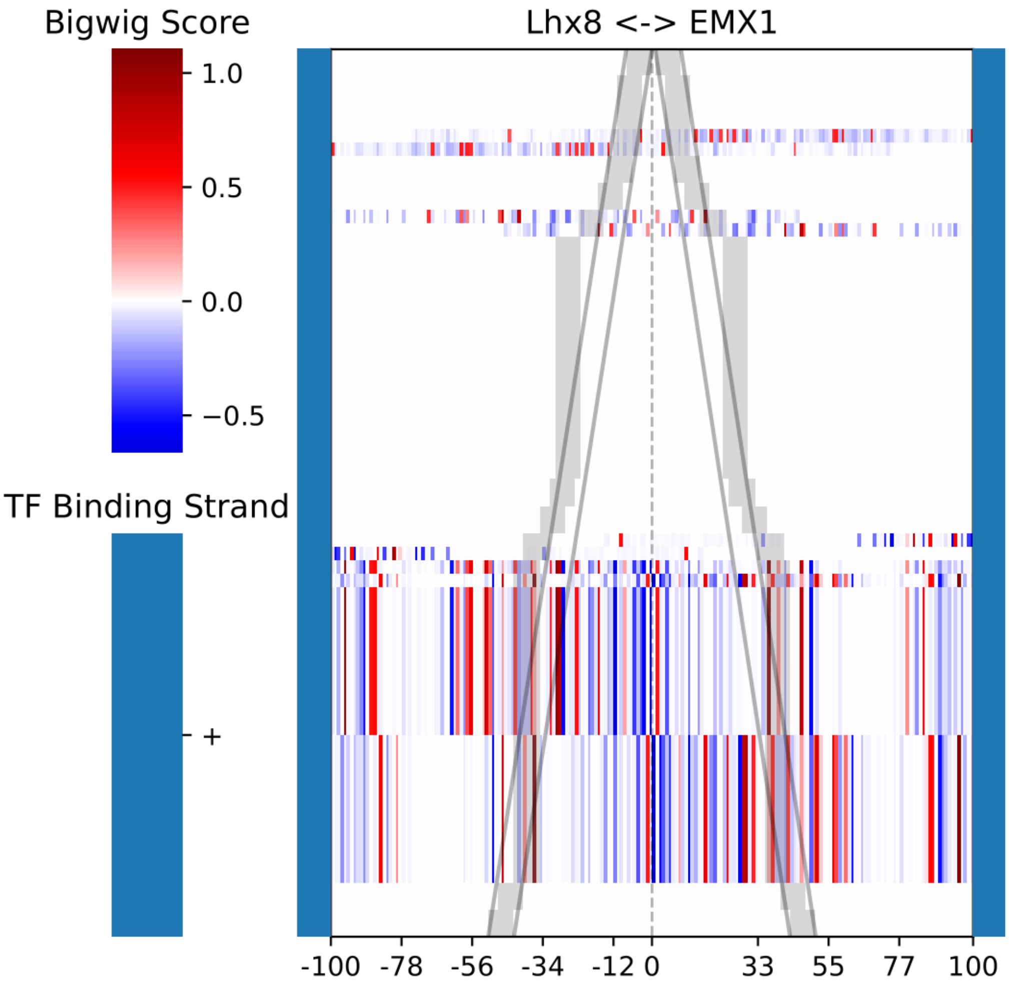

Bigwig Score
1.0
0.5
0.0
−0.5
TF Binding Strand
+
Lhx8 <-> EMX1
-100
-78
-56
-34
-12
0
33
55
77
100

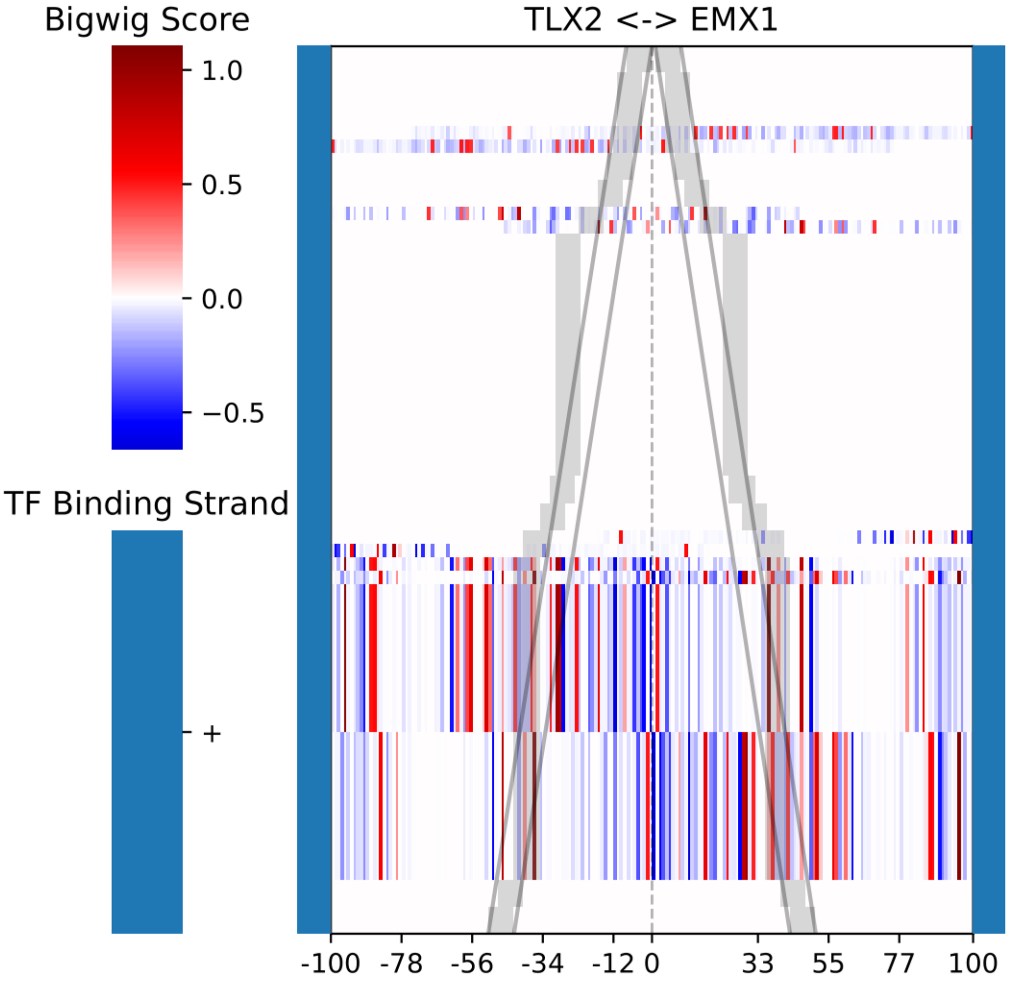

Bigwig Score
1.0
0.5
0.0
−0.5
TF Binding Strand
+
TLX2 <-> EMX1
-100
-78
-56
-34
-12
0
33
55
77
100

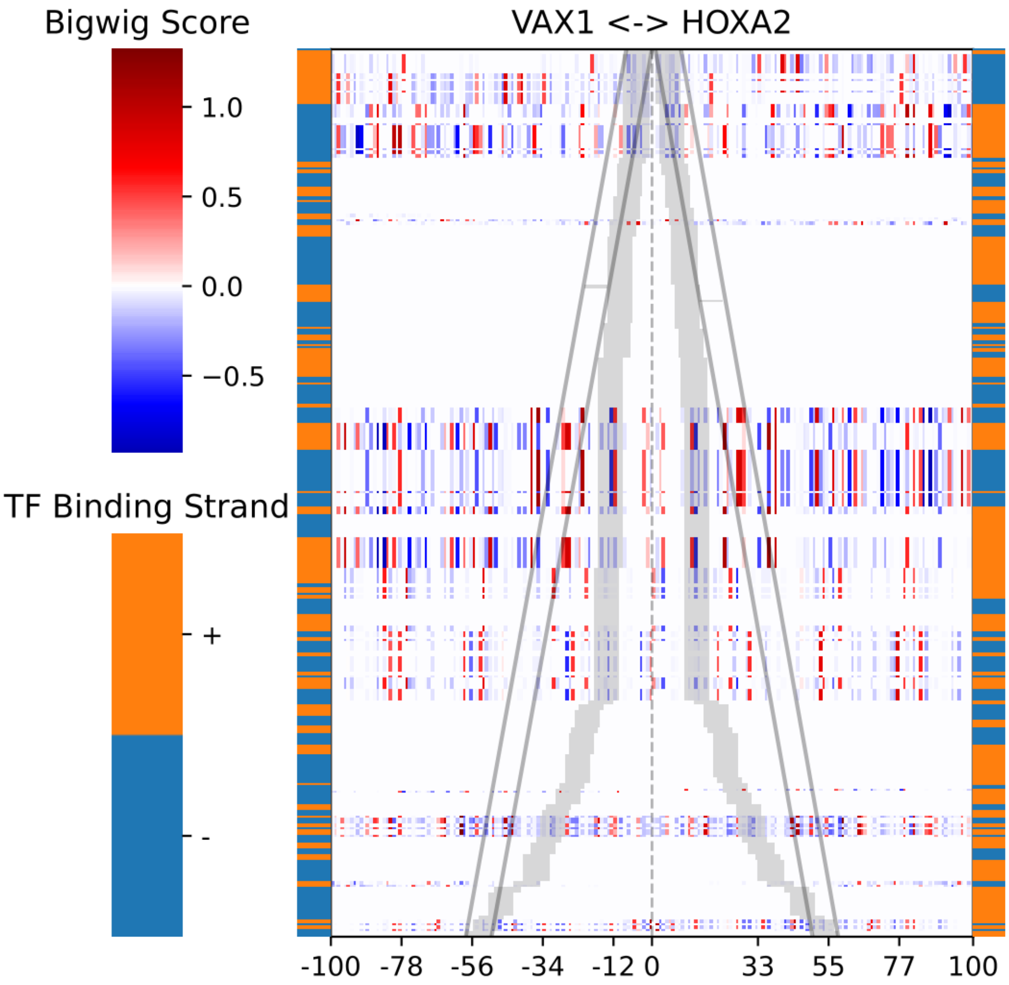

Bigwig Score
1.0
0.5
0.0
−0.5
TF Binding Strand
+
-
VAX1 <-> HOXA2
-100
-78
-56
-34
-12
0
33
55
77
100

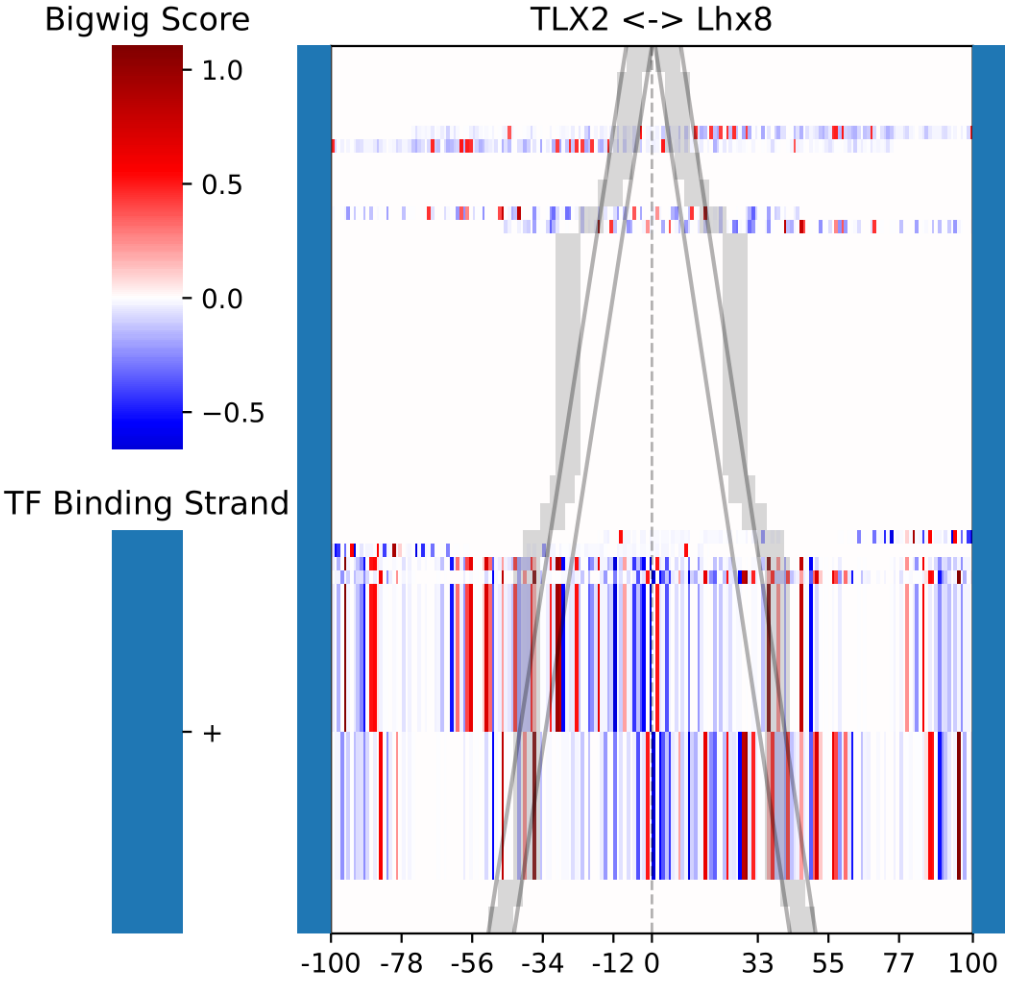

Bigwig Score
1.0
0.5
0.0
−0.5
TF Binding Strand
+
TLX2 <-> Lhx8
-100
-78
-56
-34
-12
0
33
55
77
100

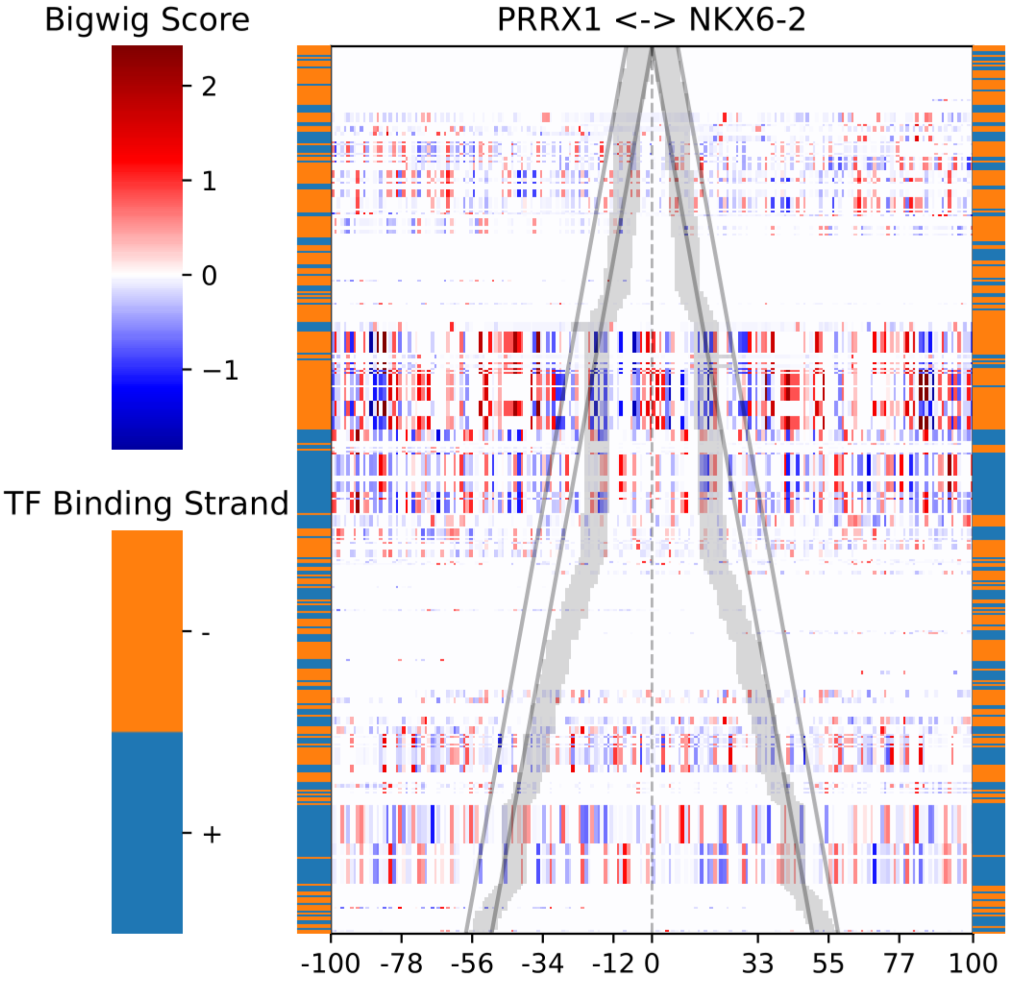

Bigwig Score
2
1
0
−1
TF Binding Strand
-
+
PRRX1 <-> NKX6-2
-100
-78
-56
-34
-12
0
33
55
77
100

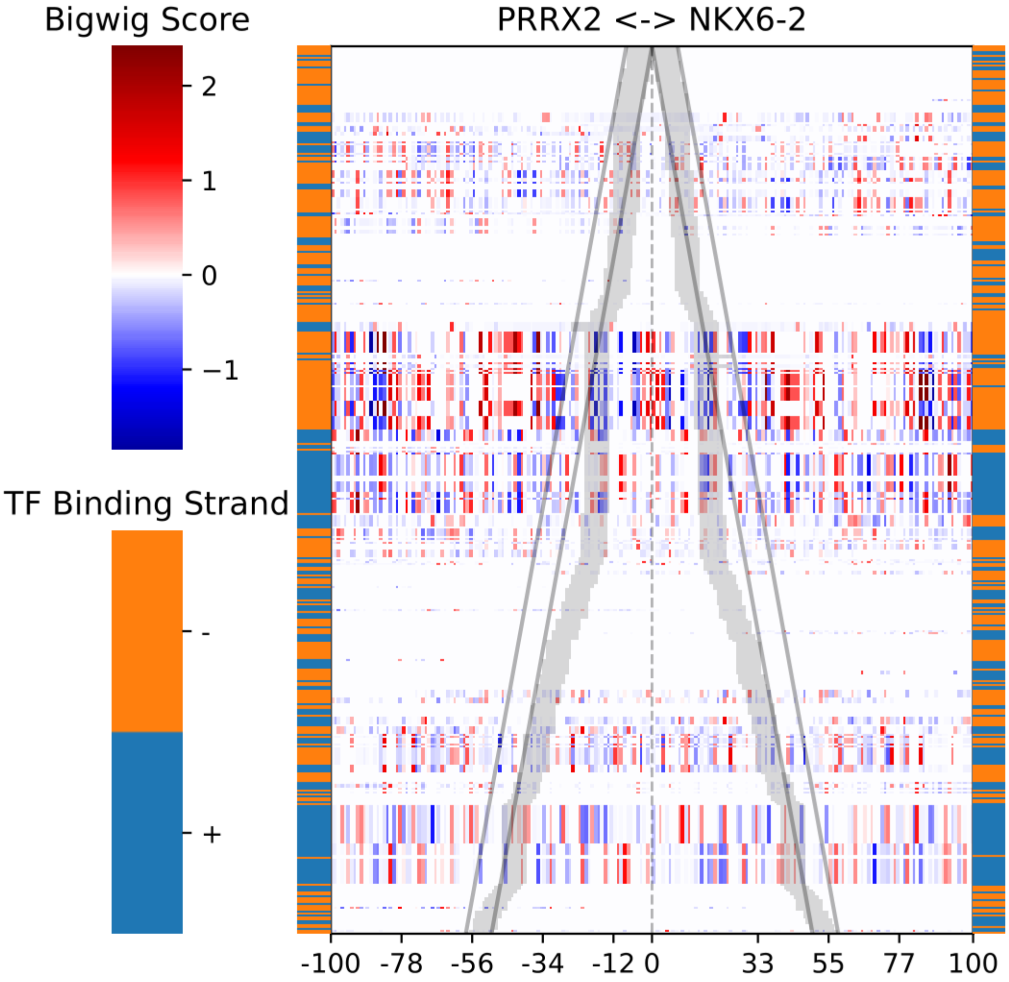

Bigwig Score
2
1
0
−1
TF Binding Strand
-
+
PRRX2 <-> NKX6-2
-100
-78
-56
-34
-12
0
33
55
77
100

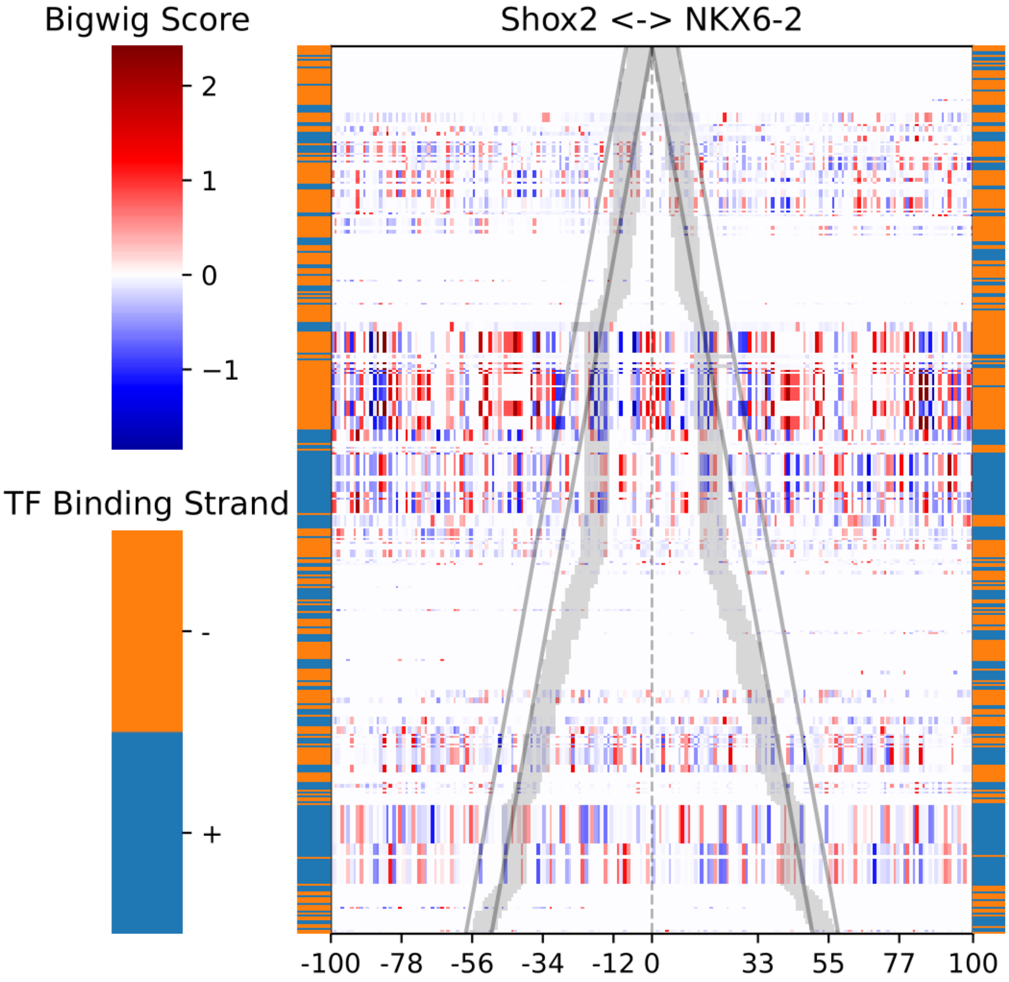

Bigwig Score
2
1
0
−1
TF Binding Strand
-
+
Shox2 <-> NKX6-2
-100
-78
-56
-34
-12
0
33
55
77
100

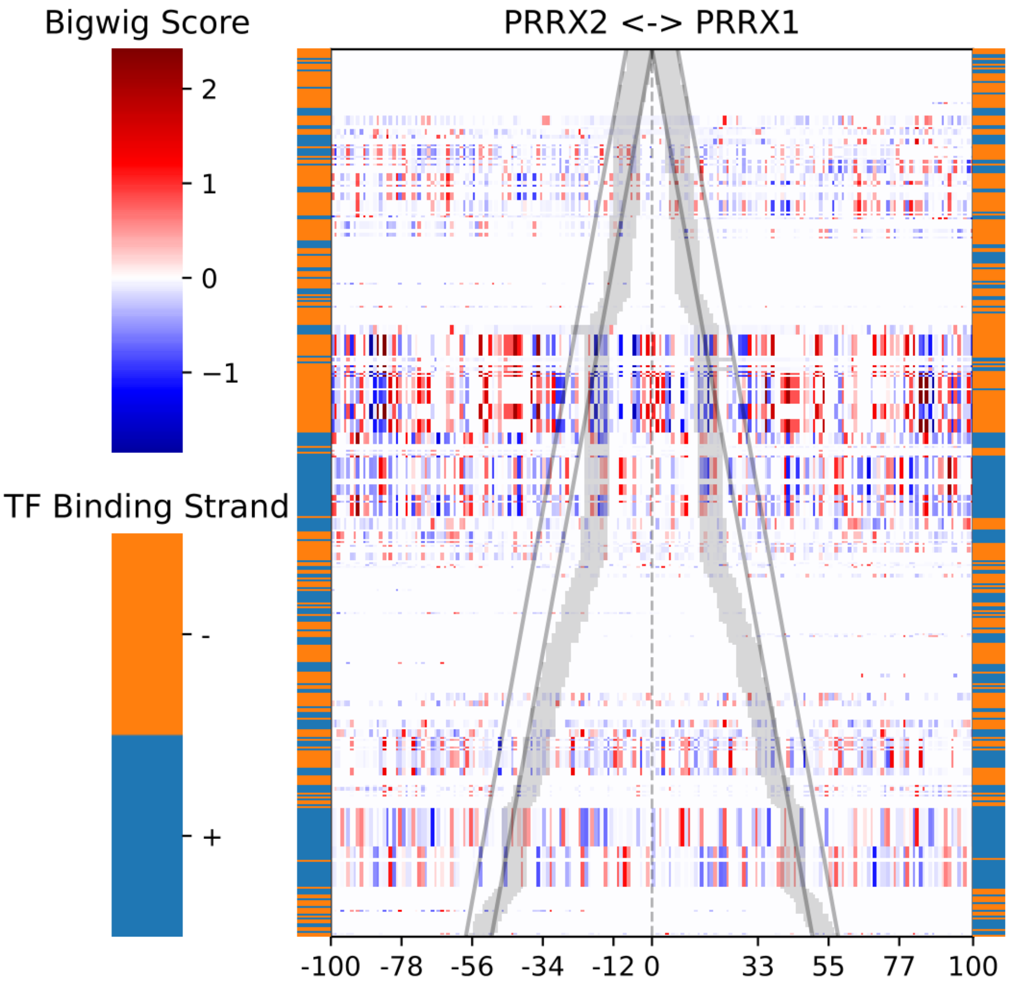

Bigwig Score
2
1
0
−1
TF Binding Strand
-
+
PRRX2 <-> PRRX1
-100
-78
-56
-34
-12
0
33
55
77
100

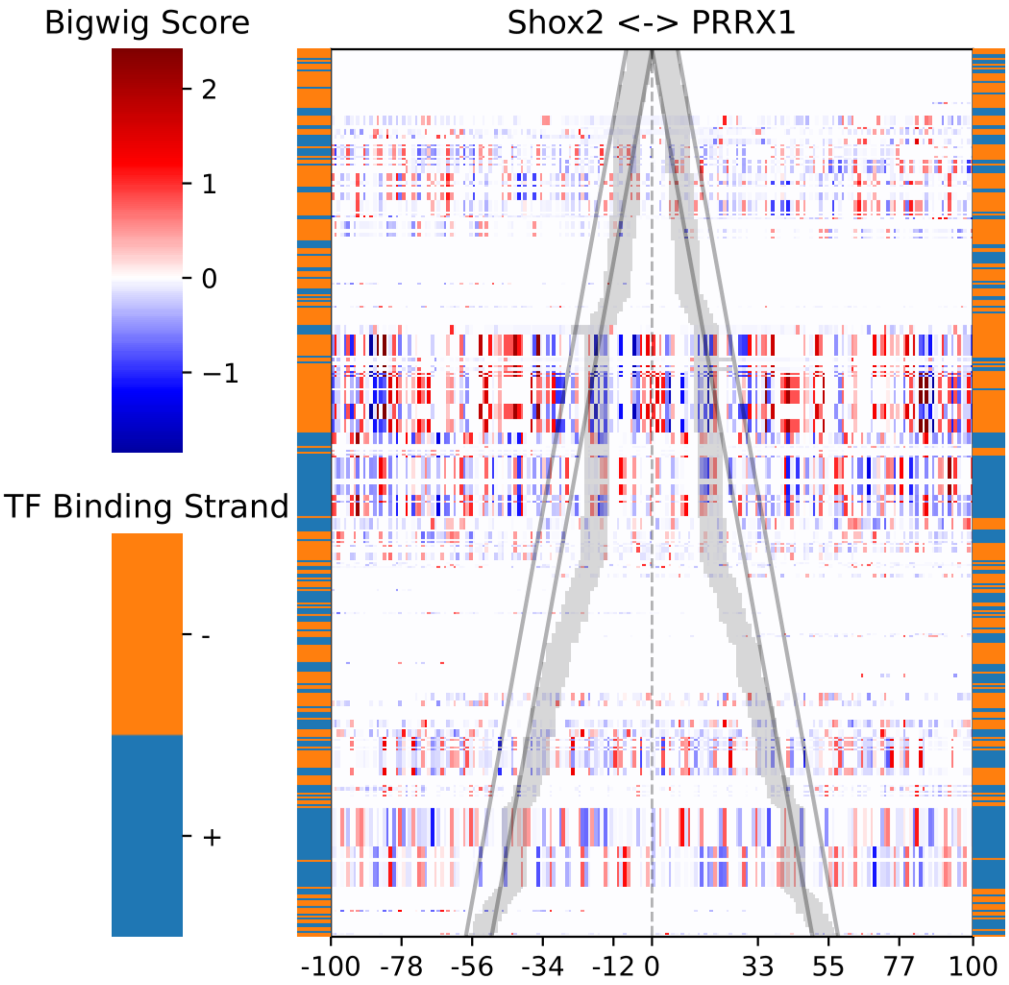

Bigwig Score
2
1
0
−1
TF Binding Strand
-
+
Shox2 <-> PRRX1
-100
-78
-56
-34
-12
0
33
55
77
100

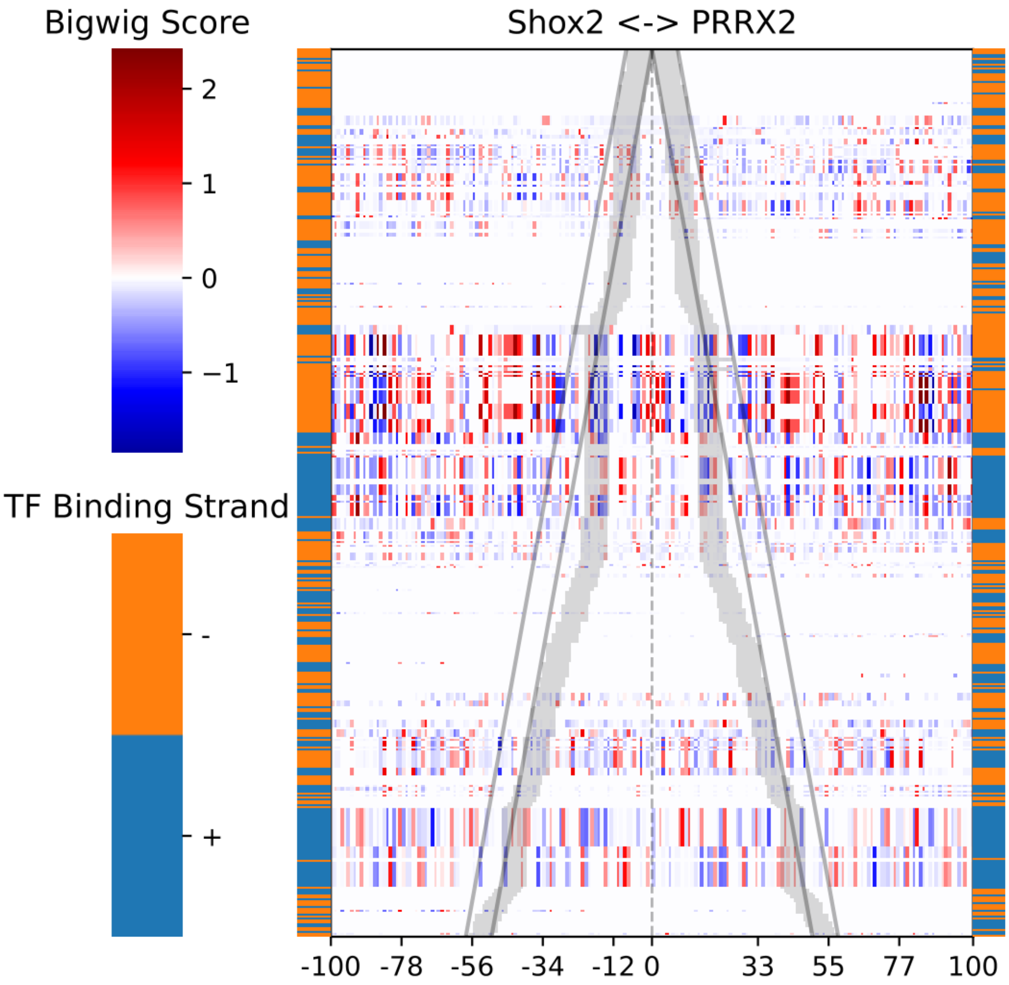
Bigwig Score
2
1
0
−1
TF Binding Strand
-
+
Shox2 <-> PRRX2
-100
-78
-56
-34
-12
0
33
55
77
100

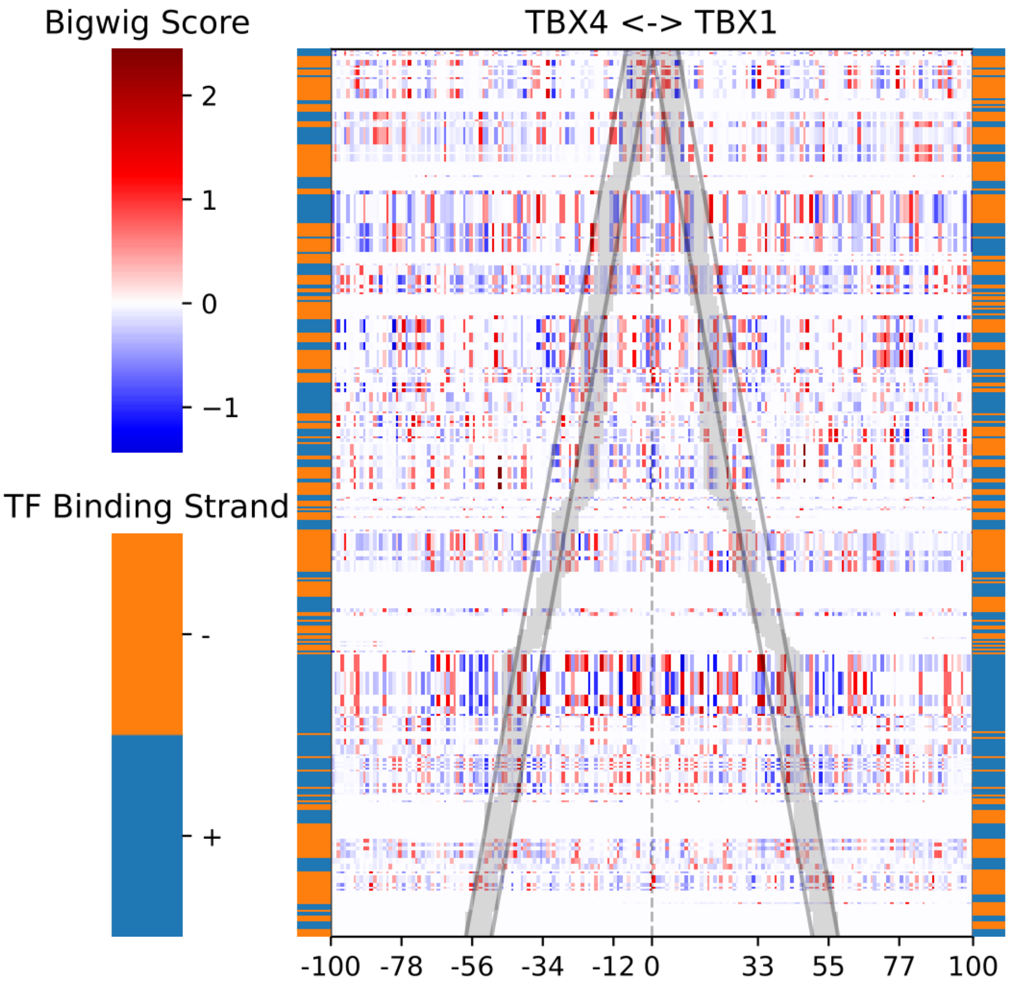

Bigwig Score
2
1
0
−1
TF Binding Strand
-
+
TBX4 <-> TBX1
-100
-78
-56
-34
-12
0
33
55
77
100

Supplemental Information: Common TF pairs for Neuron non-user (merged)

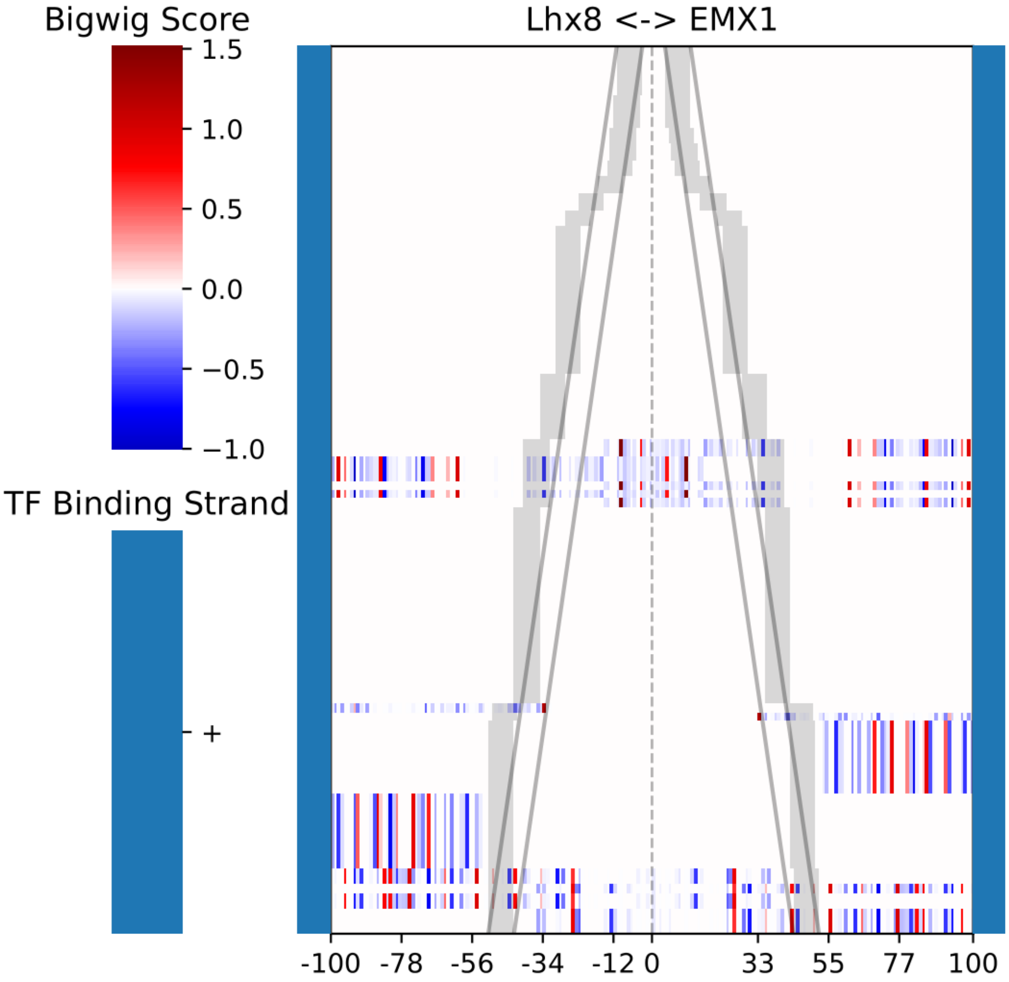
Bigwig Score
1.5
1.0
0.5
0.0
−0.5
−1.0
TF Binding Strand
+
Lhx8 <-> EMX1
-100
-78
-56
-34
-12
0
33
55
77
100

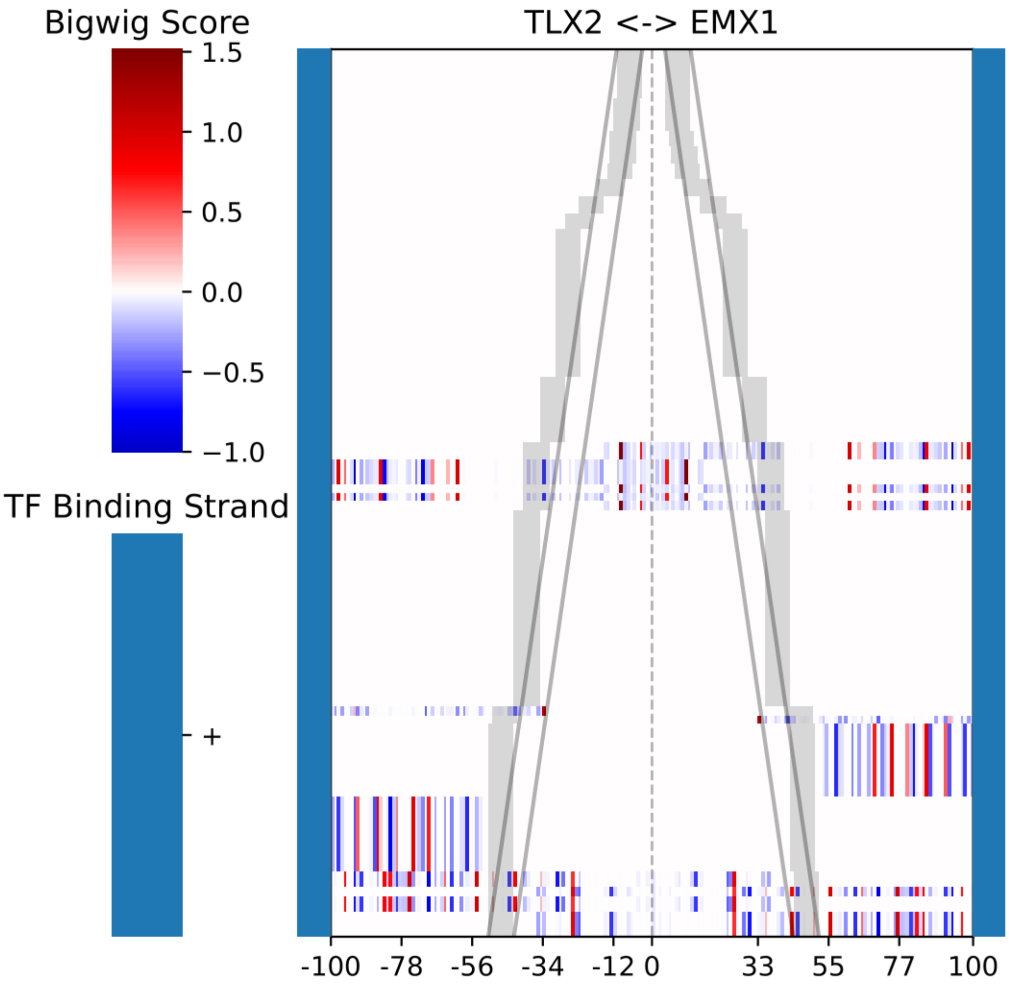
Bigwig Score
1.5
1.0
0.5
0.0
−0.5
−1.0
TF Binding Strand
+
TLX2 <-> EMX1
-100
-78
-56
-34
-12
0
33
55
77
100

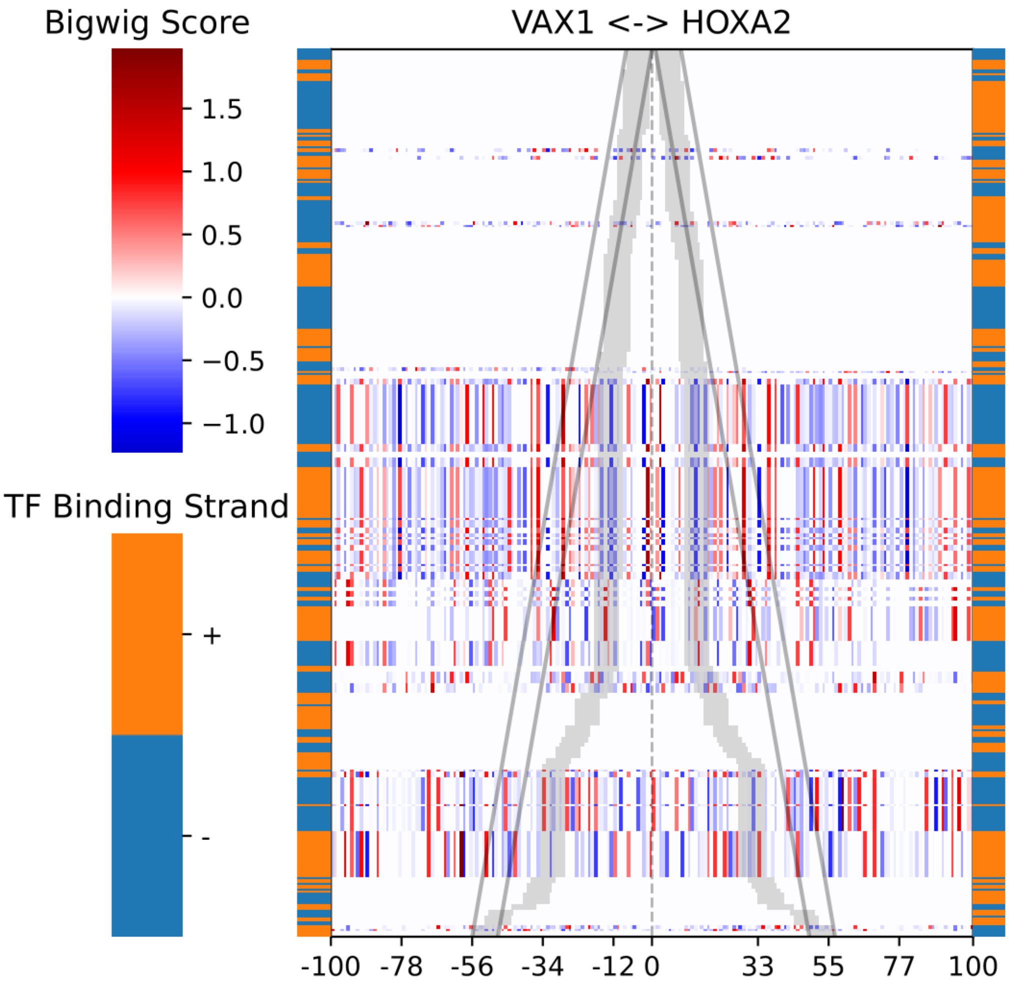

Bigwig Score
1.5
1.0
0.5
0.0
−0.5
−1.0
TF Binding Strand
+
-
VAX1 <-> HOXA2
-100
-78
-56
-34
-12
0
33
55
77
100

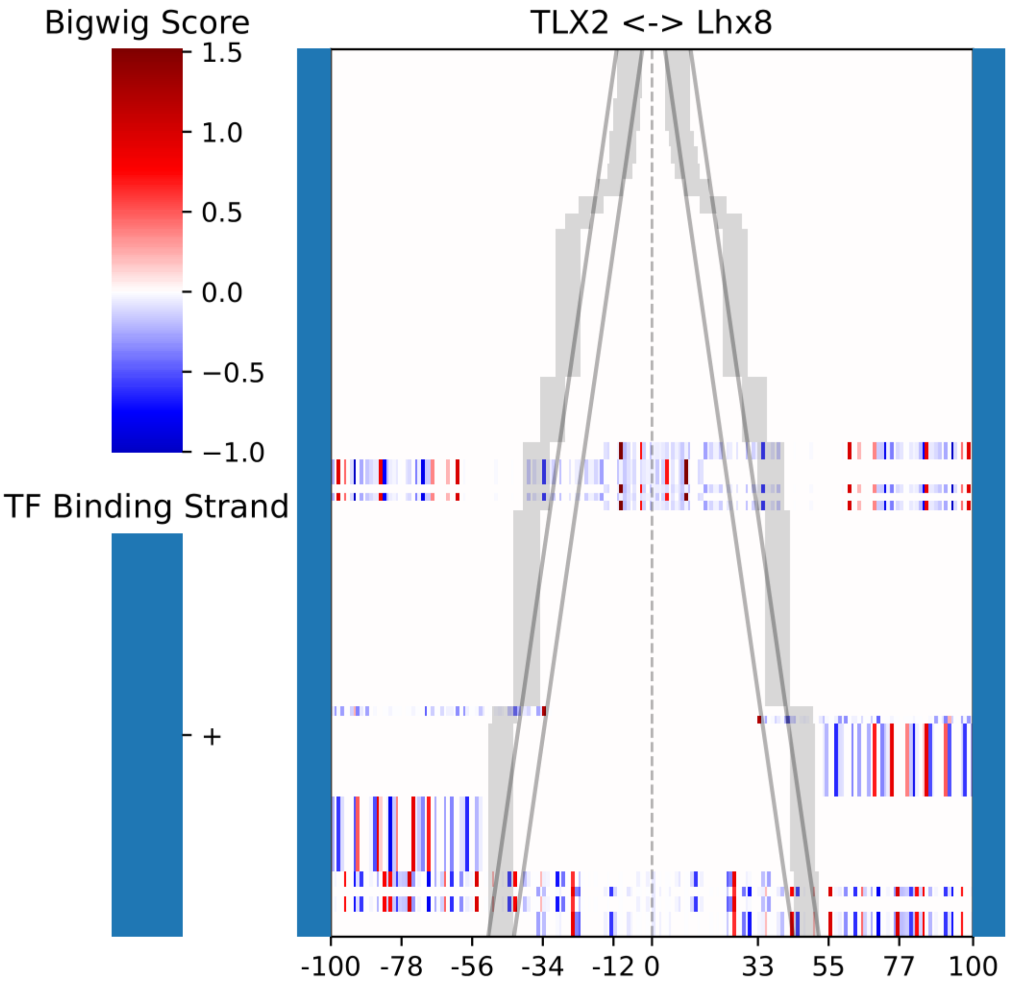
Bigwig Score
1.5
1.0
0.5
0.0
−0.5
−1.0
TF Binding Strand
+
TLX2 <-> Lhx8
-100
-78
-56
-34
-12
0
33
55
77
100

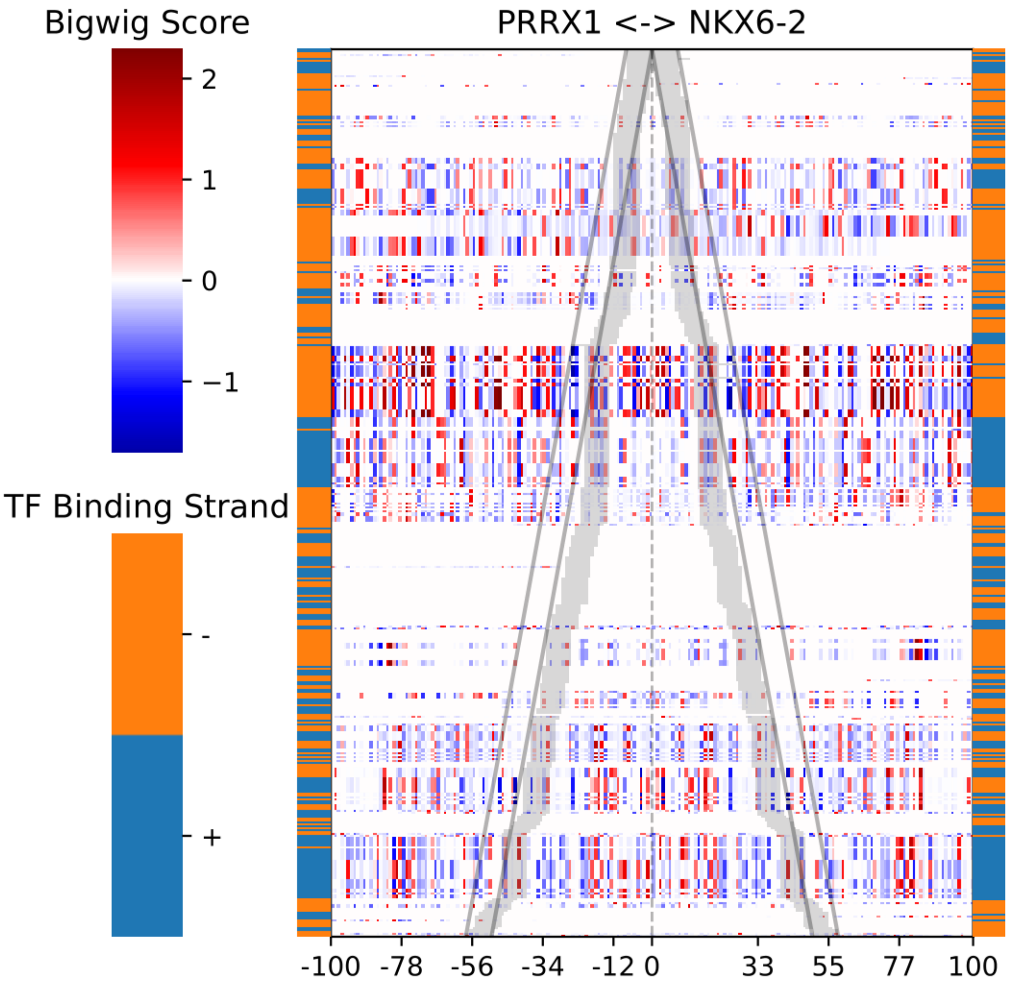

Bigwig Score
2
1
0
−1
TF Binding Strand
-
+
PRRX1 <-> NKX6-2
-100
-78
-56
-34
-12
0
33
55
77
100

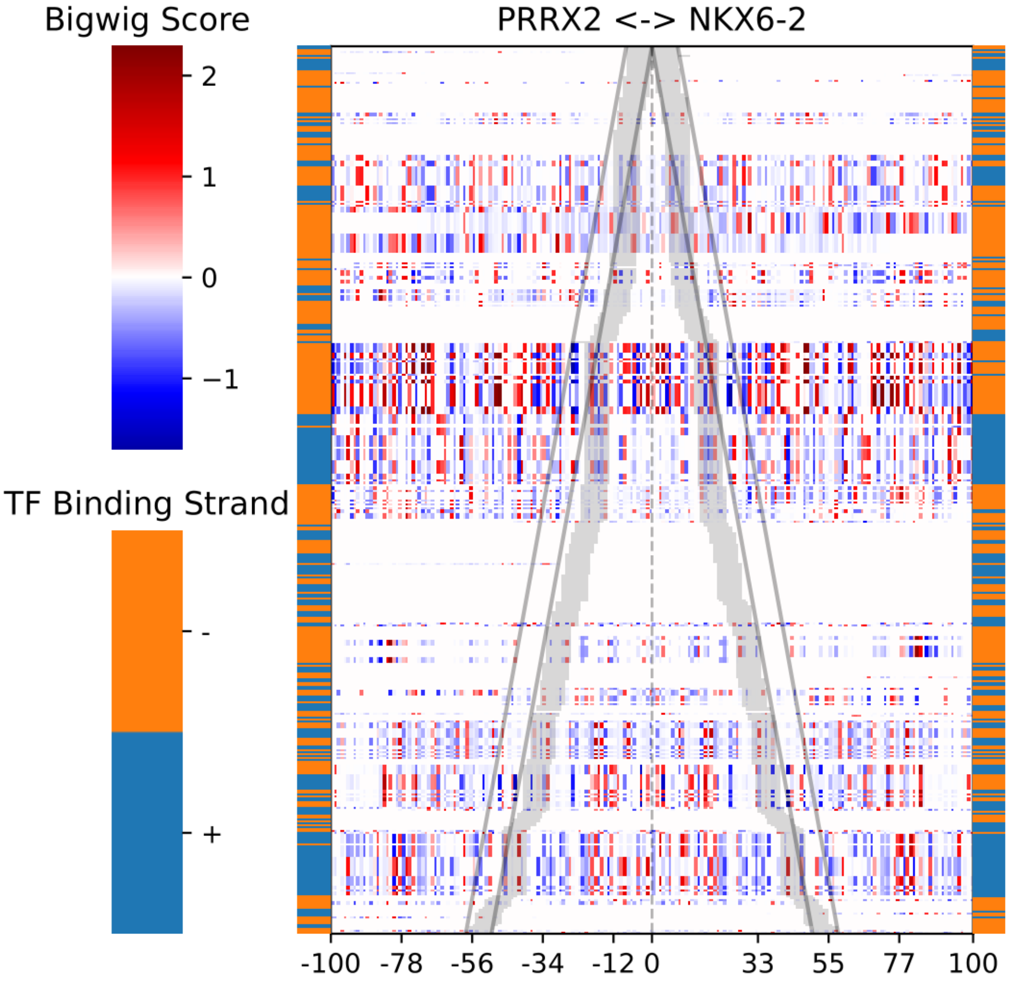
Bigwig Score
2
1
0
−1
TF Binding Strand
-
+
PRRX2 <-> NKX6-2
-100
-78
-56
-34
-12
0
33
55
77
100

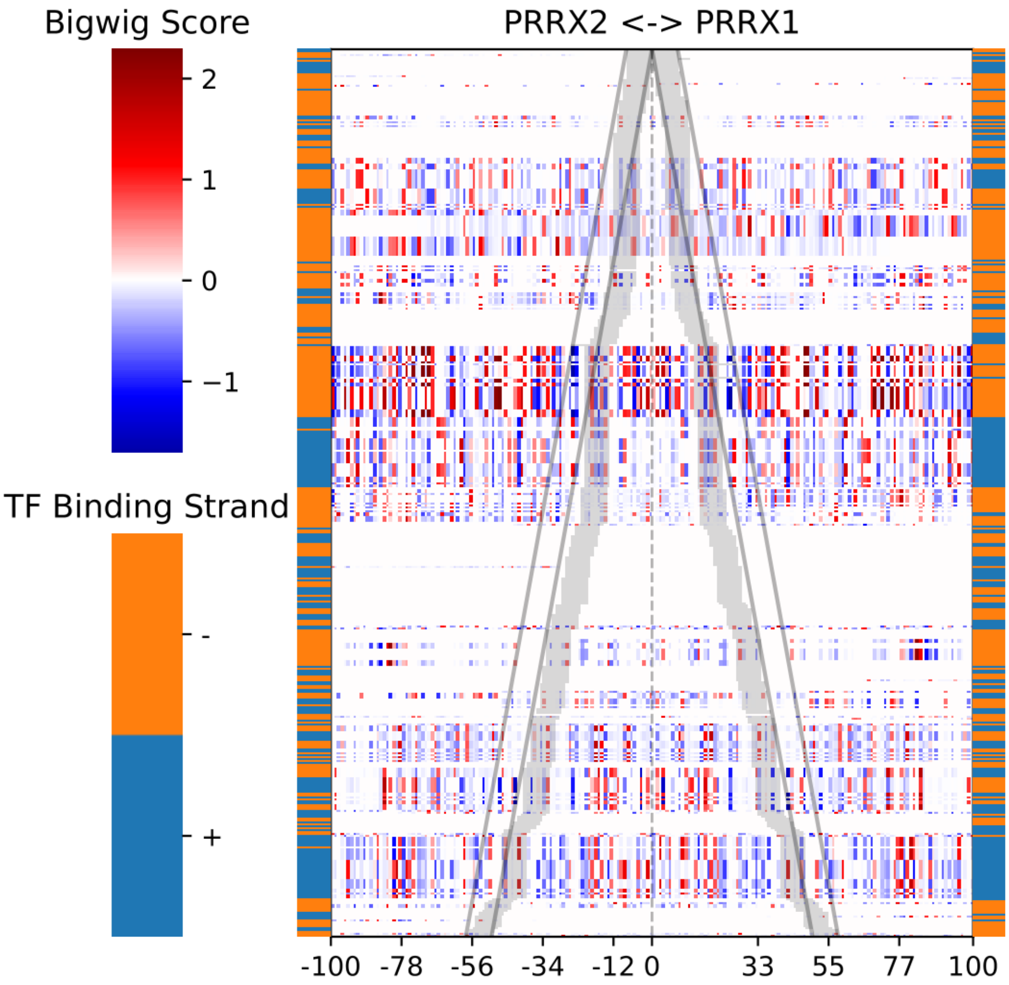
Bigwig Score
2
1
0
−1
TF Binding Strand
-
+
PRRX2 <-> PRRX1
-100
-78
-56
-34
-12
0
33
55
77
100

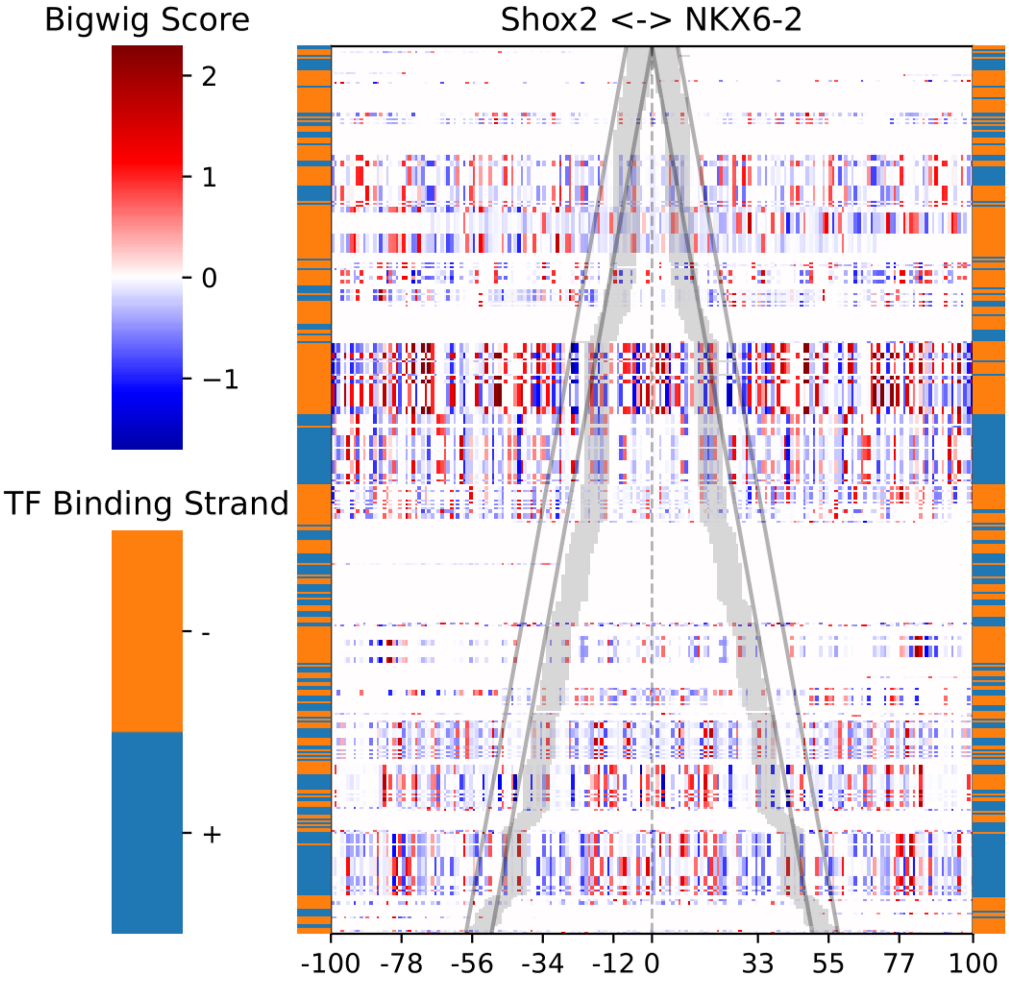

Bigwig Score
2
1
0
−1
TF Binding Strand
-
+
Shox2 <-> NKX6-2
-100
-78
-56
-34
-12
0
33
55
77
100

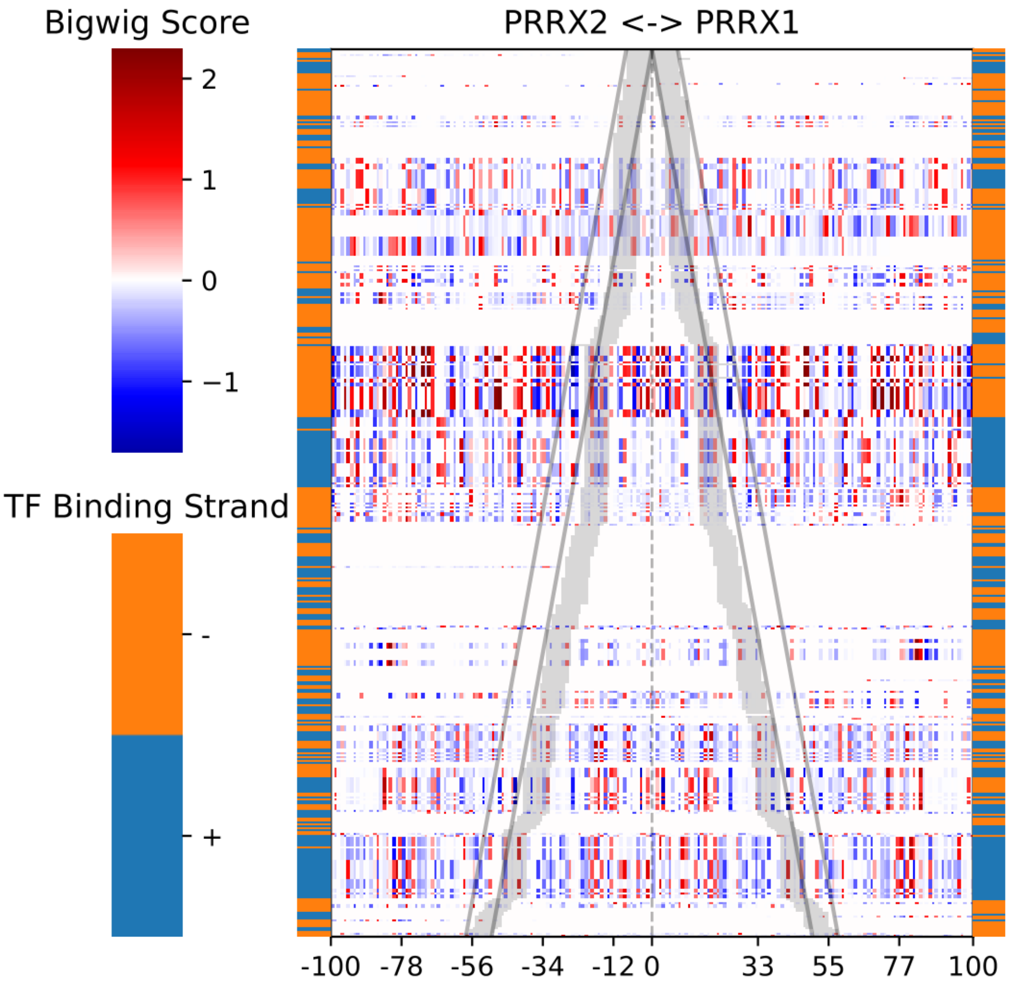
Bigwig Score
2
1
0
−1
TF Binding Strand
-
+
PRRX2 <-> PRRX1
-100
-78
-56
-34
-12
0
33
55
77
100

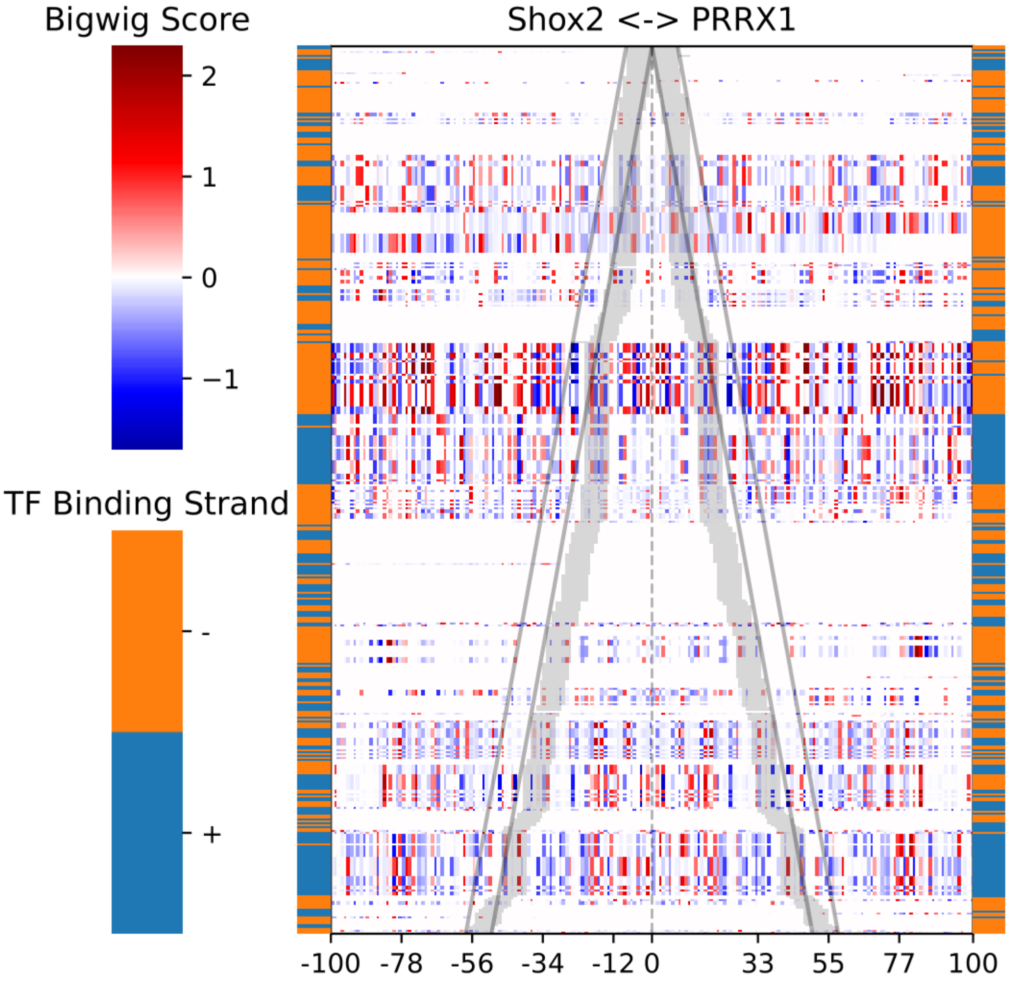

Bigwig Score
2
1
0
−1
TF Binding Strand
-
+
Shox2 <-> PRRX1
-100
-78
-56
-34
-12
0
33
55
77
100

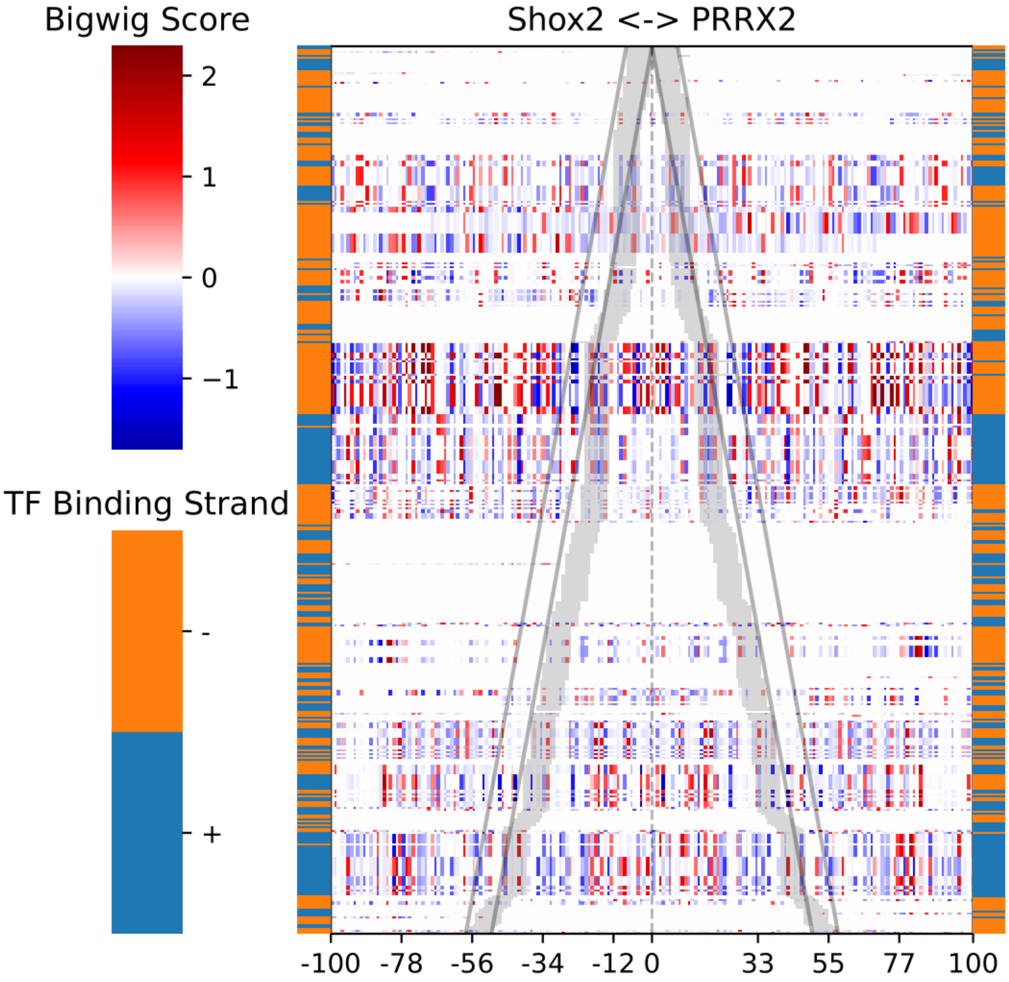

Bigwig Score
2
1
0
−1
TF Binding Strand
-
+
Shox2 <-> PRRX2
-100
-78
-56
-34
-12
0
33
55
77
100

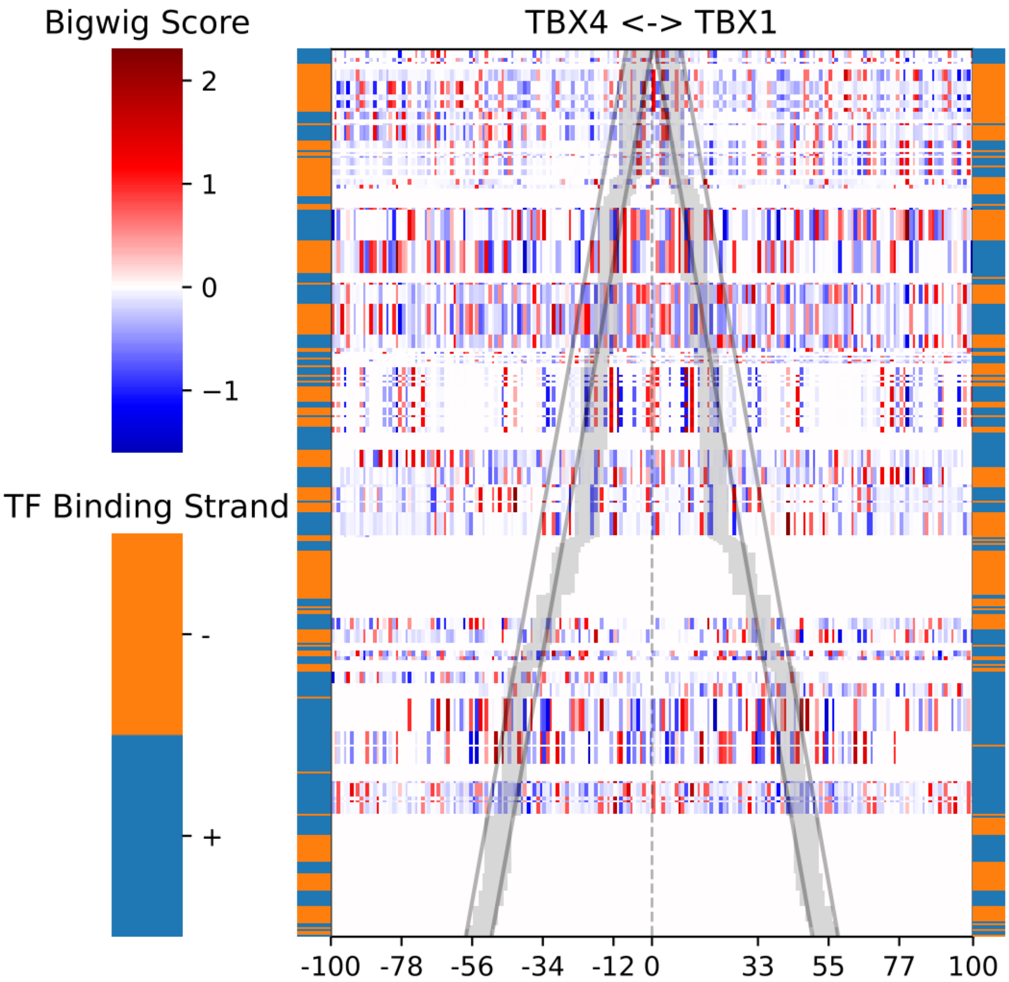

Bigwig Score
2
1
0
−1
TF Binding Strand
-
+
TBX4 <-> TBX1
-100
-78
-56
-34
-12
0
33
55
77
100

Supplemental Information: Common TF pairs for Glia user (merged)

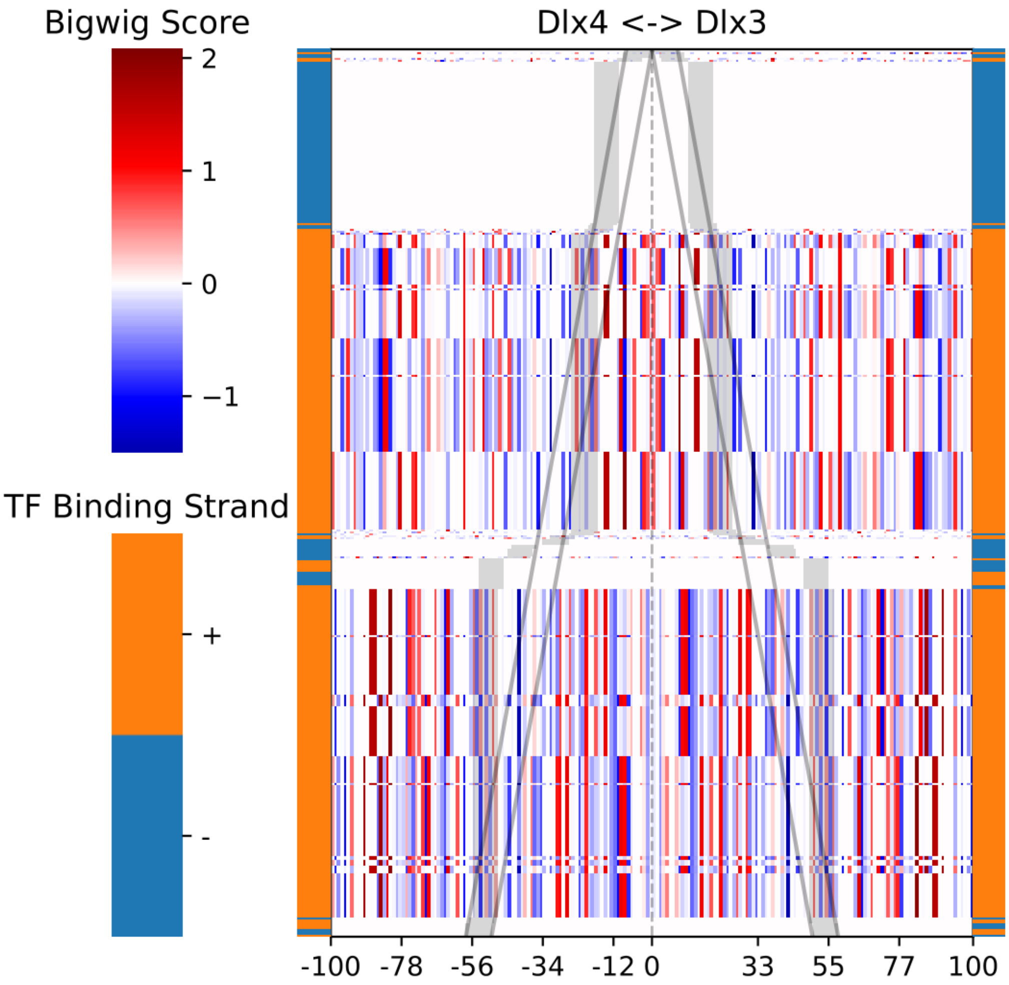
Bigwig Score
2
1
0
−1
TF Binding Strand
+
-
Dlx4 <-> Dlx3
-100
-78
-56
-34
-12
0
33
55
77
100

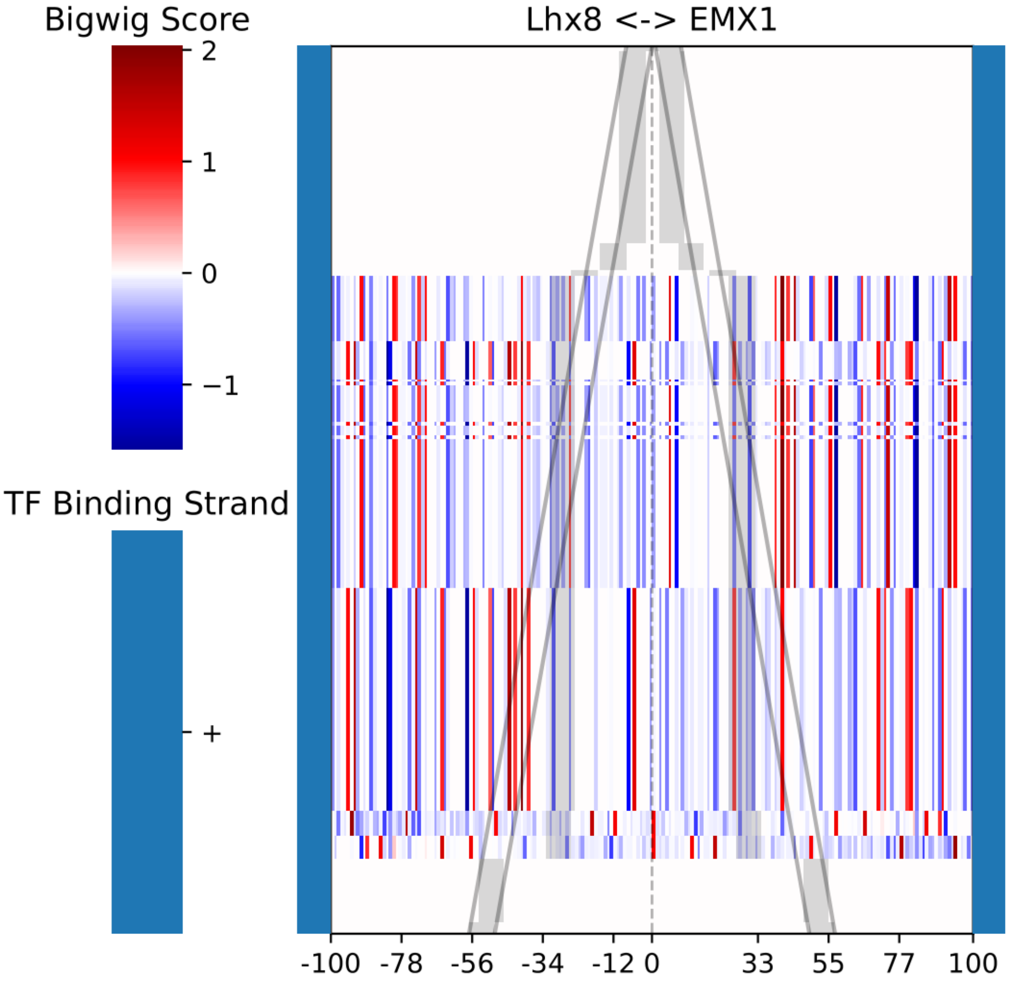

Bigwig Score
2
1
0
−1
TF Binding Strand
+
Lhx8 <-> EMX1
-100
-78
-56
-34
-12
0
33
55
77
100

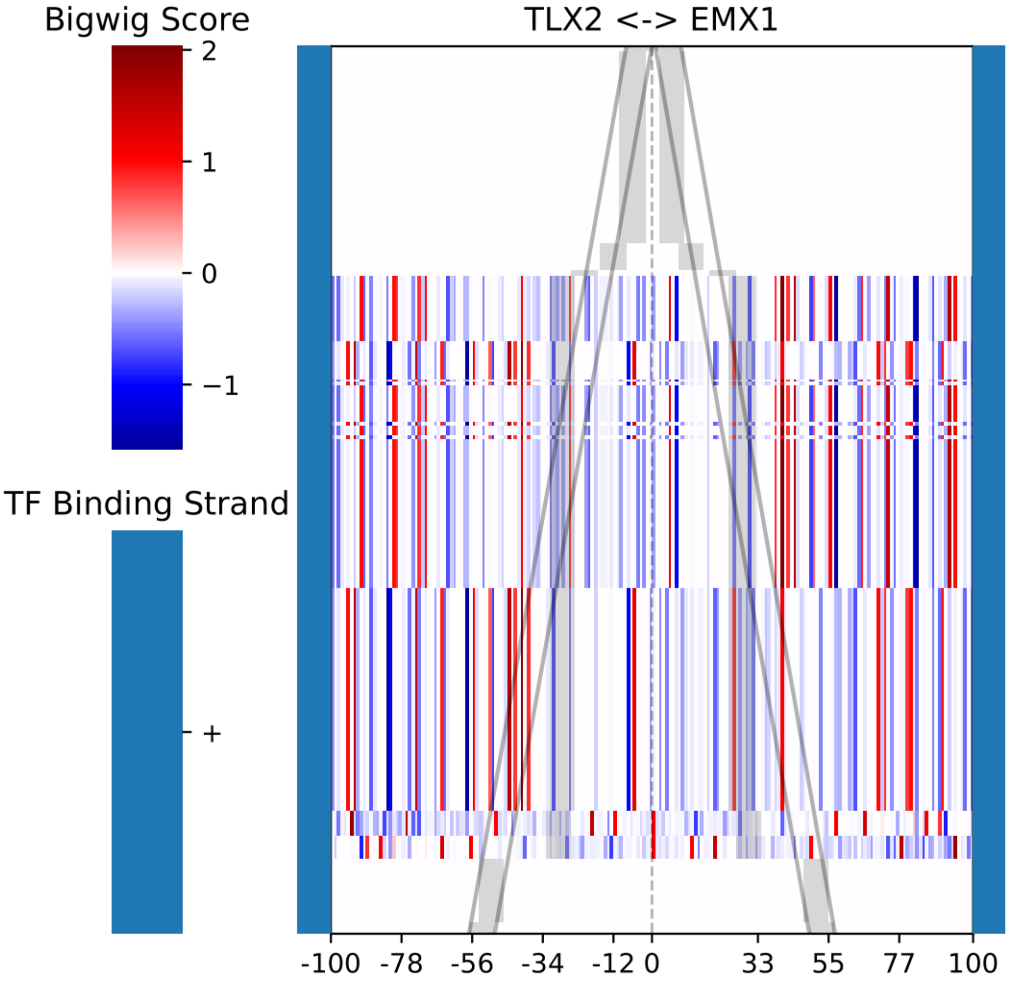

Bigwig Score
2
1
0
−1
TF Binding Strand
+
TLX2 <-> EMX1
-100
-78
-56
-34
-12
0
33
55
77
100

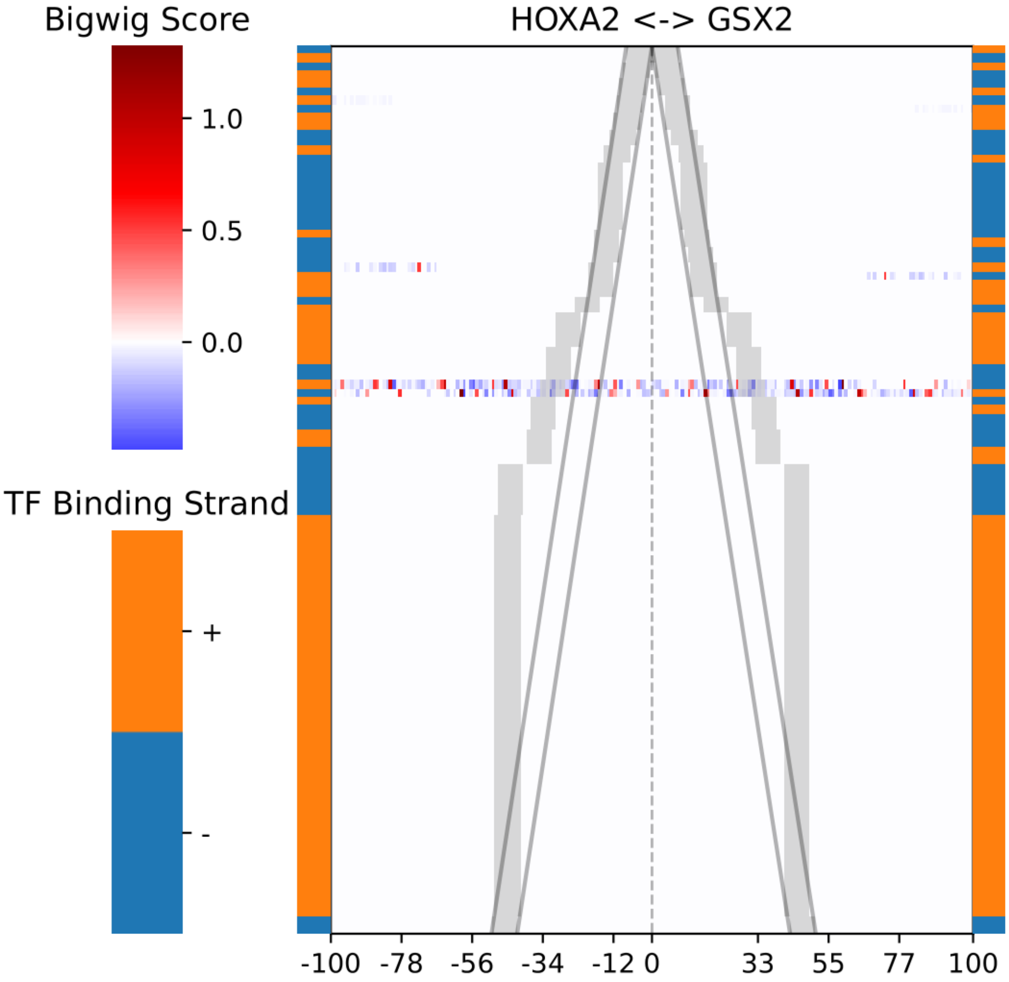

Bigwig Score
1.0
0.5
0.0
TF Binding Strand
+
-
HOXA2 <-> GSX2
-100
-78
-56
-34
-12
0
33
55
77
100

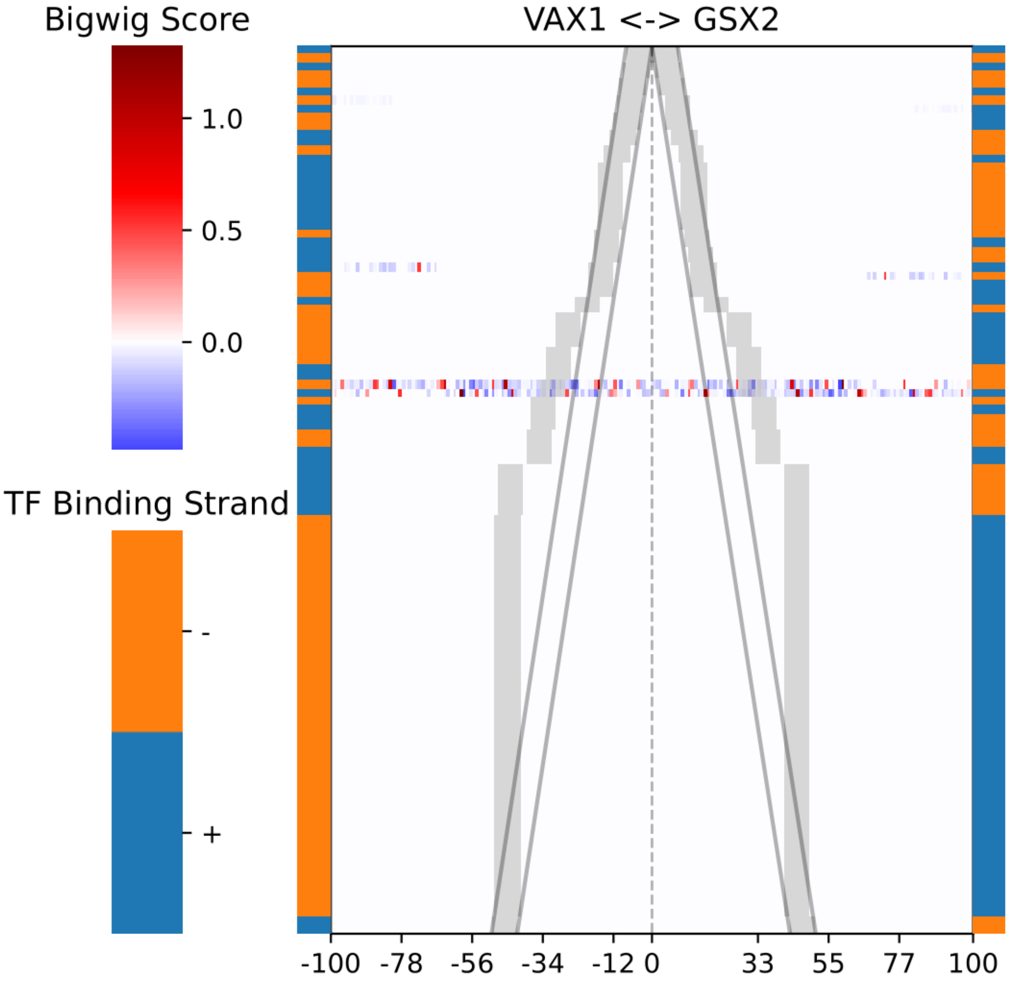
Bigwig Score
1.0
0.5
0.0
TF Binding Strand
-
+
VAX1 <-> GSX2
-100
-78
-56
-34
-12
0
33
55
77
100

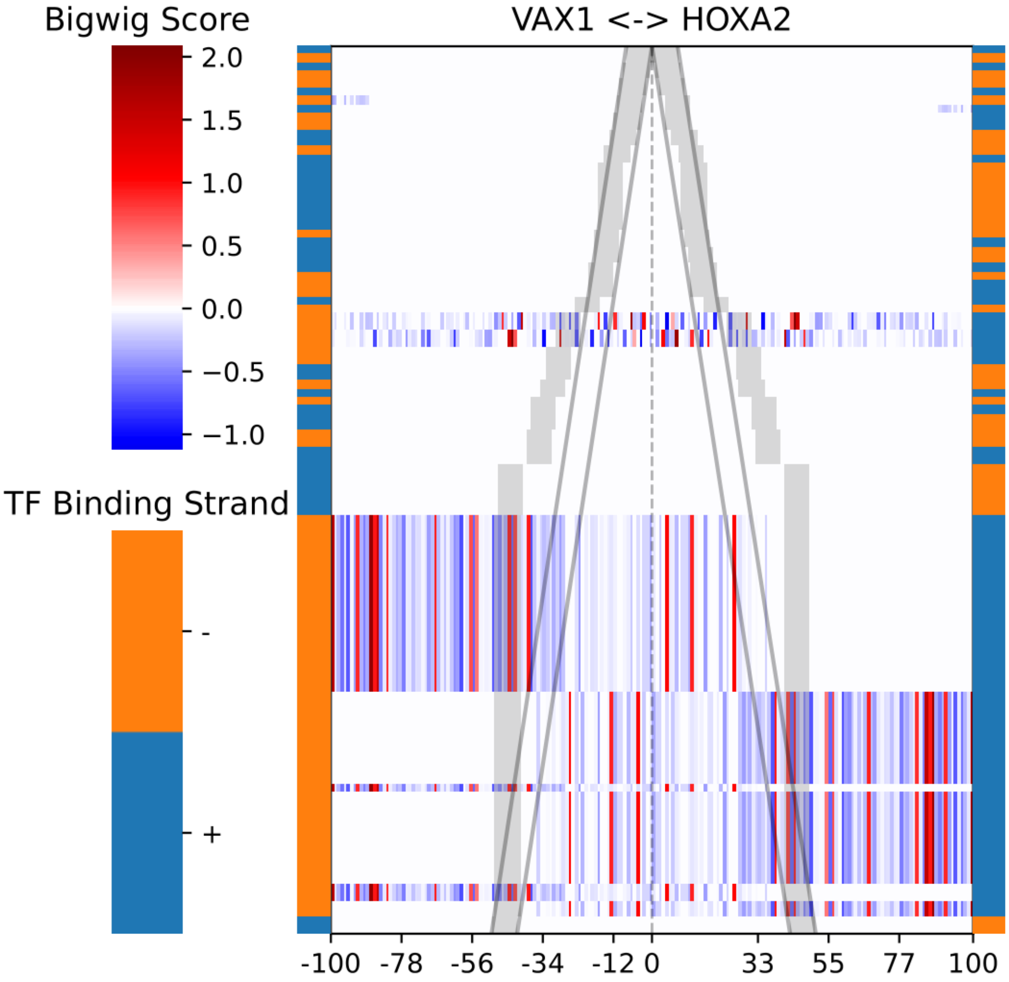
Bigwig Score
2.0
1.5
1.0
0.5
0.0
−0.5
−1.0
TF Binding Strand
-
+
VAX1 <-> HOXA2
-100
-78
-56
-34
-12
0
33
55
77
100

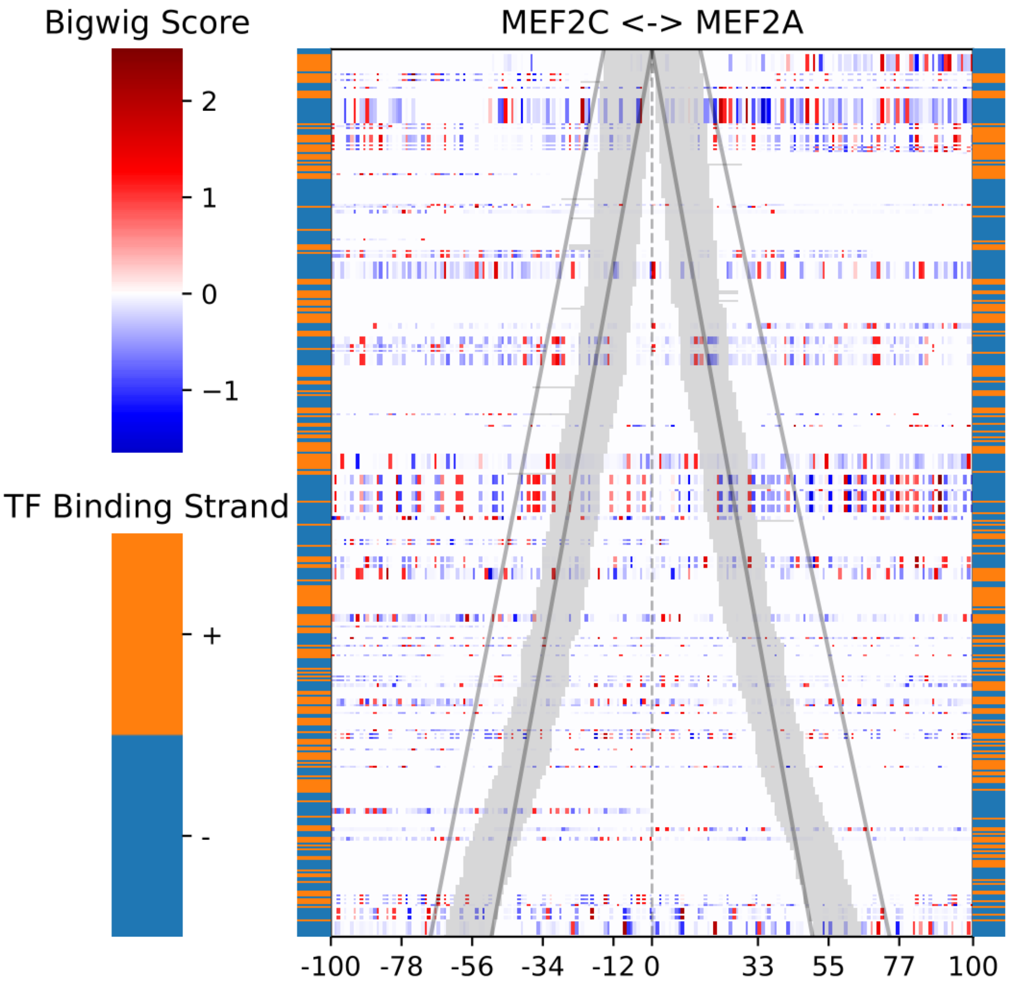

Bigwig Score
2
1
0
−1
TF Binding Strand
+
-
MEF2C <-> MEF2A
-100
-78
-56
-34
-12
0
33
55
77
100

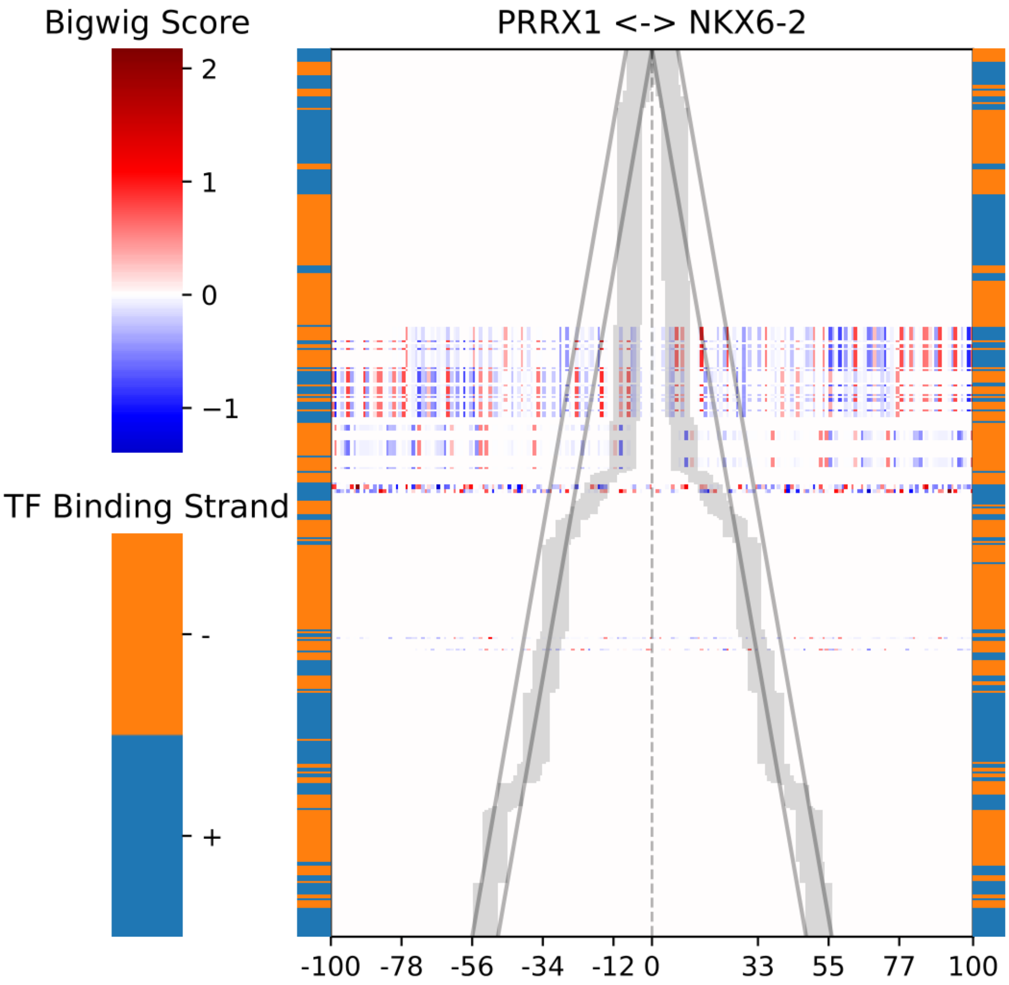
Bigwig Score
2
1
0
−1
TF Binding Strand
-
+
PRRX1 <-> NKX6-2
-100
-78
-56
-34
-12
0
33
55
77
100

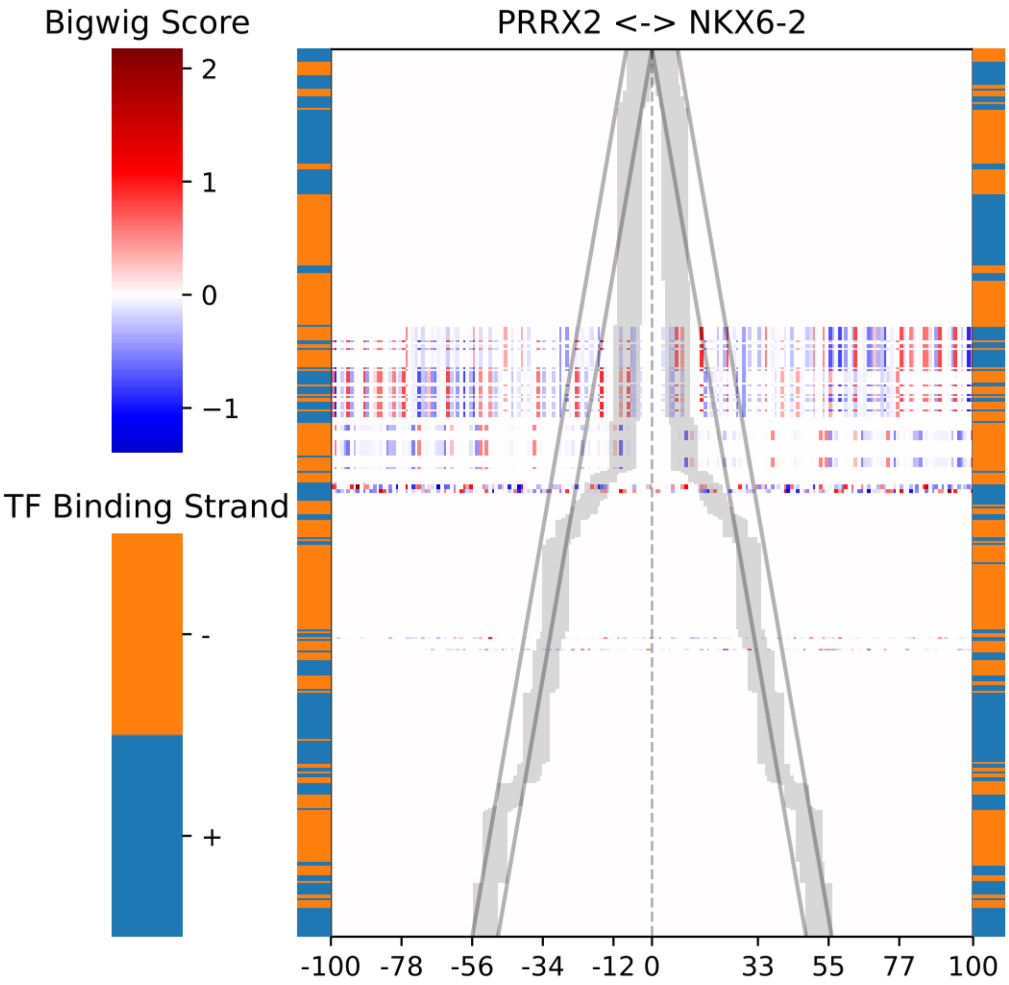

Bigwig Score
2
1
0
−1
TF Binding Strand
-
+
PRRX2 <-> NKX6-2
-100
-78
-56
-34
-12
0
33
55
77
100

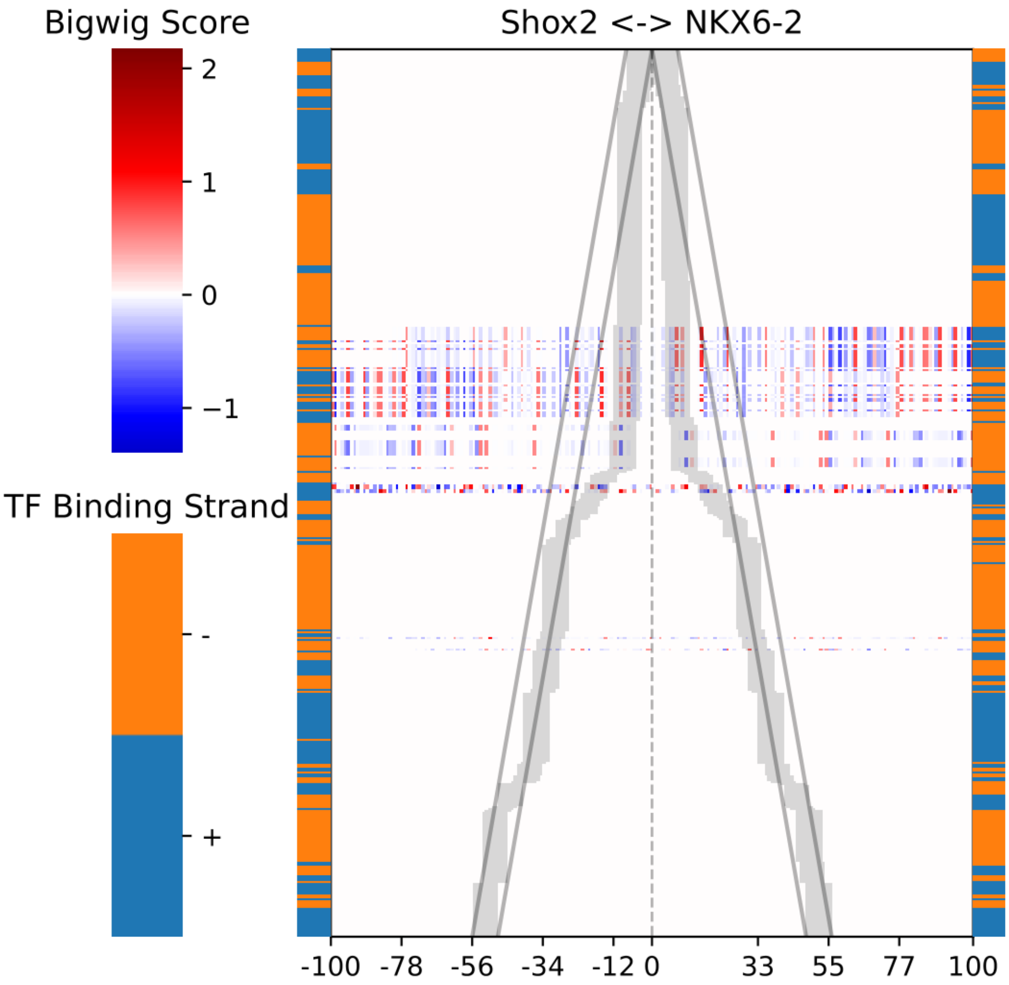

Bigwig Score
2
1
0
−1
TF Binding Strand
-
+
Shox2 <-> NKX6-2
-100
-78
-56
-34
-12
0
33
55
77
100

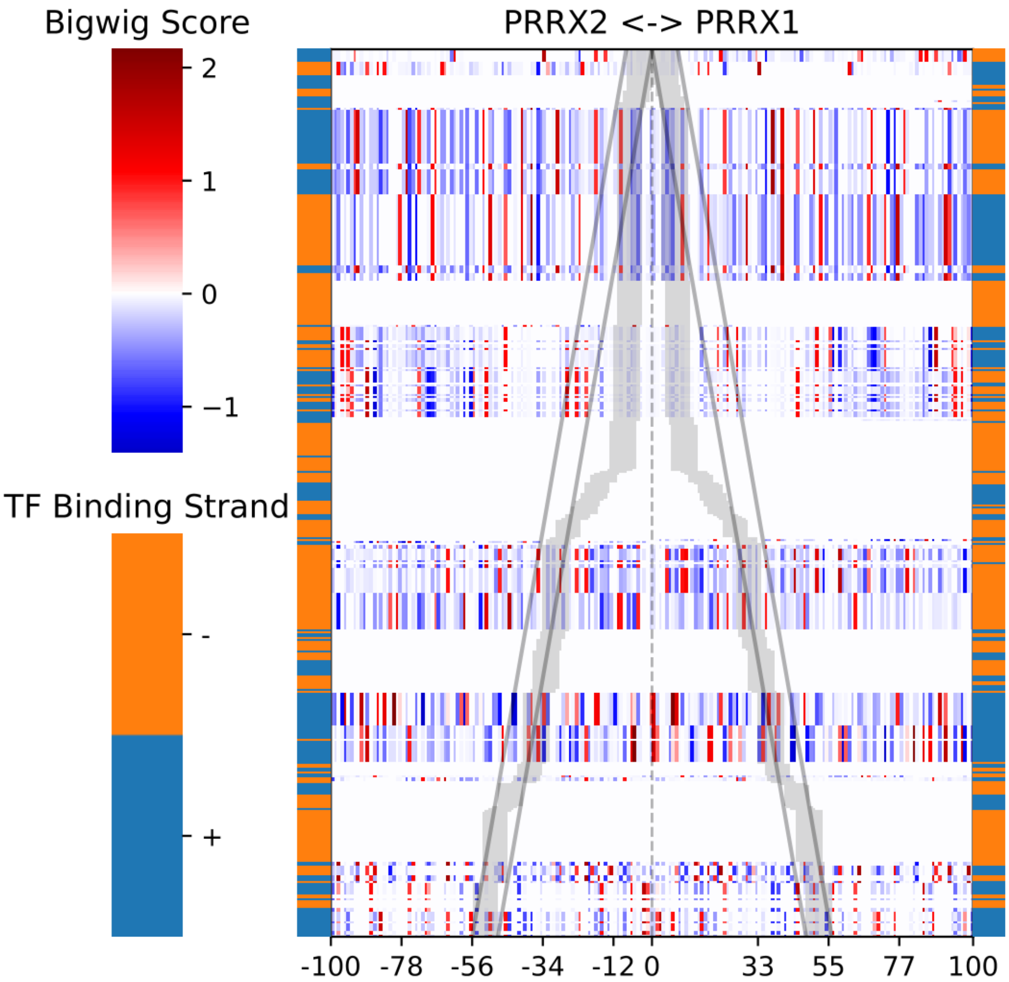

Bigwig Score
2
1
0
−1
TF Binding Strand
-
+
PRRX2 <-> PRRX1
-100
-78
-56
-34
-12
0
33
55
77
100

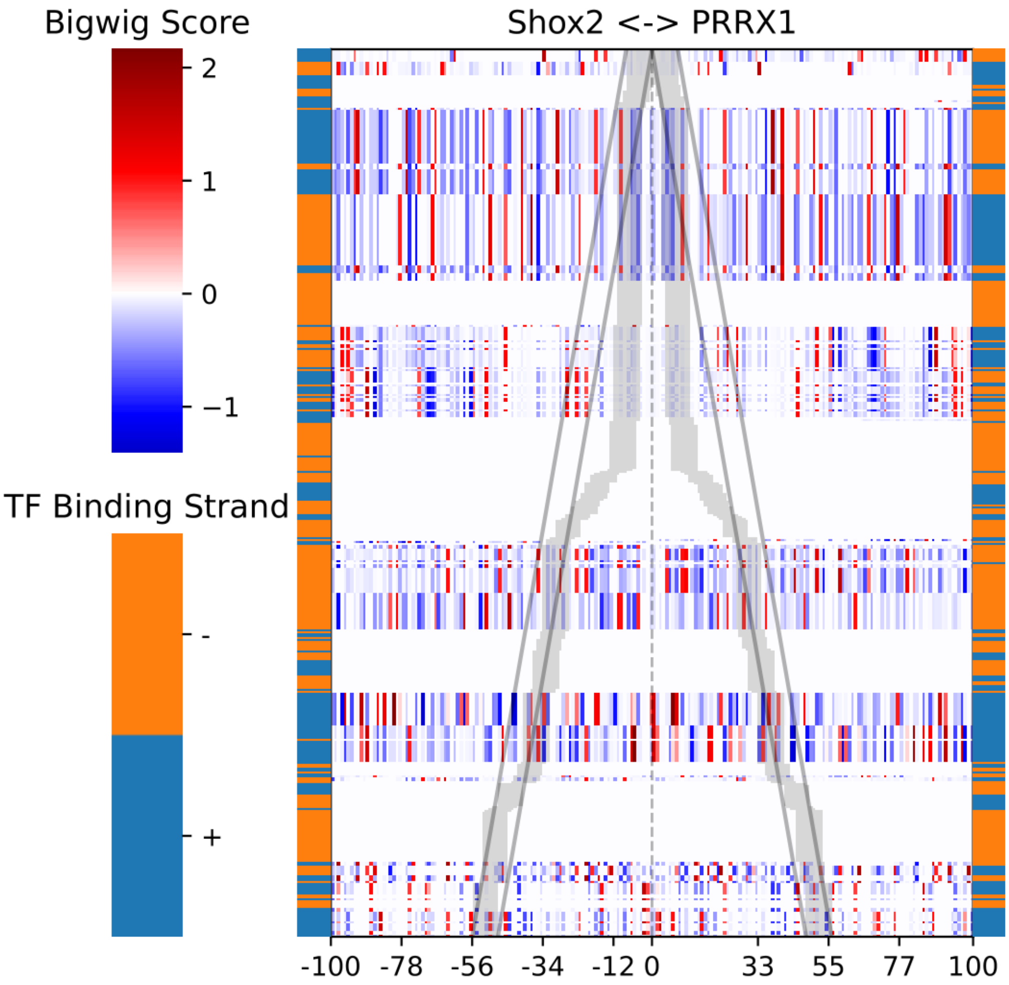
Bigwig Score
2
1
0
−1
TF Binding Strand
-
+
Shox2 <-> PRRX1
-100
-78
-56
-34
-12
0
33
55
77
100

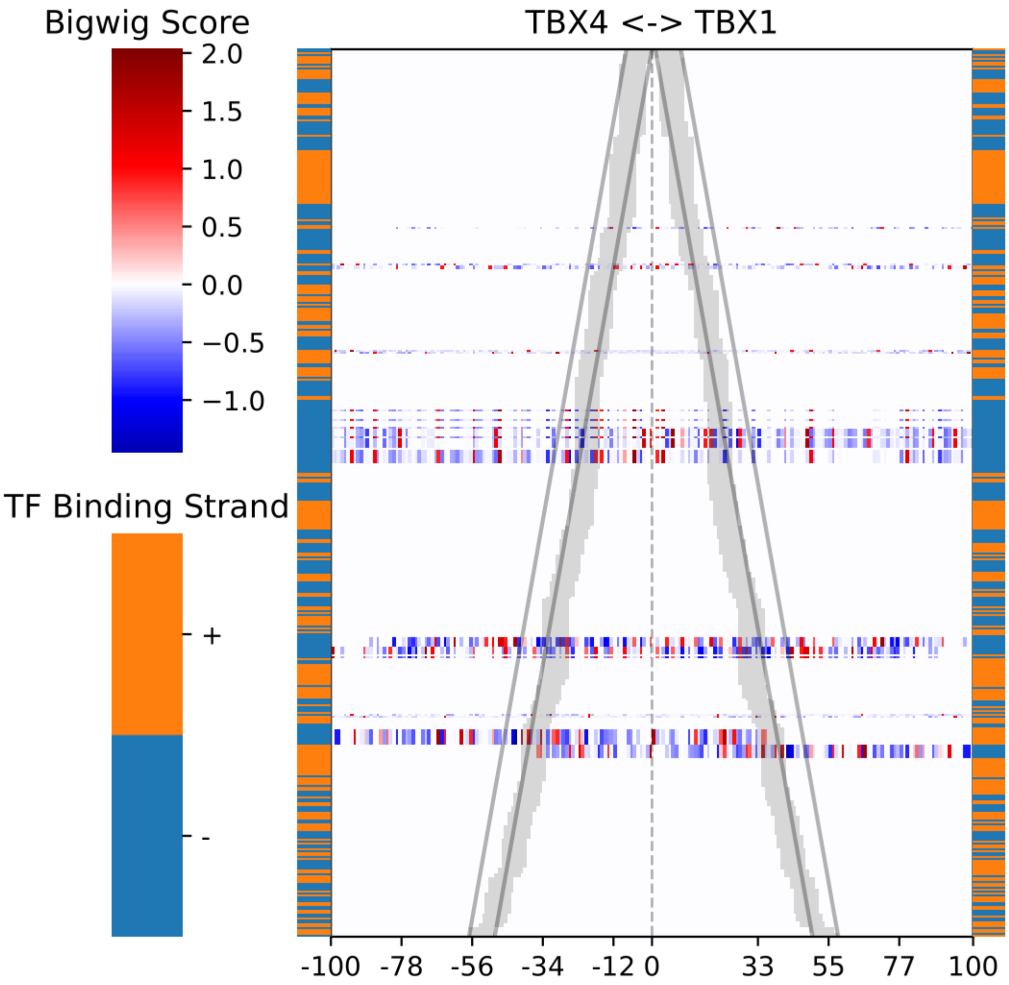

Bigwig Score
2.0
1.5
1.0
0.5
0.0
−0.5
−1.0
TF Binding Strand
+
-
TBX4 <-> TBX1
-100
-78
-56
-34
-12
0
33
55
77
100

Supplemental Information: Common TF pairs for Glia non-user (merged)

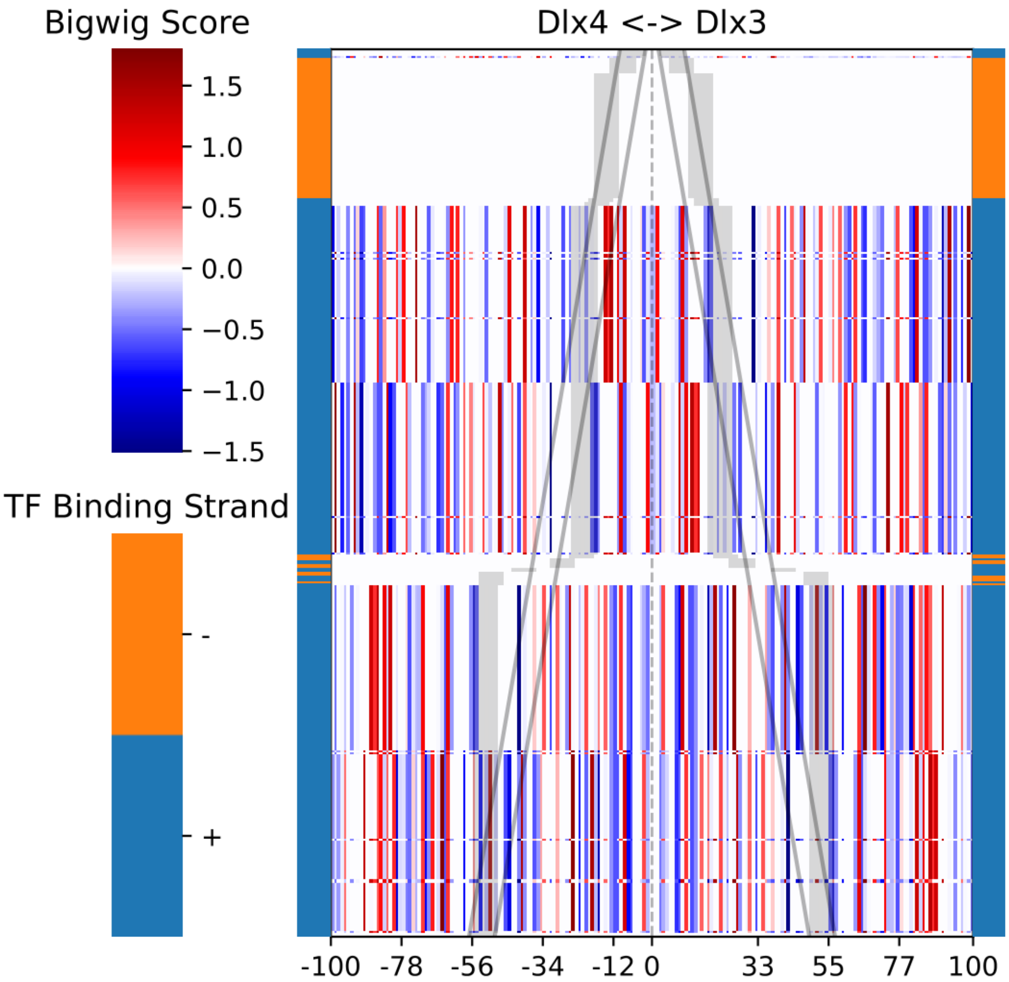

Bigwig Score
1.5
1.0
0.5
0.0
−0.5
−1.0
−1.5
TF Binding Strand
-
+
Dlx4 <-> Dlx3
-100
-78
-56
-34
-12
0
33
55
77
100

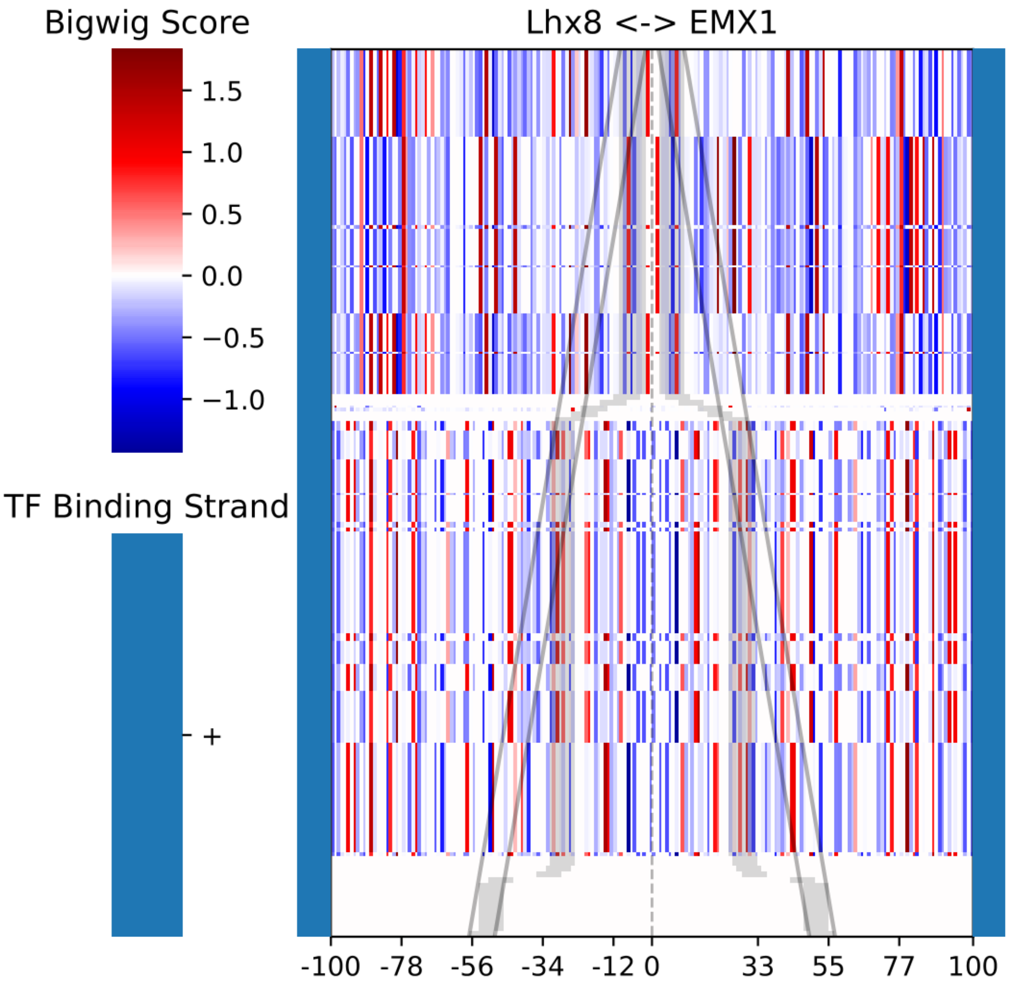

Bigwig Score
1.5
1.0
0.5
0.0
−0.5
−1.0
TF Binding Strand
+
Lhx8 <-> EMX1
-100
-78
-56
-34
-12
0
33
55
77
100

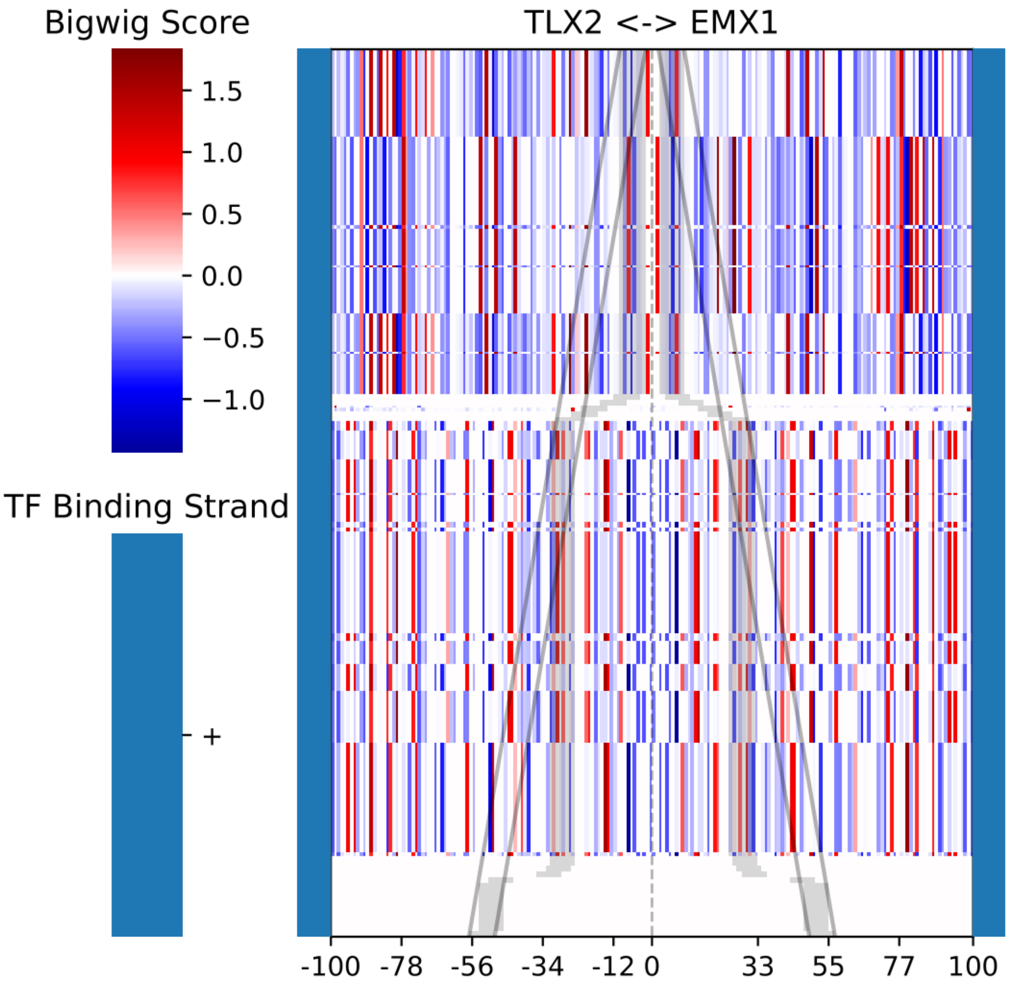
Bigwig Score
1.5
1.0
0.5
0.0
−0.5
−1.0
TF Binding Strand
+
TLX2 <-> EMX1
-100
-78
-56
-34
-12
0
33
55
77
100

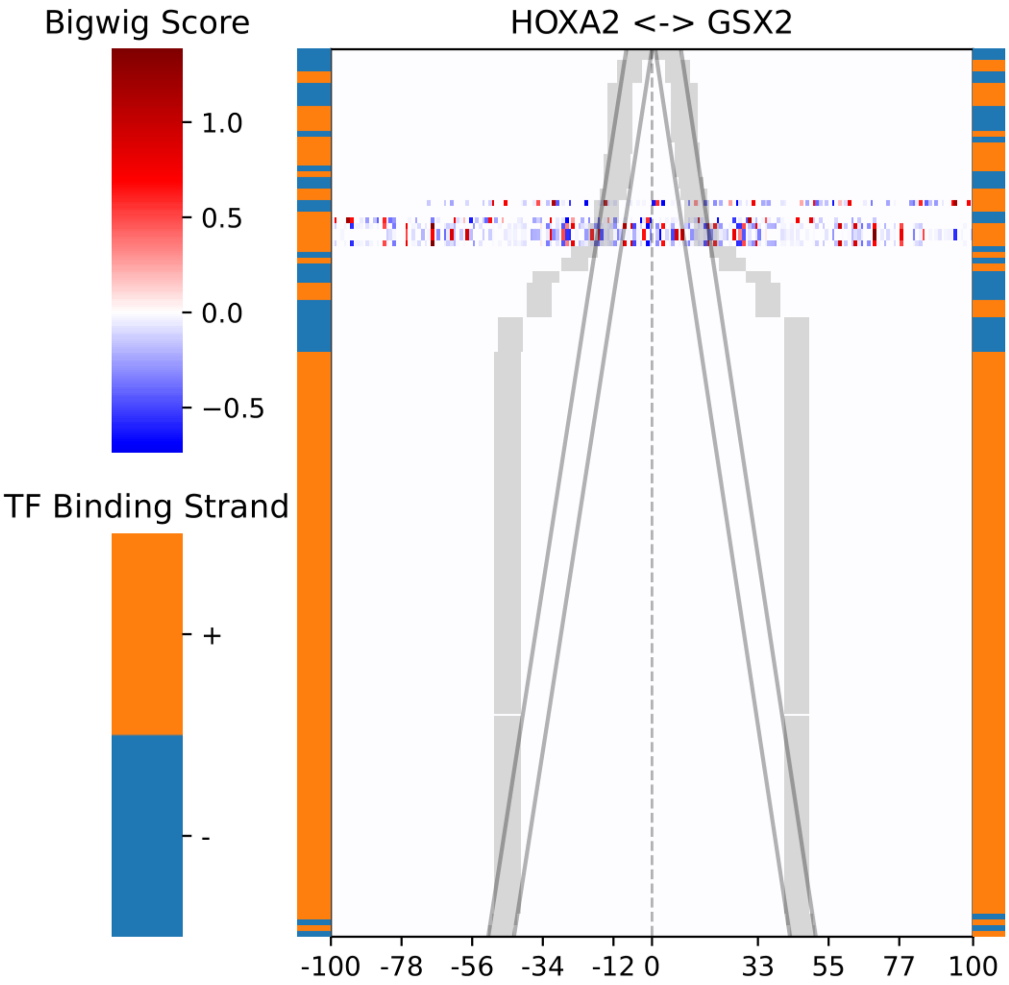

Bigwig Score
1.0
0.5
0.0
−0.5
TF Binding Strand
+
-
HOXA2 <-> GSX2
-100
-78
-56
-34
-12
0
33
55
77
100

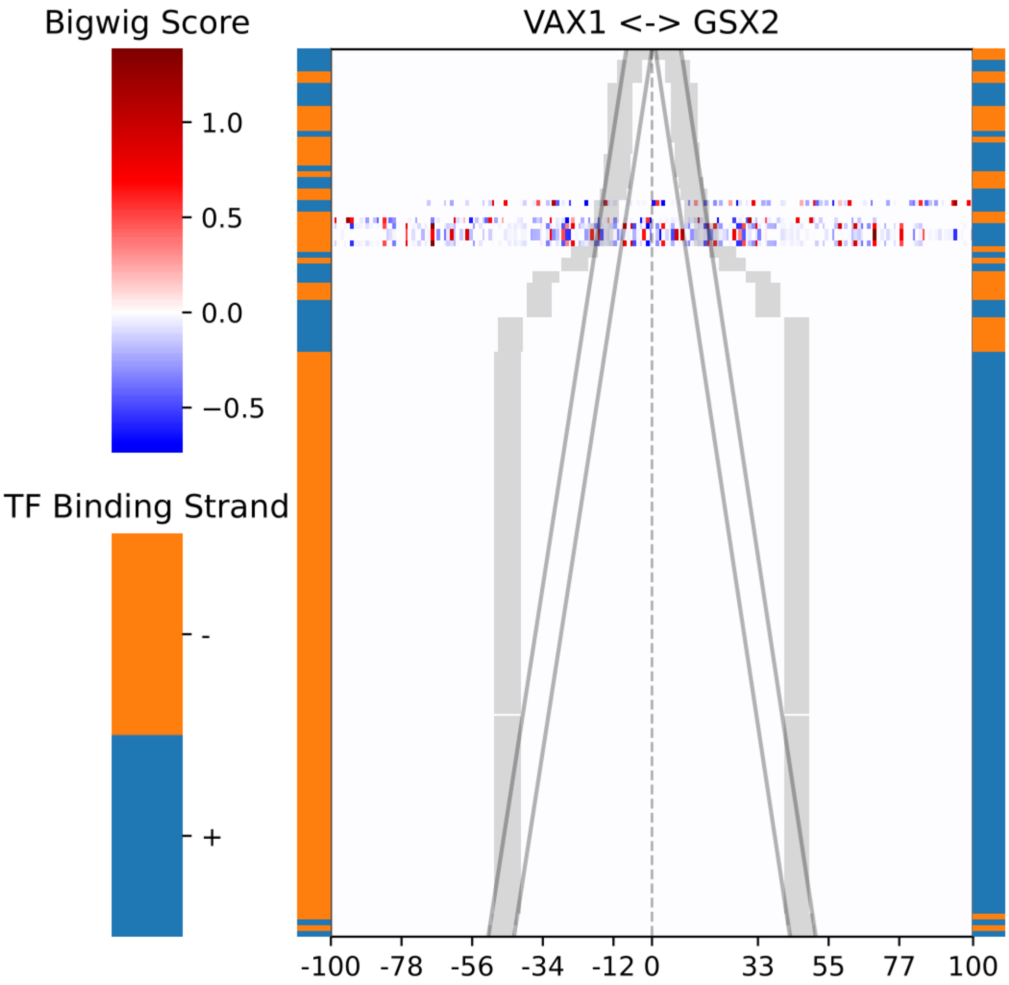

Bigwig Score
1.0
0.5
0.0
−0.5
TF Binding Strand
-
+
VAX1 <-> GSX2
-100
-78
-56
-34
-12
0
33
55
77
100

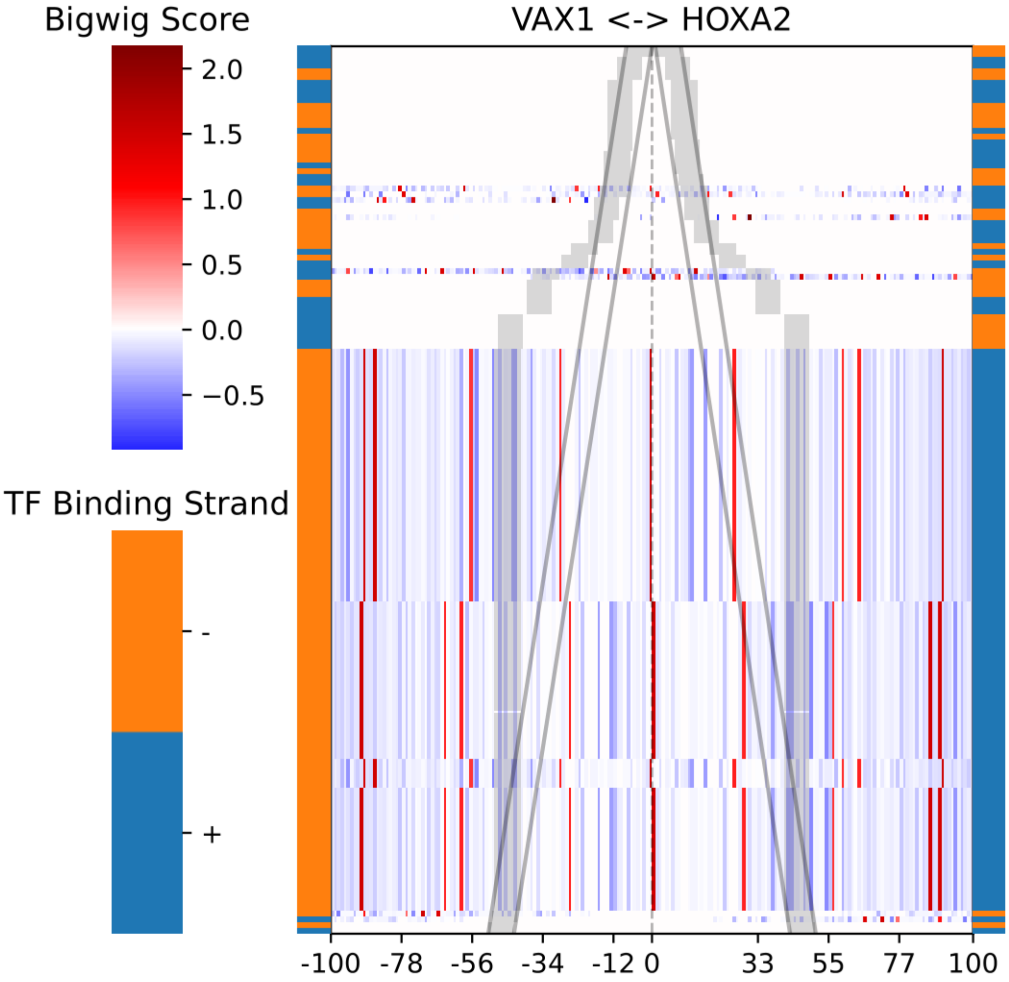
Bigwig Score
2.0
1.5
1.0
0.5
0.0
−0.5
TF Binding Strand
-
+
VAX1 <-> HOXA2
-100
-78
-56
-34
-12
0
33
55
77
100

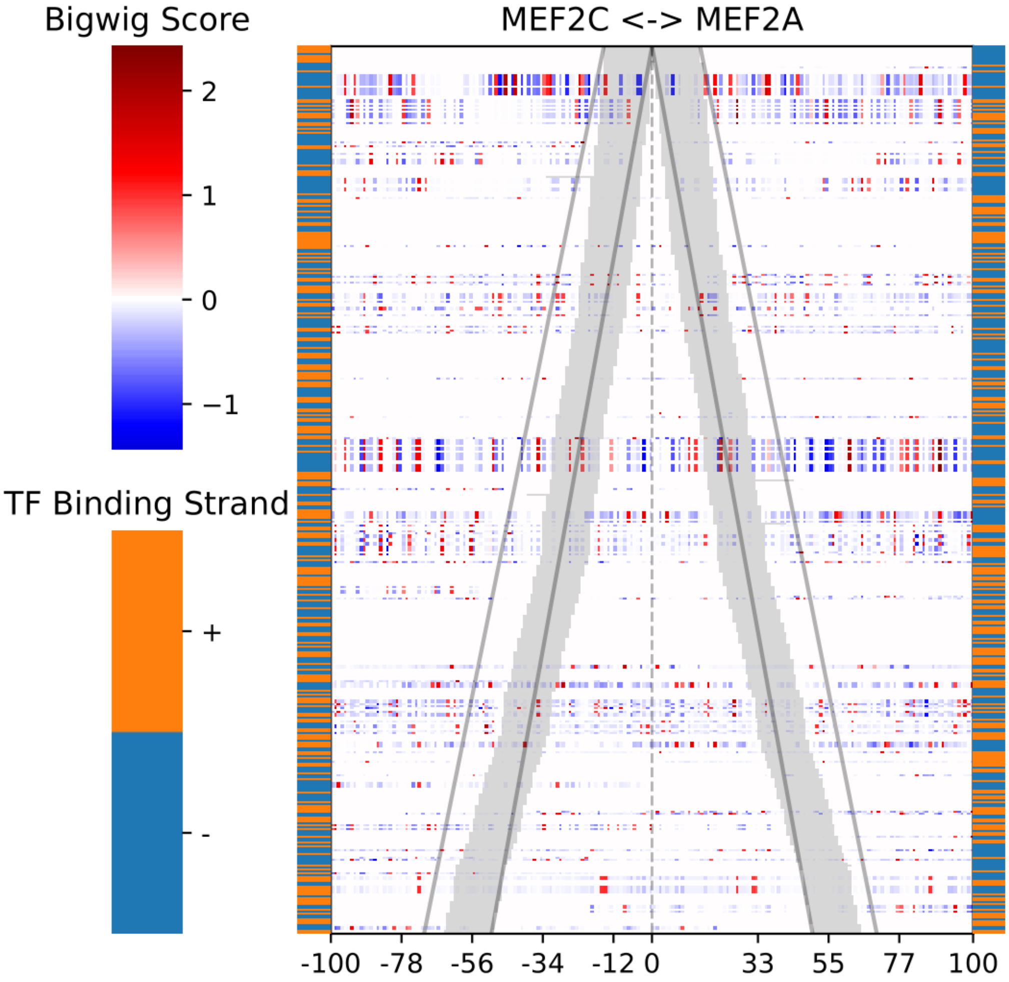

Bigwig Score
2
1
0
−1
TF Binding Strand
+
-
MEF2C <-> MEF2A
-100
-78
-56
-34
-12
0
33
55
77
100

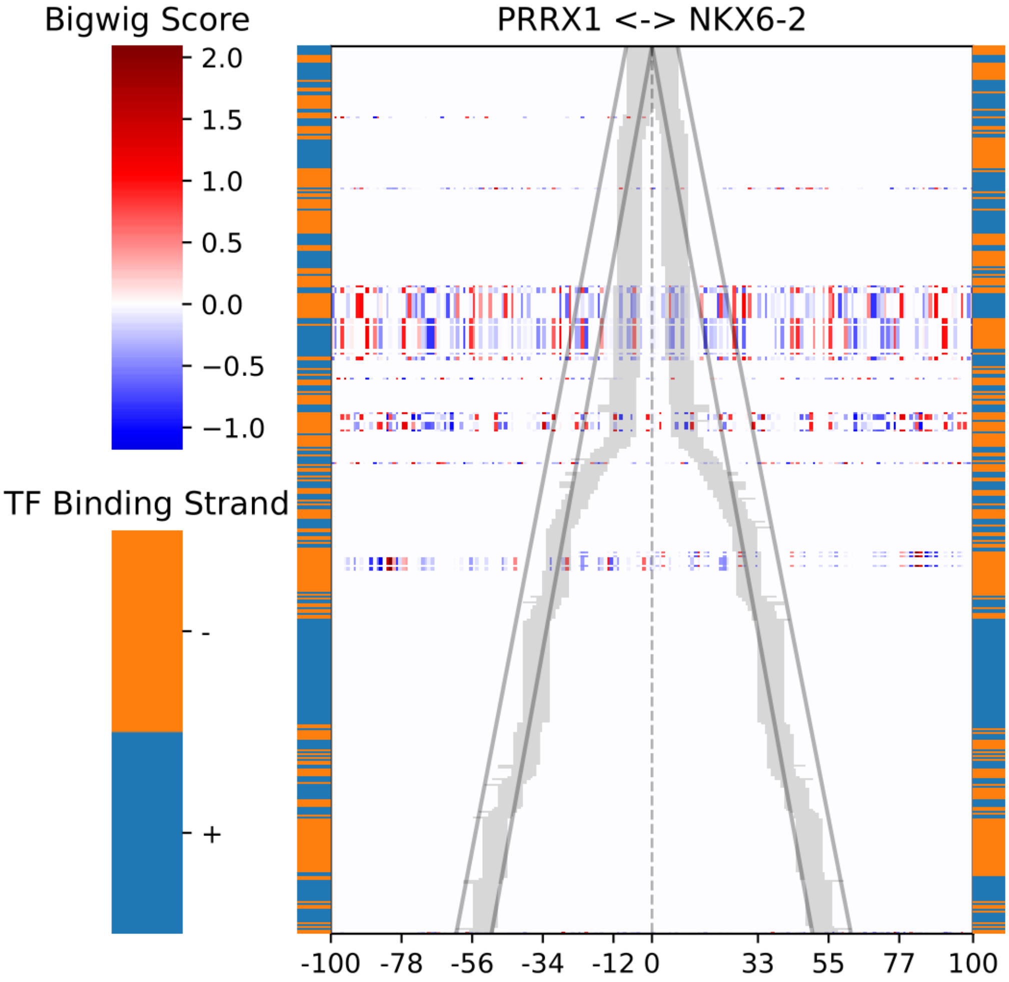

Bigwig Score
2.0
1.5
1.0
0.5
0.0
−0.5
−1.0
TF Binding Strand
-
+
PRRX1 <-> NKX6-2
-100
-78
-56
-34
-12
0
33
55
77
100

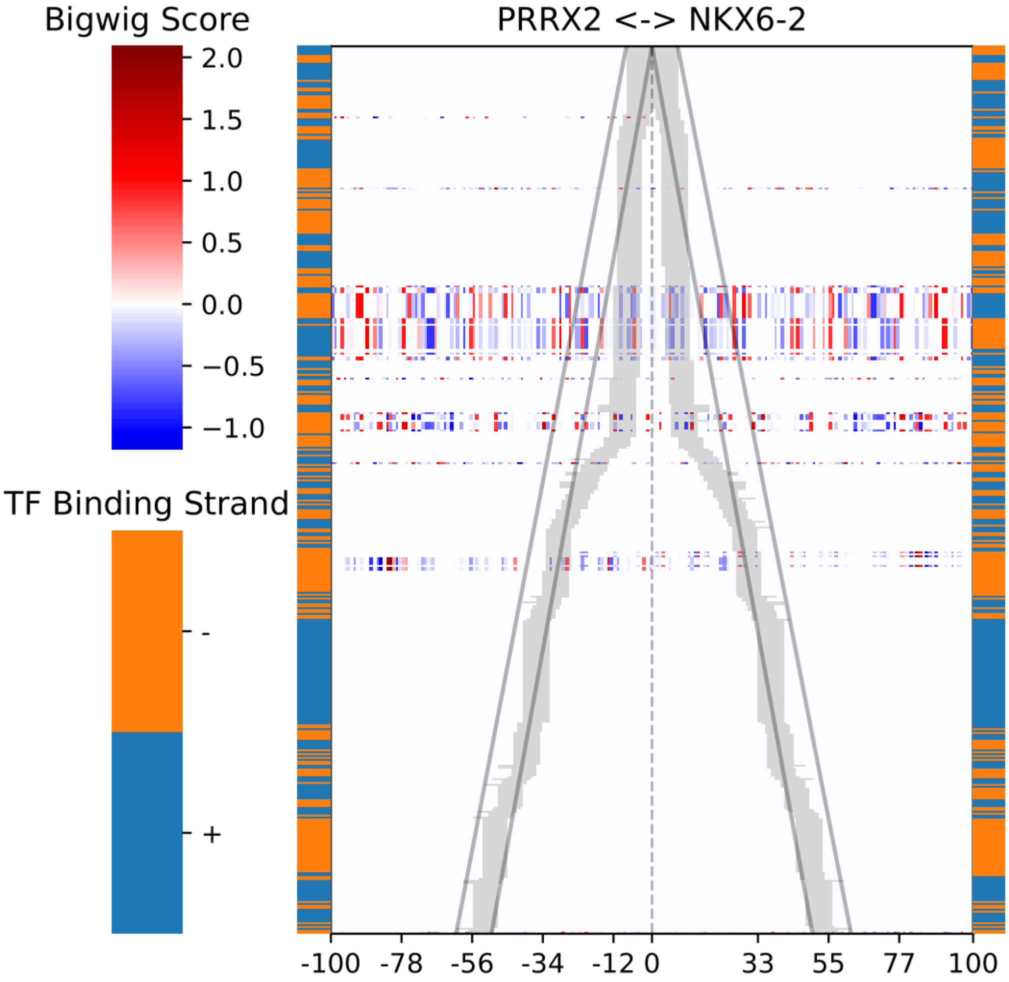

Bigwig Score
2.0
1.5
1.0
0.5
0.0
−0.5
−1.0
TF Binding Strand
-
+
PRRX2 <-> NKX6-2
-100
-78
-56
-34
-12
0
33
55
77
100

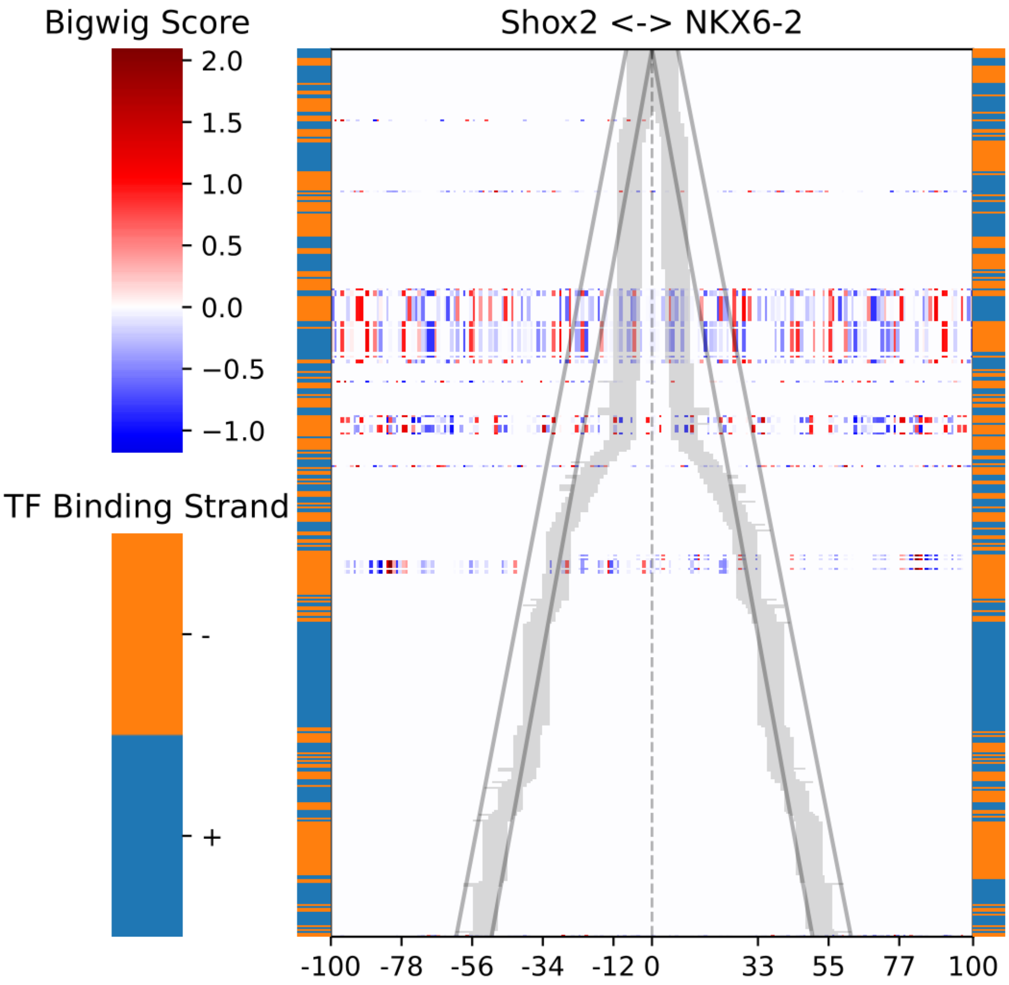

Bigwig Score
2.0
1.5
1.0
0.5
0.0
−0.5
−1.0
TF Binding Strand
-
+
Shox2 <-> NKX6-2
-100
-78
-56
-34
-12
0
33
55
77
100

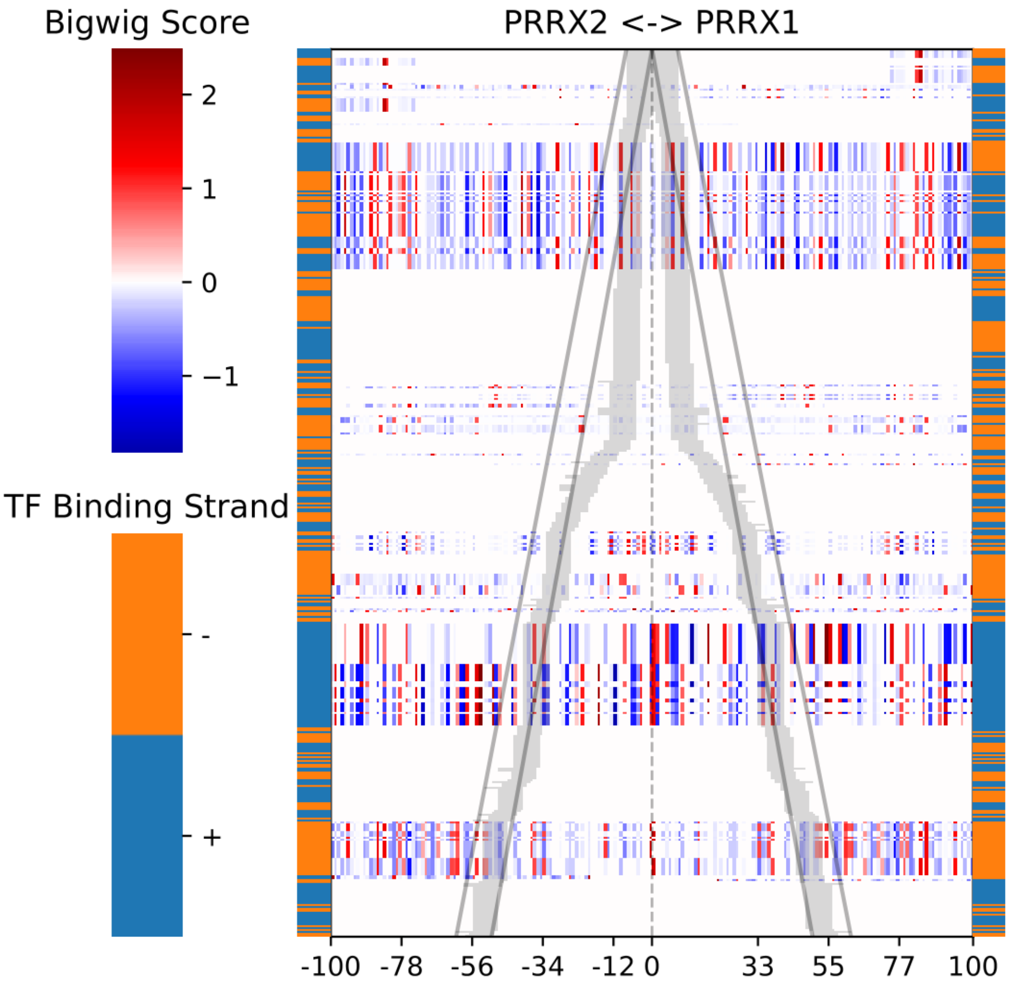
Bigwig Score
2
1
0
−1
TF Binding Strand
-
+
PRRX2 <-> PRRX1
-100
-78
-56
-34
-12
0
33
55
77
100

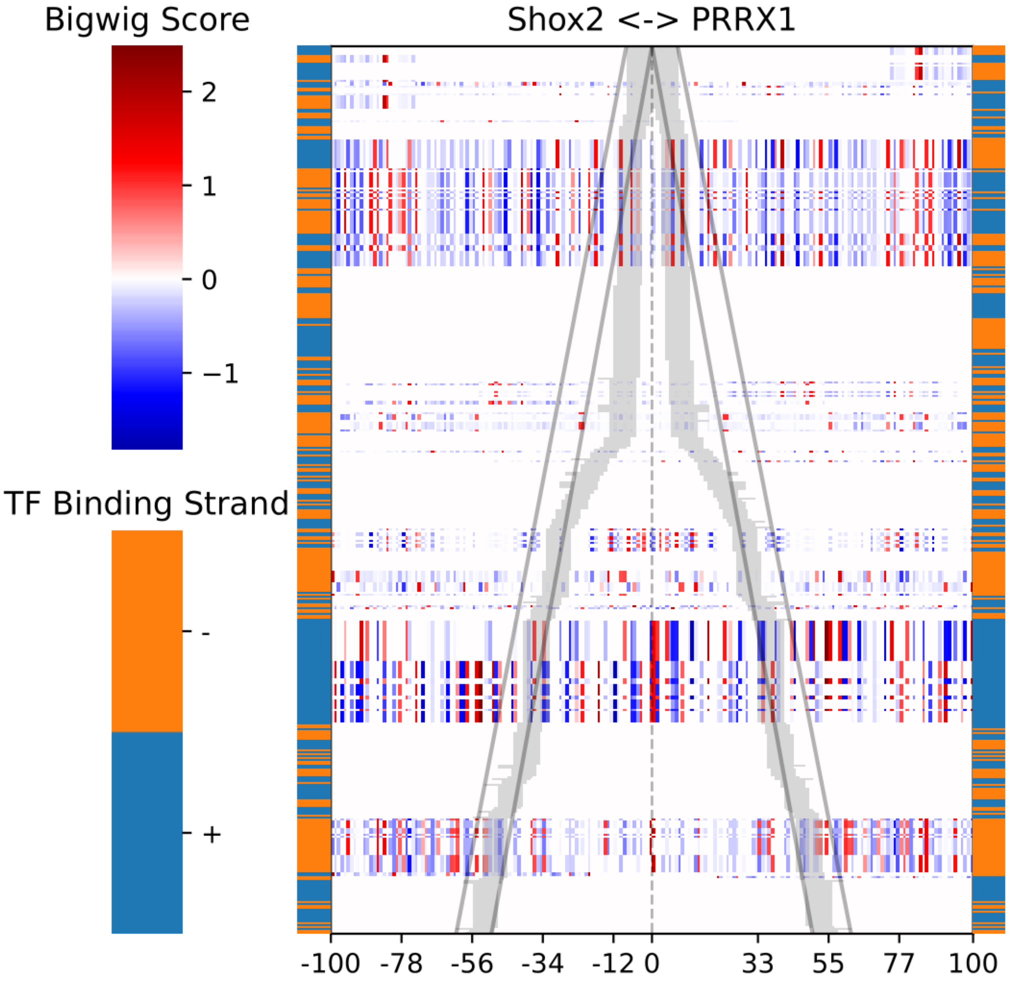
Bigwig Score
2
1
0
−1
TF Binding Strand
-
+
Shox2 <-> PRRX1
-100
-78
-56
-34
-12
0
33
55
77
100

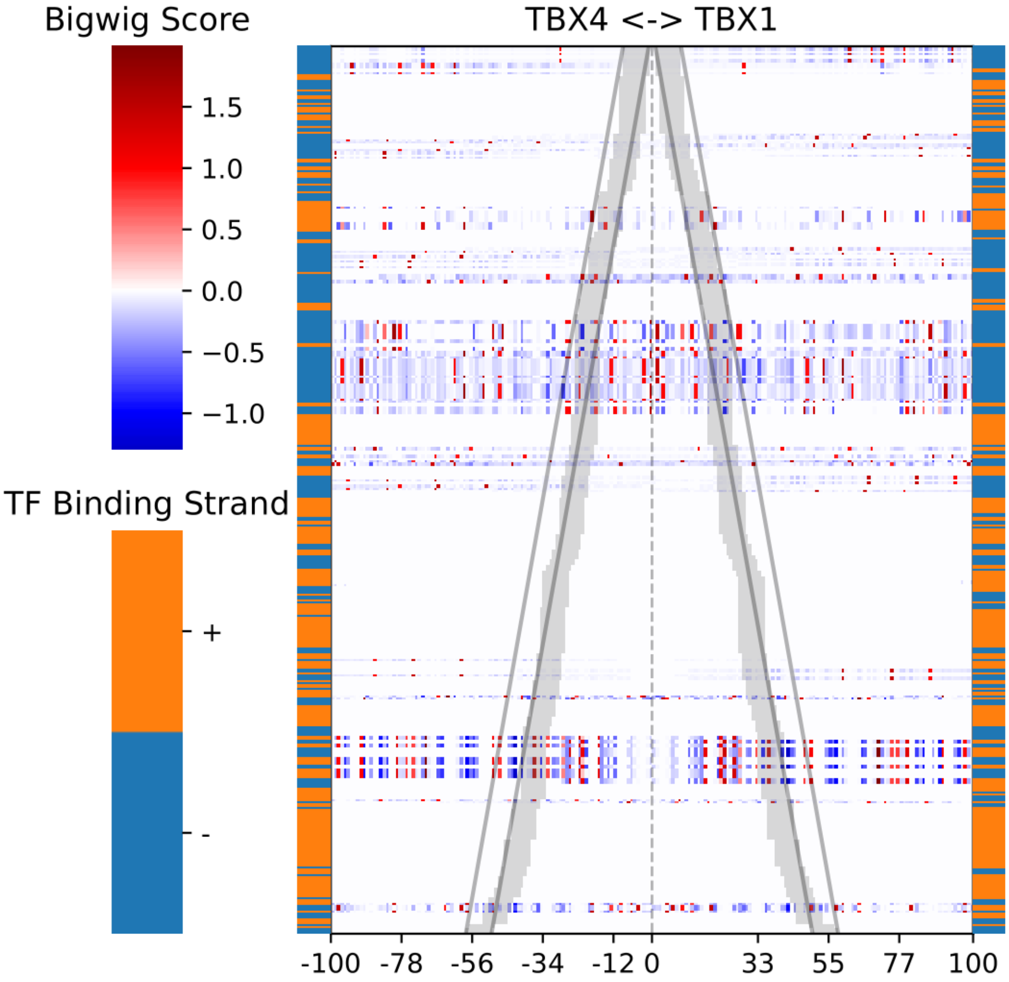
Bigwig Score
1.5
1.0
0.5
0.0
−0.5
−1.0
TF Binding Strand
+
-
TBX4 <-> TBX1
-100
-78
-56
-34
-12
0
33
55
77
100
