# Supplementary material for: Heroin addiction modulates transcription factor binding in regulatory regions of the human putamen
Source: Sci Rep. 2026 May 12;16:21737. doi: 10.1038/s41598-026-52754-7 (PMC13357565; doi:10.1038/s41598-026-52754-7)
Supplement: Supplementary file 4 — Supplementary Material 3 [file 41598_2026_52754_MOESM4_ESM.docx]

**Supplementary Figure 1.** Workflow outlining the analysis pipeline for studying TF binding grammar and unique TF binding sites extraction in OUD and control putamen tissues.

**Supplementary Figure 2. Differential TF Binding in Glia and Neurons and Unique Binding Profiles in Heroin Users only**. **(A, B)** Differential TF binding across glia and neurons after 1^st^ and 99^th^ percentile and p-value <0.05 filter criteria. **(C)** Comparison of top 5 TFs by unique binding sites, promoters, and enhancers in glia of heroin users vs non-users.

**Supplementary Figure 3. TF Interaction Networks and Pathway Enrichment in Glia and Neurons. (A)** Circular network displaying interactions between TFs, SUDs associated molecules (genes) and key signaling pathways in glia of heroin users, with lines indicating regulatory relationships. Solid lines represent direct interactions; dashed lines indicate expression. **(B)** Interaction network centered on MYC and STAT3 in glial cells, highlighting IL-33 and serotonin receptor signaling pathways; pink arrows connect USP17L27 to CSF1, STAT3 and MYC, illustrating their roles in cellular signaling and gene regulation. **(C-F)** Bar plots showing top enriched biological pathways with log10(padj) values for users and non-users in neuron and glia respectively. **[**GT: glia of users, GC: glia of non-users, NT: neurons of users, NC: neurons of non-users]

**Supplementary Figure 4. Comparative Analysis of TF Pair Co-Occurrence, Filtered TF Counts, and Regulatory Networks in Neurons of Heroin Users. (A)** Bar plot showing the total number of co-occurring TF pairs for neuron and glia categories. Each bar represents the total co-occurrence across all pairs [GT: glia of users, GC: glia of non-users, NT: neurons of users, NC: neurons of non-users]. **(B)** Bar plot depicting the number of filtered TFs for neuron and glia categories (GC, GT, NC, NT). A trendline (red) is added to show the filtered TF counts across categories.

**Supplementary Figure 5. Unique co-occurring TF Pair Prediction and Pathway Analysis in Non-User Glia. (A)** Scatter plots comparing cosine similarity and Z-scores for unique co-occurring TF pairs in glia of non-users (GC). **(B)** Network illustrating interactions between unique co-occurring TFs and pathways related to signaling in glia of non-users.

**Supplementary Figure 6. Unique co-occurring TF Pair Prediction and Pathway Analysis in User Neuron. (A)** Scatter plots comparing cosine similarity and Z-scores for unique co-occurring TF pairs in neurons of users (NT). **(B)** Network illustrating interactions between unique co-occurring TFs and pathways related to signaling in neurons of users.

**Supplementary Figure 7. Binding Patterns of common TF pair VAX1 and HOXA2 in Glia and Neurons (A-D).** PairMaps of common co-occurring TF pairs VAX1 and HOXA2 across neurons of non-users **(A)**, neurons of users **(B)**, glia of non-users **(C)**, and glia of users **(D)**. Bigwig scores (log-transformed, color-coded from red to blue) indicate TF binding, with strands (+ and -) represented by orange and blue color bars (y-axis). The x-axis shows the genomic positions (bp) from -50 to 150 bp around binding sites.
